# Effect of Steric Constraint at the *γ*-Backbone Position on the Conformations and Hybridization Properties of PNAs

**DOI:** 10.4061/2011/652702

**Published:** 2011-05-25

**Authors:** Matthew J. Crawford, Srinivas Rapireddy, Raman Bahal, Iulia Sacui, Danith H. Ly

**Affiliations:** Department of Chemistry, Center for Nucleic Acids Science and Technology (CNAST), Mellon Institute, Carnegie Mellon University, 4400 Fifth Avenue, Pittsburgh, PA 15213, USA

## Abstract

Conformationally preorganized peptide nucleic acids (PNAs) have been synthesized through backbone modifications at the *γ*-position, where R = alanine, valine, isoleucine, and phenylalanine side chains. The effects of these side-chains on the conformations and hybridization properties of PNAs were determined using a combination of CD and UV-Vis spectroscopic techniques. Our results show that the *γ*-position can accommodate varying degrees of sterically hindered side-chains, reaffirming the bimodal function of PNAs as the true hybrids of “peptides” and “nucleic acids.”

## 1. Introduction

Oligonucleotides are becoming increasingly important in the postgenomic era, as molecular tools for basic research as well as potential therapeutic and diagnostic reagents for the treatment and detection of genetic diseases [1–4]. However, for many of the *in vivo* applications, it is not sufficed just to be able to design oligonucleotide reagents that can recognize and bind sequence specifically to DNA or RNA. These reagents would also need to be able to get into cells and withstand enzymatic degradation by nucleases in the cellular *milieu*. To date, diverse classes of oligonucleotide analogues have been developed, but none possesses all the characteristic features [5–8]. It is, therefore, important to be able to modify the structures and/or chemical functionalities of these reagents further, with ease and flexibility, so that many of these desired features could be augmented and/or improved upon [9, 10] and undesired attributes, such as nonspecific binding and toxicity, could be further minimized [11, 12].

A particular class of oligonucleotide analogue endowed with such synthetic flexibility is peptide nucleic acids (PNAs) [13]. PNAs are nucleic acid mimics, comprised of *N*-(2-aminoethyl) glycine backbone and DNA/RNA nucleobases that are connected through a flexible carboxymethylene linker. Despite the structural departure from the natural biopolymers, PNAs maintain the ability to hybridize to complementary DNA and RNA strands through Watson-Crick base-pairing, just as their natural counterparts, but with higher affinity and sequence selectivity. The improvement in binding affinity has been attributed in part to the lack of electrostatic repulsion in the backbones [14], while the enhancement in sequence selectivity has been attributed in part to the increased backbone rigidity upon hybridization as the result of solvation [15, 16]. Unlike DNA or RNA, which are prone to nucleolytic degradation, PNAs are resistant to both proteases and nucleases. These properties, together with the ease and flexibility of synthesis, make PNAs an attractive commodity for *in vivo* applications. 

So far, a large number of structural modifications have been made to the backbone of PNAs [17–21]. Among them, modifications made at the *γ*-position show the most promise because of the simplicity and flexibility in synthesis and the benefits that they confer on the hybridization properties of PNAs [22–28]. Recently, we showed that randomly folded, single-stranded PNAs can be preorganized into either a right-handed or left-handed helix by installing an appropriate stereogenic center at the *γ*-backbone position [27–29]. *γ*PNAs derived from L-amino acids adopted a right-handed helix, while those derived from D-amino acids adopted a left-handed helix (unpublished data); however, only the right-handed helical *γ*PNAs are able to hybridize to DNA and RNA with high affinity and sequence selectivity. Although a number of amino acid side chains including alanine [22, 23], serine [27], cysteine [25], and lysine [24, 26, 30] have been incorporated at this position, a systematic study aimed at assessing the effect of steric hindrance on the conformations and hybridization properties of PNAs has not yet been established. Knowledge of this information is crucial to the future design of PNAs with improved hybridization properties, water solubility, cellular uptake, biodistribution, and pharmacokinetics—all of which are essential for their *in vivo* applications. In an attempt to address this issue, we synthesized a series of thymine-containing *γ*PNA monomers and corresponding oligomers with different amino acid side chains at the *γ*-backbone position and characterized their conformations and hybridization properties using a combination of CD and UV-Vis spectroscopic techniques.

## 2. Materials and Methods

### 2.1. Materials and Equipments

All commercial reagents were used without further purification. Solvents were dried by standard methods and distilled immediately prior to use. All chemicals were purchased from Aldrich except for Boc-Val-OH, Boc-Phe-OH, Boc-Ala-OH, and Boc-Ile-OH, which were purchased from Novabiochem. ^1^H-NMR and ^13^C-NMR spectra were recorded on a Bruker Avance AV-300 NMR spectrometer using standard Bruker software. Column chromatography was performed using standard grade silica gel from Sorbent Technologies. TLC was performed with silica gel 60 F-254 precoated plates from Sorbent Technologies. MALDI-TOF experiments were performed on a PerSeptive Biosystems Voyager STR MALDI-TOF mass spectrometer using a 10 mg/mL solution of *α*-hydroxycinnamic acid in ACN-water (1 : 1) with 0.1% TFA. Mass spectra were recorded on a Finnigan LCQ ESI/APCI ion trap mass spectrometer by electrospray ionization. CD experiments were performed on a Jasco J-715 spectropolarimeter equipped with a thermoelectrically controlled single-cell holder. UV-Vis measurements were taken on a Varian Cary 300 Bio spectrophotometer equipped with a thermoelectrically controlled multicell holder. All Boc/Z-protected PNA monomers were purchased from Applied Biosystems. PNA1–9 oligomers were synthesized on solid-support according to standard protocols [31]. The oligomers were purified by reverse-phase HPLC and characterized by MALDI-TOF. All PNA stock solutions were prepared using nanopure water, and the concentrations were determined at 95°C using the following extinction coefficients for PNA monomers: 13,700 M^−1^ cm^−1^ (**A**), 6,600 M^−1^ cm^−1^ (**C**), 11,700 M^−1^ cm^−1^ (**G**), and 8,600 M^−1^ cm^−1^ (**T**). The DNA oligomers were purchased from Integrated DNA Technologies, Inc. and used without further purification.

### 2.2. CD Analysis

The samples were prepared in buffer containing 0.1 mM EDTA, 100 mM NaCl, 10 mM sodium phosphate at pH 7.4. The UV absorption at 260 nm was carefully adjusted at 95°C so that each sample contained the same concentration. All spectra represent an average of at least twelve scans between 200–320 nm, measured at the rate of 100 nm per min in a 1-cm path-length cuvette at 25°C. The spectra were baseline corrected and then smoothed using an eight-point adjacent averaging algorithm.

### 2.3. Thermal Denaturation Experiments

The samples were prepared in the same buffer employed in the CD experiments. UV-Vis absorbance at 260 nm was recorded every 0.5°C as the samples were cooled from 95°C to 20°C and then heated to 95°C at a rate of 1.0°C/min. Each *T*_*m*_ curve was then normalized for absorbance reading.

### 2.4. Monomer Synthesis

Alanine derivatives **1**–**5a** were prepared according to published procedures [27]. **5a**: HRMS (ESI/MS_m/z_) M_calc _ for C_17_H_26_N_4_NaO_4_ 421.17, found 412.1344.

**Boc-Val-ol (1b)**

Boc-Val-OH (501 mg, 2.31 mmoL) was dissolved in a cold (−15°C) solution of dimethoxyethane (DME) (5 mL). N-methyl morpholine (NMM) (0.25 mL, 2.27 mmoL) and isobutyl chloroformate (0.30 mL, 2.30 mmoL) were added dropwise to the mixture. The solution was allowed to stir for 1 min. The precipitated N-methyl morpholine hydrochloride was removed by vacuum filtration and washed with DME (3 × 2 mL). The filtrate and washing solutions were recombined, cooled to −15°C in a methanol ice bath. To this mixture, a solution of NaBH_4_ (0.130 g, 3.45 mmoL) in water (0.5 mL) was added dropwise (producing evolution of gas). The mixture was allowed to stir for 1 min, and approximately 100 mL of water was added to quench the reaction. The solution was then transferred to a separatory funnel and extracted with ethyl acetate (EtOAc) (3 × 50 mL) and then brine (1 × 100 mL). The organic layers were combined and dried over anhydrous sodium sulfate. The solvent was then removed under reduced pressure to give 450 mg (2.21 mmoL, 97% yield) of the desired product **1b **as pale yellow oil. TLC: rf = 0.65 (6 : 4 EtOAc : hexane). ^1^H-NMR (CDCl_3_, 300 MHz): *δ* = 0.80–1.06 (6H, m, CH(CH_3_)_2_); 1.49 (9H, s, C(CH_3_)_3_); 1.85 (1H, m, CH(CH_3_)_2_); 2.25 (1H, br, CH_2_OH); 3.50 (1H, m, NCHCO); 3.76 (2H, m, CH_2_OH); 4.65 (1H, br, CONH). Minor solvent impurities were found in the ^1^H-NMR: EtOAc (1.30, 2.10, and 4.12), H_2_O (1.60), Acetone (2.20), and DME (3.40 and 3.60). (ESI-MS m/z) mass calculated 203.28 for C_10_H_21_NO_3_, found 226.07 (203.28 + Na^+^).

**Boc-Ile-ol (1c)**

Synthesis is analogous to that of **1b**, starting from Boc-Ile-OH (499.6 mg, 2.08 mmoL), NMM (0.23 mL, 2.09 mmoL), isobutyl chloroformate (0.27 mL, 2.08 mmoL), and NaBH_4_ (0.12 g, 3.17 mmoL). 430 mg (1.99 mmoL, 95% yield) of **1c** was obtained as colorless oil. TLC: rf = 0.65 (6 : 4 EtOAc : hexane). ^1^H-NMR (CDCl_3_, 300 MHz): *δ* = 0.80–1.00 (6H, m, CHCH_3_, CHCH_2_CH_3_); 1.20–1.40 (2H, m, CHCH_2_CH_3_); 1.49 (9H, s, C(CH_3_)_3_); 1.60 (1H, m, CHCH_2_CH_3_); 2.25 (1H, br, CH_2_OH); 3.50 (1H, m, NCHCO); 3.76 (2H, m, CH_2_OH); 4.65 (1H, br, CONH). Minor impurities were found in the ^1^H-NMR: Isobutyl chloroformate (0.9, 2.0, 3.90), EtOAc (1.30, 2.10, and 4.12), and H_2_O (1.60). (ESI-MS m/z) mass calculated 217.31 for C_11_H_23_NO_3_, found 240.31 (217.31 + Na^+^).

**Boc-Phe-ol (1d)**

Synthesis is analogous to that of **1b**, starting from Boc-Phe-OH (500 mg, 1.89 mmoL), NMM (0.21 mL, 1.91 mmoL), isobutyl chloroformate (0.25 mL, 1.93 mmoL), and NaBH_4_ (0.11 g, 2.91 mmoL). 410 mg (1.63 mmoL, 86% yield) of **1d** was obtained as colorless oil. TLC: rf = 0.65 (6 : 4 EtOAc : hexane). ^1^H-NMR (CDCl_3_, 300 MHz): *δ* = 1.49 (9H, s, C(CH_3_)_3_); 2.25 (1H, br, CH_2_OH); 2.85 (2H, m, CHCH_2_Ph); 3.50 (1H, m, NCHCO); 3.76 (2H, m, CH_2_OH); 4.75 (1H, br, CONH); 7.25–7.40 (5H, m, CH_2_PhH2–6). Minor impurities were found in the ^1^H-NMR: Isobutyl chloroformate (0.90, 2.00, 3.90), EtOAc (1.30, 2.10, and 4.12), H_2_O (1.60), and Acetone (2.20). (ESI-MS m/z) mass calculated 251.32 for C_14_H_21_NO_3_, found 252.0 and 274.13 (251.32 + Na^+^).

**Ethyl *N*-(o-nitrophenylsulfonyl) glycinate (Nos-Gly-OEt)**

It was prepared following the reported procedures  [23].

**Boc-Val*ψ*[CH_2_N(Nos)]Gly-OEt (2b)**

**1b** (450 mg, 2.21 mmoL), Nos-Gly-OEt (475 mg, 1.65 mmoL), and triphenylphosphine (Ph_3_P) (653 mg, 2.49 mmoL) were dissolved in freshly distilled dry THF (25 mL). The solution was stirred in an ice bath under nitrogen. Diisopropyl azodicarboxylate (DIAD) (0.5 mL, 2.54 mmoL) was added dropwise over 10 min. The reaction was stirred under nitrogen overnight. The solvent was removed under a reduced pressure. The oily product was purified by column chromatography (3 : 7 EtOAc : hexane, rf = 0.65) to give 700 mg (1.48 mmoL, 90% yield) of **2b**. ^1^H-NMR (CDCl_3_, 300 MHz): *δ* = 0.90–1.00 (6H, m, CH(CH_3_)_2_); 1.25 (3H, t, OCH_2_CH_3_, J = 7.1 Hz); 1.49 (9H, s, C(CH_3_)_3_); 1.80 (1H, m, CH(CH_3_)_2_); 3.45–3.80 (3H, m, NCHCH_2_N); 4.00–4.20 (2H, m, OCH_2_CH_3_); 4.40–4.65 (2H, m, NCH_2_CO); 6.30 (1H, br, NH); 7.60–7.80 (3H, m, ArH4–6); 8.10 (1H, m, ArH3). Minor impurities were found in the ^1^H-NMR: DIAD byproduct (1.30, 5.00, 6.30), H_2_O (1.60), and Acetone (2.20). (ESI-MS m/z) mass calculated 473.54 for C_20_H_31_N_3_O_8_S, found 496.13(473.54 + Na^+^).

**Boc-Ile*ψ*[CH_2_N(Nos)]Gly-OEt (2c)**

Synthesis is analogous to that of **2b**, starting from **1c** (430 mg, 1.99 mmoL), Nos-Gly-OEt (436 mg, 1.51 mmoL), and Ph_3_P (604 mg, 2.30 mmoL). The reaction was stirred under nitrogen for 48 hrs. The solvent was removed, and the oily product mixture was purified by column chromatography (3 : 7 EtOAc : hexane, rf = 0.68) to give 649 mg (1.33 mmoL, 88% yield) of **2c**. ^1^H-NMR (CDCl_3_, 300 MHz): *δ* = 0.80–1.00 (6H, m, CHCH_3_, CHCH_2_CH_3_), 1.10 (2H, m, CHCH_2_CH_3_); 1.20 (3H, t, OCH_2_CH_3_, J = 7.1 Hz); 1.49 (9H, s, C(CH_3_)_3_); 1.60 (1H, m, CHCH_2_CH_3_); 3.45–3.80 (3H, m, NCHCH_2_N); 4.00–4.20 (2H, m, OCH_2_CH_3_); 4.40–4.65 (2H, m, NCH_2_CO); 6.30 (1H, br, NH); 7.60–7.80 (3H, m, ArH4–6); 8.10 (1H, m, ArH3). Minor impurities were found in the ^1^H-NMR: DIAD byproduct (1.30, 5.00, 6.30), H_2_O (1.60), and Acetone (2.20). (ESI-MS m/z) mass calculated 487.57 for C_21_H_33_N_3_O_8_S, found 510.57 (487.57 + Na^+^).

**Boc-Phe*ψ*[CH_2_N(Nos)]Gly-OEt (2d)**

Synthesis is analogous to that of **2b**, starting from **1d** (410 mg, 1.63 mmoL), Nos-Gly-OEt (402 mg, 1.39 mmoL), and Ph_3_P (528 mg, 2.01 mmoL). The reaction was stirred under nitrogen overnight. The solvent was removed, and the oily product mixture was purified by column chromatography (3 : 7 EtOAc : hexane, rf = 0.70) to give 560 mg (1.07 mmoL, 77% yield) of **2d**. ^1^H-NMR (CDCl_3_, 300 MHz): *δ* = 1.20 (3H, t, OCH_2_CH_3_ J=7.1 Hz); 1.49 (9H, s, C(CH_3_)_3_); 2.85 (2H, m, CHCH_2_Ph); 3.50–3.70 (3H, m, NCHCH_2_N); 4.02 (1H, m, CHCH_2_Ph); 4.30 (2H, m, NCH_2_CO); 6.30 (1H, br, NH); 7.10–7.40 (5H, m, CH_2_PhH2–6); 7.65–7.80 (3H, m, oNBS ArH4–6); 7.90 (1H, m, oNBS ArH3). Minor impurities were found in the ^1^H-NMR: DIAD byproduct (1.30, 5.00, 6.30), H_2_O (1.60), and Acetone (2.20). (ESI-MS m/z) mass calculated 521.58 for C_24_H_31_N_3_O_8_S, found 544.13 (521.58 + Na^+^).

**Boc-Val*ψ*(CH_2_N)Gly-OEt (3b)**

**2b** (700 mg, 1.48 mmoL) was dissolved in acetonitrile (25 mL) under nitrogen. Dried potassium carbonate (440 mg, 3.13 mmoL) was added to the solution. While stirring thiophenol (0.450 mL, 4.40 mmoL) was added dropwise over the course of 3 min. The mixture was vigorously stirred overnight. The resulting mixture was gravity-filtered to remove excess potassium carbonate and the solvent removed under reduced pressure. The crude product was dissolved in 100 mL EtOAc, washed with water (2 × 100 mL), followed by brine (1 × 50 mL). The organic layers were recombined and dried over anhydrous sodium sulfate. The solvent was removed under reduced pressure. The crude product was purified by column chromatography (20 : 1 EtOAc:EtOH, rf = 0.15) to give 200 mg (0.69 mmoL, 47% yield). ^1^H-NMR (CDCl_3_, 300 MHz): *δ* = 0.90–1.00 (6H, m, CH(CH_3_)_2_); 1.25 (3H, t, OCH_2_CH_3_, J = 7.1 Hz); 1.49 (9H, s, C(CH_3_)_3_); 1.80 (1H, m, CH(CH_3_)_2_); 2.65 (2H, m, CHCH_2_NH); 3.40 (2H, AB, NCH_2_CO, J=17.4 Hz); 3.50 (1H, m, NCHCH_2_); 4.10–4.30 (2H, q, OCH_2_CH_3_, J = 7.2 Hz); 4.60 (1H, br, Boc NH). ^13^C NMR (CDCl_3_, 75 MHz): *δ* = 172.5, 156.2, 77.2, 60.7, 55.5, 50.9, 30.5, 28.4, 19.3, 18.2, 14.2. (ESI-MS m/z) mass calculated 288.38 for C_14_H_28_N_2_O_4_, found 289.07.

**Boc-Ile*ψ*(CH_2_N)Gly-OEt (3c)**

Synthesis is analogous to that of **3b**, starting from **2c** (649 mg, 1.33 mmoL), potassium carbonate (426 mg, 3.08 mmoL), and thiophenol (0.420 mL, 4.11 mmoL). The reaction was stirred under nitrogen for 48 hrs. The solvent was removed under reduced pressure after gravity filtration. Analogous workup was performed as for compound **4b**. The crude product was purified by column chromatography (EtOAc, rf = 0.15) to give 300 mg (0.99 mmoL, 75% yield) of oily product. ^1^H-NMR (CDCl_3_, 300 MHz): *δ* = 0.80–1.00 (6H, m, CHCH_3_, CHCH_2_CH_3_), 1.15 (2H, m, CHCH_2_CH_3_); 1.30 (3H, t, OCH_2_CH_3_, J = 7.1 Hz); 1.49 (9H, s, C(CH_3_)_3_); 1.60 (1H, m, CHCH_2_CH_3_); 2.65 (2H, m, CHCH_2_NH); 3.40 (2H, AB, NCH_2_CO, J = 17.4 Hz); 3.70 (1H, m, NCHCH_2_); 4.10–4.30 (2H, q, OCH_2_CH_3_, J = 7.2 Hz); 4.60 (1H, br, Boc NH). ^13^C NMR (CDCl_3_, 75 MHz): *δ* = 172.5, 156.1, 77.2, 60.7, 54.5, 50.9, 50.3, 37.2, 28.4, 25.4, 15.2, 14.2, 11.6. (ESI-MS m/z) mass calculated 302.41 for C_21_H_33_N_3_O_8_S, found 303.00.

**Boc-Phe*ψ*(CH_2_N)Gly-OEt (3d)**

Synthesis is analogous to that of **3b**, starting from **2d** (560 mg, 1.07 mmoL), potassium carbonate (398 mg, 2.88 mmoL), and thiophenol (0.400 mL, 3.91 mmoL). After workup and column purification, 300 mg (0.89 mmol, 83% yield) of **3d **(20 : 1 EtOAc : EtOH, rf = 0.15) was obtained. ^1^H-NMR (CDCl_3_, 300 MHz): *δ* = 1.20 (3H, t, OCH_2_CH_3_, J = 7.1 Hz); 1.49 (9H, s, C(CH_3_)_3_); 2.65 (2H, m, CHCH_2_NH); 2.85 (2H, m, CHCH_2_Ph); 3.40 (2H, AB, NCH_2_CO, J = 17.4 Hz); 3.80 (1H, m, NCHCH_2_); 4.10–4.30 (2H, q, OCH_2_CH_3_, J = 7.2 Hz); 4.60 (1H, br, Boc NH); 7.10–7.40 (5H, m, CH_2_PhH2–6). Minor impurities were found in the ^1^H-NMR: H_2_O (1.60). ^13^C NMR (CDCl_3_, 75 MHz): *δ* = 172.4, 155.6, 138.0, 129.4, 128.4, 126.4, 77.2, 60.8, 51.6, 51.0, 39.1, 28.4, 14.2. (ESI-MS m/z) mass calculated 336.43 for C_18_H_28_N_2_O_4_, found 337.07.

**Boc-Val*ψ*[CH_2_N(ThyAc)]Gly-OEt (4b)**

Thymin-1-ylacetic acid (154 mg, 0.84 mmoL), N, N′, Dicyclohexylcarbodiimide (DCC) (176 mg, 0.85 mmoL), and 3-hydroxy-1,2,3-benzotriazin-4(3H)-one (DhbtOH) (137 mg, 0.84 mmoL) were dissolved in 10 mL dry N,N-Dimethylformamide (DMF). The mixture was stirred under nitrogen at room temperature for 1 hr. Then, **3b** (200.0 mg, 0.69 mmoL), which was dissolved in dry DMF (2 × 2.5 mL), was added dropwise into the mixture. The reaction mixture was heated at 50°C for 24 hrs. After TLC verification, the mixture was gravity-filtered and washed with DMF. The solvent was removed under reduced pressure. The crude mixture was dissolved in 100 mL EtOAc and then washed with 100 mL of saturated NaHCO_3_ solution. The organic layer was set aside, and the NaHCO_3_ layer was washed with EtOAc (2 × 100 mL). The organic layers were recombined and washed with 10% KHSO_4_ (3 × 50 mL), followed by saturated NaHCO_3_ (3 × 50 mL) and then brine (1 × 100 mL). The organic layer was dried over anhydrous sodium sulfate and the solvent was removed under reduced pressure. The product was purified by column chromatography (EtOAc, rf = 0.45) to give 100 mg (0.22 mmoL, 32% yield) of the desired product. ^1^H-NMR (DMSO, 300 MHz): *δ* = 0.90–1.00 (6H, m, CH(CH_3_)_2_); 1.20 (3H, t, OCH_2_CH_3_, J = 7.1 Hz); 1.49 (9H, s, C(CH_3_)_3_); 1.70 (1H, m, CH(CH_3_)_2_); 1.80 (3H, s, Thymine CH_3_); 2.90–3.60 (2H, m, CHCH_2_NCO)*; 3.45–3.60 (1H, m, NCHCH_2_)*; 3.70 and 3.90 (2H, m, NCH_2_CO)*; 4.14–4.22 (2H, q, OCH_2_CH_3_, J = 7.2 Hz); 4.40 and 4.70 (2H, 2AB, NCOCH_2_, J = 7.6 and 16.6 Hz)*; 6.60 and 6.80 (1H, 2d, Boc-NH, J = 9.4 and 9.7 Hz)*; 7.20 (1H, s, Thymine H); 11.20 (1H, 2s, Thymine-NH)*. Minor impurities were found in the ^1^H-NMR: EtOAc (1.20, 2.00, 4.00), H_2_O (3.30), and DhbtOH byproduct (5.50). (ESI-MS m/z) mass calculated 454.52 for C_21_H_34_N_4_O_7_, found 477.20 (454.52 + Na^+^). (*1 to 2 mixed rotameric species in solution.)

**Boc-Ile*ψ*[CH_2_N(ThyAc)]Gly-OEt (4c)**

Synthesis is analogous to that of **4b**, starting with thymin-1-ylacetic acid (223 mg, 1.21 mmoL), DCC (251 mg, 1.22 mmoL), and DhbtOH (196 mg, 1.20 mmoL) dissolved in 10 mL dry DMF. After stirring for 1 hr at room temperature, **3c** (300 mg, 0.99 mmol) was dissolved in dry DMF (2 × 2.5 mL) and added dropwise. The mixture was heated at 50°C for 48 hrs. After workup and purification by column chromatography (20 : 1 EtOAc : EtOH, rf = 0.45), 300 mg (0.64 mmoL, 65% yield) of the desired product was obtained. ^1^H-NMR (DMSO, 300 MHz): *δ* = 0.80–1.00 (6H, m, CHCH_3_, CHCH_2_CH_3_); 1.10 (2H, m, CHCH_2_CH_3_); 1.20 (3H, t, OCH_2_CH_3_, J = 7.1 Hz); 1.49 (9H, s, C(CH_3_)_3_); 1.60 (1H, m, CHCH_2_CH_3_); 1.80 (3H, s, Thymine CH_3_); 2.90–3.70 (2H, m, CHCH_2_NCO)*; 3.95 and 4.00 (1H, 2m, NCHCH_2_)*; 4.05–4.12 (2H, m, NCH_2_CO)*; 4.14–4.22 (2H, q, OCH_2_CH_3_, J = 7.2 Hz); 4.40 and 4.70 (2H, 2AB, NCOCH_2_, J = 7.6 and 16.6 Hz)*; 6.65 and 6.82 (1H, 2d, Boc-NH, J = 9.4 and 9.7 Hz)*; 7.20 (1H, s, Thymine H); 11.20 (1H, 2s, Thymine-NH)*. Minor impurities were found in the ^1^H-NMR: EtOAc (1.20, 2.00, 4.00), DMF (2.70 and 2.90), H_2_O (3.30), and DhbtOH byproduct (5.50). (ESI-MS m/z) mass calculated 468.54 for C_22_H_36_N_4_O_7_, found 491.13 (468.54 + Na^+^). (*1 to 2 rotameric species in solution.)

**Boc-Phe*ψ*[CH_2_N(ThyAc)]Gly-OEt (4d)**

Synthesis is analogous to that of **4b**, starting with thymin-1-ylacetic acid (198 mg, 1.08 mmoL), DCC (228 mg, 1.11 mmoL), and DhbtOH (186 mg, 1.14 mmoL) dissolved in 10 mL dry DMF. After stirring for 1 hr at room temperature, a solution of **3d** (300.0 mg, 0.60 mmoL) in dry DMF (2 × 2.5 mL) was added dropwise. The mixture was heated at 50°C for 24 hrs. After workup and purification by column chromatography (20 : 1 EtOAc : EtOH rf = 0.55), 280 mg (0.56 mmoL, 93% yield) of **4d **was obtained. ^1^H-NMR (DMSO, 300 MHz): *δ* = 1.20 (3H, t, OCH_2_CH_3_, J = 7.1 Hz); 1.49 (9H, s, C(CH_3_)_3_); 1.80 (3H, s, Thymine CH_3_); 2.60–2.85 (2H, m, CHCH_2_Ph)*; 3.05–3.50 (2H, m, CHCH_2_NCO)*; 3.80 and 3.95 (1H, 2m, NCHCH_2_)*; 4.00–4.15 (2H, m, NCH_2_CO)*; 4.20 (2H, q, OCH_2_CH_3_, J = 7.2 Hz); 4.50 and 4.70 (2H, 2AB, NCOCH_2_ J = 16.1 and 16.6 Hz)*; 6.75 and 6.90 (1H, 2d, Boc-NH, J = 8.7 and 9.4 Hz)*; 7.10–7.30 (6H, m, CH_2_PhH2–6 and Thymine H)); 11.20 (1H, 2s, Thymine-NH)*. Minor impurities were found in the ^1^H-NMR: EtOAc (1.20, 2.00, 4.00), EtOH (1.10 and 3.60), H_2_O (3.30), and DhbtOH byproduct (5.50). (ESI-MS m/z) mass calculated 502.56 for C_25_H_34_N_4_O_7_, found 530.47 (502.56 + Na^+^). (*1 to 2 rotameric species in solution.)

**Boc-Val*ψ*[CH_2_N(ThyAc)]Gly-OH (5b)**

**4b** (100 mg, 0.22 mmoL) was dissolved in tetrahydrofuran (THF) (3 mL). The solution was stirred and cooled to 0°C in an ice bath and followed by dropwise addition of 2 M NaOH (3 mL). After confirming by TLC that the reaction has gone to completion, water (25 mL) was added to quench the reaction. The mixture was extracted with EtOAc (2 × 100 mL). The aqueous layer was then acidified with 1 M HCl (aq) solution to pH 3. The solution was then extracted with EtOAc (3 × 100 mL) and then dried over anhydrous sodium sulfate. The solvent was then removed under reduced pressure. Ether was added to the solution, resulting in precipitation of the monomer. The white precipitate was filtered off and purified by column chromatography (8 : 2 DCM : MeOH, rf = 0.40) to give 51 mg (0.12 mmoL, 54% yield) of white crystalline product. m.p: 210–213°C (decomposition). ^1^H-NMR (DMSO, 500 MHz): *δ* = 0.90–1.00 (6H, m, CH(CH_3_)_2_); 1.49 (9H, s, C(CH_3_)_3_); 1.70 (1H, m, CH(CH_3_)_2_); 1.80 (3H, s, Thymine CH_3_); 3.00, 3.20, 3.45, 3.55 (2H, 4m, CHCH_2_NCO)*; 3.50 and 3.60 (1H, 2m, NCHCH_2_)*; 3.70 and 3.85 (2H, 2AB, NCH_2_CO, J = 17.4 and 18.2 Hz)*; 4.45 and 4.70 (2H, 2AB, NCOCH_2_, J = 16.3 and 17.1 Hz)*; 6.60 and 6.90 (1H, 2d, Boc-NH, J = 9.4 and 9.7 Hz)*; 7.20 (1H, s, Thymine H); 11.20 (1H, 2s, COOH)*. ^13^C NMR (DMSO, 75 MHz): *δ* = 172.1, 168.2, 167.3, 164.9, 156.4, 156.2, 151.5, 142.6, 142.3, 108.5, 78.3, 77.9, 54.3, 54.1, 52.1, 49.5, 48.7, 48.0, 39.1, 30.6, 30.3, 28.7, 19.9, 19.0, 18.3, 12.4. (ESI-MS m/z) mass calculated 426.46 for C_19_H_30_N_4_O_7_, found 449.20 (426.46 + Na^+^). (*1 to 2 rotameric species in solution). HRMS (ESI/MS_m/z_) M_calc _ for C_19_H_30_N_4_NaO_7_ 449.20, found 449.1682.

**Boc-Ile*ψ*[CH_2_N(ThyAc)]Gly-OH (5c)**

Synthesis is analogous to that of **5b**, starting with **4c** (300 mg, 0.68 mmoL) dissolved in THF (9 mL). The solution was cooled to 0°C, and 2 M NaOH (9 mL) was added dropwise. To quench the reaction 75 mL of water was added. After extraction and precipitation, the product was purified by column chromatography (8 : 2 DCM : MeOH, rf = 0.40) to give 156 mg (0.36 mmoL, 52% yield) of **5c** as white crystals. m.p: 210–213°C (decomposition). ^1^H-NMR (DMSO, 500 MHz): *δ* = 0.80–1.00 (6H, m, CHCH_3_, CHCH_2_CH_3_); 1.20–1.40 (2H, m, CHCH_2_CH_3_); 1.49 (9H, s, C(CH_3_)_3_); 1.60 (1H, m, CHCH_2_CH_3_); 1.80 (3H, s, Thymine CH_3_); 3.00, 3.30, 3.50, 3.60 (2H, 4m, CHCH_2_NCO)*; 3.55 and 3.65 (1H, 2m, NCHCH_2_)*; 3.90 and 3.95 (2H, 2AB, NCH_2_CO, J = 16.5 and 18.7 Hz)*; 4.45 and 4.70 (2H, 2AB, NCOCH_2_, J = 16.2 and 16.7 Hz)*; 6.60 and 6.90 (1H, 2d, Boc-NH, J = 9.2 and 9.5 Hz)*; 7.20 (1H, s, Thymine H); 11.20 (1H, 2s, COOH)*. ^13^C NMR (DMSO, 75 MHz): *δ* = 171.8, 168.1, 167.5, 164.8, 156.2, 156.1, 151.5, 142.5, 142.2, 108.5, 78.3, 77.9, 53.3, 52.9, 51.7, 49.3, 48.9, 48.4, 48.0, 37.3, 28.7, 25.5, 25.2, 15.7, 12.4, 11.9, 11.5. (ESI-MS m/z) mass calculated 440.49 for C_20_H_32_N_4_O_7_, found 463.13 (440.49 + Na^+^). (*1 to 2 rotameric species in solution.) HRMS (ESI/MS_m/z_) M_calc_ for C_20_H_32_N_4_NaO_4_ 463.22, found 463.1957.

**Boc-Phe*ψ*[CH_2_N(ThyAc)]Gly-OH (5d)**

Synthesis is analogous to that of **5b**, starting with **4d** (280 mg, 0.56 mmoL) dissolved in THF (12 mL). The solution was cooled to 0°C and 2 M NaOH (12 mL) was added dropwise. To quench the reaction 75 mL of water was added. After extraction and precipitation, the product was purified by column chromatography (8 : 2 DCM : MeOH, rf = 0.50) to give 150 mg (0.32 mmoL, 56% yield) of **5d** as white crystals. m.p: 210–213°C (decomposition). ^1^H-NMR (DMSO, 500 MHz): *δ* = 1.49 (9H, s, C(CH_3_)_3_); 1.80 (3H, s, Thymine CH_3_); 2.55, 2.65, 2.75, 2.85 (2H, 4AB, CHCH_2_Ph, J = 5.4 and 10.0 some peaks are obscured by H_2_O peak)*; 3.00, 3.35, 3.40, 3.60 (2H, 4AB, CHCH_2_NCO, J~6.5 Hz)*; 3.75 and 3.95 (1H, 2m, NCHCH_2_)*; 3.90 (2H, m, NCH_2_CO)* and 4.0 (2H, AB, NCH_2_CO, J = 17.8 Hz)*; 4.45 (2H, m, NCOCH_2_)* and 4.70 (2H, AB, NCOCH_2_, J = 16.4 Hz)*; 6.80 and 6.90 (1H, 2d, Boc-NH, J = 9.0 and 9.4 Hz)*; 7.10–7.30 (6H, m, CH_2_PhH2–6 and Thymine H); 11.20 (1H, 2s, COOH)*. ^13^C NMR (DMSO, 75 MHz): *δ* = 171.1, 168.3, 167.6, 164.8, 155.7, 151.5, 142.6, 139.6, 139.2, 129.5, 128.5, 128.4, 126.5, 126.3, 108.4, 78.4, 78.0, 51.7, 50.8, 48.7, 48.1, 46.0, 38.1, 28.7, 12.4, 10.3. (ESI-MS m/z) mass calculated 474.51 for C_23_H_30_N_4_O_7_, found 497.07 (474.51 + Na^+^). (*1 to 2 rotameric species in solution.) HRMS (ESI/MS_m/z_) M_calc_ for C_23_H_30_N_4_NaO_7_ 497.20, found 497.1580.

### 2.5. Oligomer Synthesis

The oligomers were synthesized according to standard solid-phase procedures [31]. The oligomers were cleaved from the resin with TFA/TFMSA/m-cresol/thioanisole mixture (6 : 2 : 1 : 1), precipitated with ethyl ether (4x), and then air-dried. The oligomers were purified by reverse-phase HPLC and characterized by MALDI-TOF mass spectrometry.

## 3. Results and Discussion

**Scheme 1:**
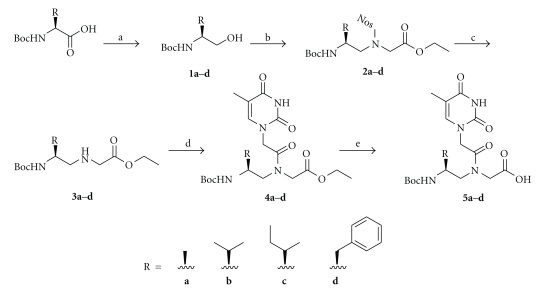
Synthesis of thymine-containing *γ*PNA monomers. Reagents and conditions: (a) *i*BuOCOCl, NMM, DME, −20°C, then NaBH_4_/H_2_O, −20°C; (b) Nos-Gly-OEt, Ph_3_P, DIAD, THF, 0°C→RT; (c) PhSH, K_2_CO_3_, CH_3_CN, RT; (d) Thy-AcOH, DCC, DhbtOH, DMF, 40°C; (e) 2 M NaOH : THF (1 : 1), 15 min (0°C), then neutralized with 1 M HCl.

The following amino acid side chains, alanine (ala), valine (val), isoleucine (ile), and phenylalanine (phe), were incorporated at the *γ*-backbone position of PNA. The corresponding thymine monomers were prepared from their respective Boc-protected L-amino acids according to the procedures outlined in Scheme 1. Mitsunobu coupling reaction was chosen over reductive amination in the preparation of the backbone intermediates because it is less prone to racemization. These chiral building blocks were then individually incorporated into the PNA oligomers using standard solid-phase synthesis procedures [31]. After cleavage from the resin, the oligomers were purified by reverse-phase HPLC (See Figures S1a–S9a, in Supplementary Material available online at doi: 10.4061/2011/652702) and characterized by MALDI-TOF mass spectrometry (Figures S1b–S9b).

**Figure 1:**
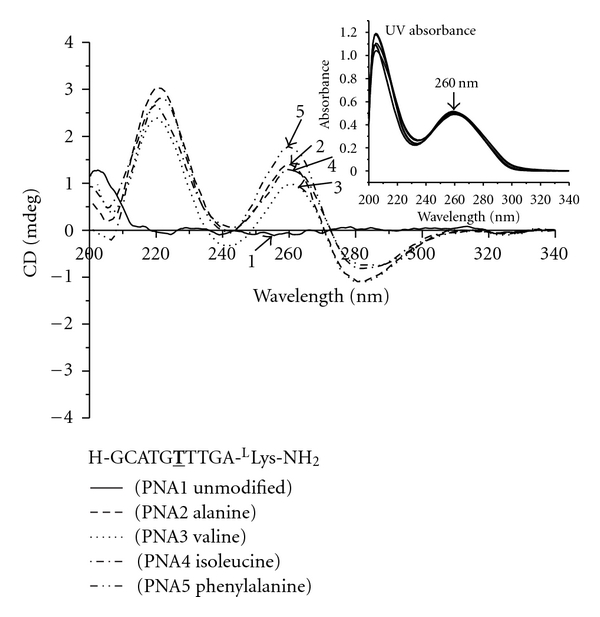
CD spectra of PNA1 through 5. Otherwise stated, all the samples for CD and UV-melting experiments were prepared in buffer containing 10 mM sodium phosphate, 0.1 mM EDTA, 100 mM NaCl at pH = 7.4. The concentration of oligomer was 5 *μ*M strand each. The CD spectra were recorded at 25°C. Inset: UV-Vis spectra of PNA1 through 5 at 90°C, demonstrating that they had the same concentration. T: modified monomer with the indicated side-chain.

CD measurements were recorded at 5 *μ*M strand concentration each in sodium phosphate buffer (10 mM sodium phosphate, 0.1 mM EDTA and 100 mM NaCl, pH 7.4) at room temperature. Inspection of Figure 1 reveals that all PNA oligomers containing *γ*-backbone modifications (PNA2 through 5) exhibited biphasic exciton coupling patterns, characteristic of a right-handed helix [32]. No noticeable CD signals were observed in the nucleobase absorption regions (220–300 nm) for the unmodified PNA (PNA1). This is expected since unmodified, single-stranded PNA does not have a well-defined helical conformation [27]. We ruled out the possibility of PNA adopting an equimolar ratio of a right-handed and left-handed helix as suggested by MD simulations [33], based on multinuclear and multidimensional NMR analyses [27]. The similarity in the CD profiles of PNA2 through 5 suggests that variations in the amino acid side chains at the *γ*-position have little effect on the overall conformation of *γ*PNAs. The subtle differences in the 240 nm minima generally indicate variations in the helical pitch, with the positive minima characteristic of a more relaxed PNA-PNA [34] and the negative minima characteristic of a more tightly wound PNA-DNA duplex [35]. The variations in the degree of winding are likely to be the result of steric clash, with the largest phenylalanine side-chain expected to induce the greatest effect. 

**Figure 2:**
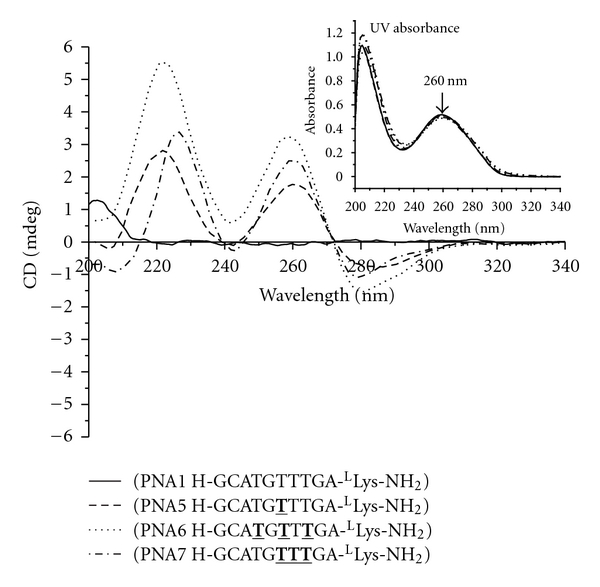
CD spectra of PNA1, 5, 6, and 7. The CD spectra were recorded at 25°C. Inset: UV-Vis spectra of PNA1, 5, 6, and 7 at 90°C. T: modified monomer with phe side-chain.

To better understand how these side chains affect the hybridization properties of *γ*PNAs, we measured their melting transitions (*T*_*m*_s) following hybridization with an antiparallel, complementary DNA strand. Our data showed that incorporation of each *γ*-building block resulted in an increase in the *T*_*m*_ of the hybrid duplex by ~4^*º*^C for all four amino acid side chains examined (Table 1), consistent with the CD data. Next, we examined the effects of backbone spacing on the conformations and hybridization properties of *γ*PNAs. We selected phenylalanine (phe) because it is the most sterically hindered side-chain among this group, which is likely to cause the greatest steric clash. PNA6 contained three phe groups placed in alternate positions with the unmodified PNA units, while PNA7 also contained three phe groups but they were placed consecutively next to one another. The two oligomers showed similar CD profiles (Figure 2) but with varied signal strengths, with PNA6 (phe 3alt) exhibiting stronger signals than PNA7 (phe 3con). It is interesting to note that PNA5, which contained one phe group, showed similar CD profile as that of PNA7, which beared three phe groups. This result indicates that one phe side-chain is sufficient to preorganize PNA into a helical motif.

UV melting data showed that PNA6 binds less tightly to a complementary DNA strand than PNA7, with the Δ*T*_*m*_ of +11 and +13°C, respectively, compared to that of the unmodified PNA-DNA. This result is unexpected because PNA6 seems more organized (or better base-stacked) based on the CD data. It should therefore be able to bind more effectively with its complementary DNA strand. The fact that it is not suggests that other factors may play a role. One possible source of extra affinity for PNA7 may come from interstrand interactions. The three phe rings placed in the consecutive arrangement might be able to interact with the adjacent DNA bases better than those placed in the alternate positions. Such interactions help anchor PNA onto the DNA strand. A second possibility may come from the fact that the phe rings are able to stack better with one another in the consecutive than in the alternate arrangement upon hybridization with the complementary DNA strand. This provides a more favorable solvophobic driving force for PNA7-DNA to remain in the duplex form rather than dissociate into individual strands. A third possibility may come from the difference in the rigidity of the two helices. Since PNA6 is more rigid than PNA7, as inferred from the CD data, it is less accommodating to the DNA strand—which may explain the lower *T*_*m*_. 

**Figure 3:**
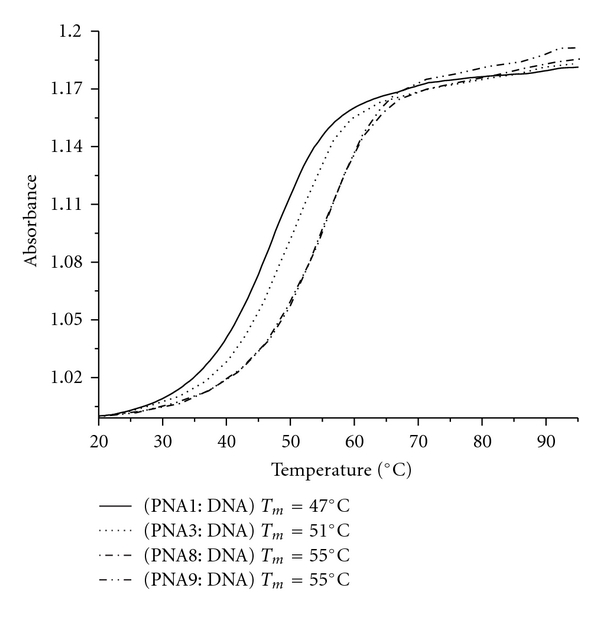
UV-melting profiles of PNA1, 3, 8, and 9 following hybridization with a complementary DNA strand. The concentration of each oligomer was 5 *μ*M. Both the heating and cooling runs were performed. They showed nearly identical profiles. T: modified monomer with phe side-chain.

**Figure 4:**
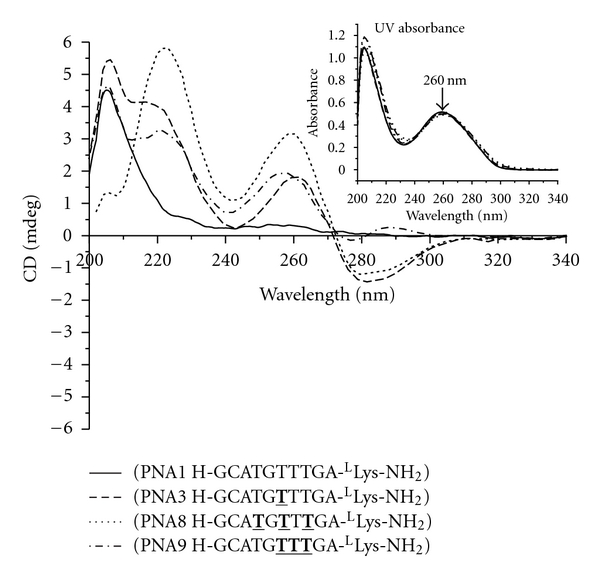
CD spectra of PNA1, 3, 8, and 9. The spectra were recorded at 25°C. Inset: UV-Vis spectra of PNA1, 3, 8, and 9 at 90°C. T: modified monomer with phe side-chain.

To delineate these three possible effects, we synthesized another set of *γ*PNA oligomers (PNA8 and PNA9) with valine side-chain at the *γ*-position. Val and phe side chains are similar in size but they differ in their ability to interact with one another. The phe side chains can *π*-stack with one another whereas the val side chains cannot. Therefore, comparing the CD and *T*_*m*_ profiles of the two sets of oligomers should provide insights into the role of *π*-*π* interaction between the phe side chains on the conformations and stability of the hybrid duplex. Our result shows that both PNA8 (val PNA alt) and PNA9 (val PNA con) have the same *T*_*m*_ (55°C) (Figure 3, Table 1) suggesting that the extra binding affinity of PNA6 (phe alt) and PNA7 (phe con) comes from *π*-*π* interaction. However, it is not clear at this point whether the phe ring stacking occurs intramolecularly with the adjacent PNA bases or intermolecularly with the DNA bases—both of which produce similar results. The same CD patterns were observed for the second set of oligomers, with the alternating PNA8 exhibiting greater CD signal than the consecutively modified PNA9 (Figure 4). It is not obvious why the alternate arrangement displayed greater helical character than their consecutive counterparts. One possible explanation is that the “helical directors,” in this case the stereogenic centers at the *γ*-backbone position, are more spread out in the alternate than in the consecutive arrangement. The alternate arrangement, therefore, should enable the “helical directors” to induce and propagate the helical sense of the oligomer more effectively than the consecutive arrangement, since each chiral director has fewer achiral units in front of them to direct. This explanation is consistent with the “Sergeants and Soldiers” concept proposed by Green and coworkers [36, 37] to explain helical induction in polymers. When comparing the two arrangements it should be noted that although they both start out at the same position (the third unit from the *C*-terminus), the alternate sequence contained *γ*-building blocks that are spread out further toward the *N*-terminus. This should give them greater advantage in organizing the remaining achiral, *N*-terminal residues since our previous study showed that helical induction occurs in a unidirectional fashion from *C*- to *N*-terminus [27]. This may explain why *γ*PNAs with the alternate placement exhibit greater CD signals, hence more helical character, than those with the consecutive arrangement. Detailed explanations for this phenomenon will await further structural studies. Nevertheless, this result shows that the chiral backbone units can be placed in the consective or alternative position with minimal effect on the oligomer's binding affinity, unless the side-chain contains an aromatic group which slightly favors the consecutive arrangement.

In addition to the perfect match, we have determined the melting transitions for the PNA-DNA hybrid duplexes containing single-base mismatched binding sites (see Table 2). Our result shows that conformationally preorganized *γ*PNAs can discriminate between related sequences, with similar degree of specificity as that of the unmodified PNA oligomer. The Δ*T*_*m*_s for mismatched duplexes range from −11 to −19°C, depending on the mismatch pair with T-T mismatch being the least discriminating. Though incorporation of additional *γ*-backbone modified units further improved the binding affinity of PNAs towards complementary DNA strands, it does not significantly affect their ability to discriminate single-base mismatched sequences. This result shows that the binding affinity of PNA can be improved by installing an appropriate stereogenic center at the *γ*-backbone position without compromising sequence specificity. 

In summary, we have shown that a number of amino acid side chains with varying degree of steric hindrance can be placed at the *γ*-position of the *N*-(2-aminoethyl) glycine backbone of PNA without inducing adverse affect on the hybridization properties of PNAs. Spectroscopic measurements showed that these PNA oligomers adopted a right-handed helix and hybridized to a complementary DNA strand with higher affinity than their unmodified counterpart, with Δ*T*_*m*_ ~ +4°C per unit incorporated. Despite their strong binding affinities, these conformationally preorganized *γ*PNAs can discriminate related sequences, with similar level of specificity as that of the unmodified PNA. Placement of the chiral *γ*-units (consecutive versus alternating) has subtle effects on the confirmations and hybridization properties of *γ*PNA oligomers depending on whether the side chains are involved in intramolecular *π*-*π* stacking; but overall, these effects are negligible. Our results confirm that PNAs are true hybrids of peptides and nucleic acids, capable of binding DNA (and RNA), and can be functionalized with a number of amino acid side chains without inducing adverse affects on the hybridization properties of the oligomers. The ability to modify the structures and chemical functionalities of oligonucleotide analogues is important because it allows other functional properties beside hybridization, such water solubility, cellular uptake, biodistribution, and pharmacokinetics to be augmented and/or further improved upon and undesired features, such nonspecific binding and toxicity, to be further minimized. The ease and flexibility of synthesis, along with superior hybridization properties and enzymatic stability, make *γ*PNAs an attractive nucleic acid platform for various biological and medical applications—as molecular tools as well as therapeutic and diagnostic reagents.

## Figures and Tables

**Table 1:** *T*_*m*_s of PNA-DNA hybrid duplexes containing perfectly matched sequence.

Name	Sequence	*T*_*m*_ (°C)	Δ*T*_*m*_ (°C)
PNA1 (Unmod)	H-GCATGTTTGA-^L^Lys-NH_2_	47	—
PNA2 (Ala)	H-GCATG**T**TTGA-^L^Lys-NH_2_	51	+4
PNA3 (Val)	H-GCATG**T**TTGA-^L^Lys-NH_2_	51	+4
PNA4 (Ile)	H-GCATG**T**TTGA-^L^Lys-NH_2_	51	+4
PNA5 (Phe)	H-GCATG**T**TTGA-^L^Lys-NH_2_	51	+4
PNA6 (Phe 3alt)	H-GCA**T**G**T**T**T**GA-^L^Lys-NH_2_	58	+11
PNA7 (Phe 3con)	H-GCATG**TTT**GA-^L^Lys-NH_2_	60	+13
PNA8 (Val 3alt)	H-GCA**T**G**T**T**T**GA-^L^Lys-NH_2_	55	+8
PNA9 (Val 3con)	H-GCATG**TTT**GA-^L^Lys-NH_2_	55	+8

**Table 2:** *T*_*m*_ *s* (°C) of PNA-DNA hybrid duplexes containing perfectly matched and single-base mismatched sequences.

Oligomer	*X* = *A*	*T* (Δ*T*_*m*_)	G(Δ*T*_*m*_)	C(Δ*T*_*m*_)
PNA1 (Unmod.)	47	35 (−12)	32 (−15)	31 (−16)
PNA2 (Ala)	51	40 (−11)	34 (−17)	33 (−18)
PNA3 (Val)	51	40 (−11)	34 (−17)	33 (−18)
PNA4 (Ile)	51	39 (−12)	34 (−17)	33 (−18)
PNA5 (Phe)	51	40 (−11)	35 (−16)	35 (−16)
PNA6 (Phe 3alt)	58	45 (−13)	42 (−16)	39 (−19)
PNA7 (Phe 3con)	60	47 (−13)	41 (−19)	41 (−19)
PNA8 (Val 3alt)	55	41 (−14)	37 (−18)	38 (−17)
PNA9 (Val 3con)	55	42 (−13)	37 (−18)	39 (−16)
