# Supplementary material for: Effect of Steric Constraint at the γ-Backbone Position on the Conformations and Hybridization Properties of PNAs
Source: J Nucleic Acids. 2011 May 25;2011:652702. doi: 10.4061/2011/652702 (PMC3138043; doi:10.4061/2011/652702)

Effect of Steric Constraint at the γ-Backbone Position on the Conformations and Hybridization Properties of PNAs

Matthew J. Crawford, Srinivas Rapireddy, Raman Bahal, Iulia Sacui, and Danith H. Ly*

Department of Chemistry and Center for Nucleic Acids Science and Technology (CNAST), Carnegie Mellon University, 4400 Fifth Avenue, Pittsburgh, PA 15213

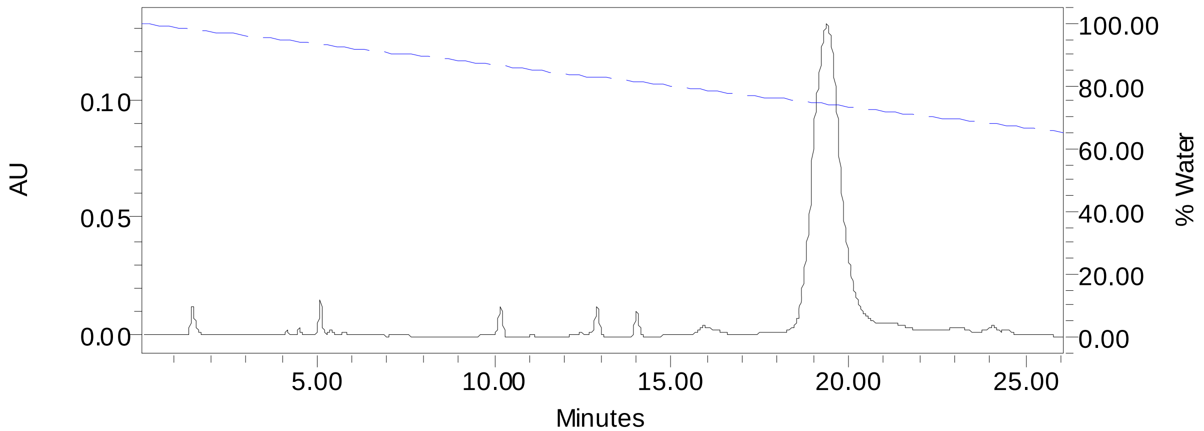

Figure S1. HPLC trace of the crude PNA7 oligomer. Eluent A: 0.1% TFA in water and eluent B: 0.1% TFA in ACN. The gradient was 0-40% of eluent B in 40 minutes at 45°C with a flow rate of 3.0 mL/min.

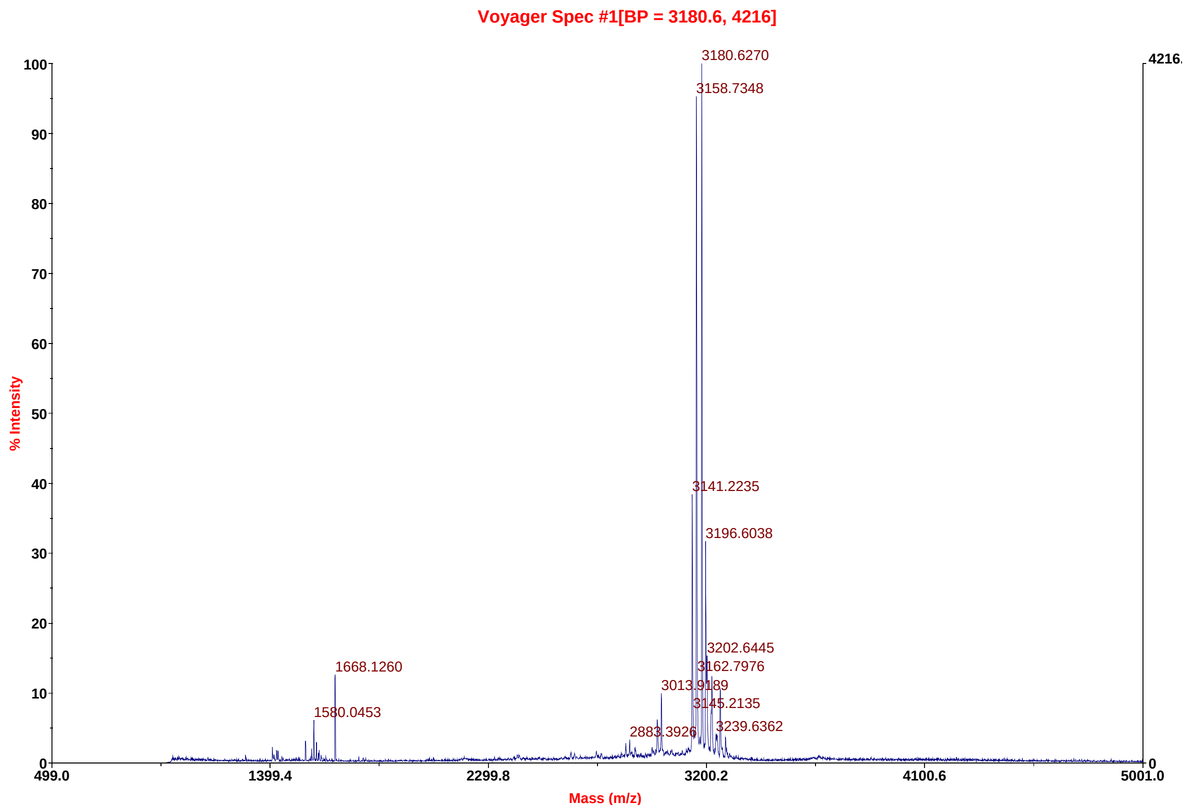

Figure S2. MALDI-TOF profile of PNA7 oligomer. Mass calculated: 3157.0, observed 3158.73, 3180.63 (3157.0 + Na^+), and 1668.13 (1/2 mass).

Oligomer Name	***Mass Calculated (m/z)***	***Experimental Mass (m/z)***
**PNA1 (Unmodified)**	2887	2885
**PNA2 (Alanine Mod)**	2901	2899
**PNA3 (Valine Mod)**	2929	2927
**PNA4 (Isoleucine Mod)**	2943	2942
**PNA5 (Phenylalanine Mod)**	2977	2976
**PNA6 (Phenylalanine 3 Alt Mod)**	3157	3158
**PNA7 (Phenylalanine 3 Con Mod)**	3157	3158
**PNA8 (Valine 3 Alt Mod)**	3011	3008
**PNA9 (Valine 3 Con Mod)**	3011	3008

Figure S3. Calculated and observed masses (MALDI-TOF MS) for the PNA and γ-Modified PNA oligomers utilized in the studies.

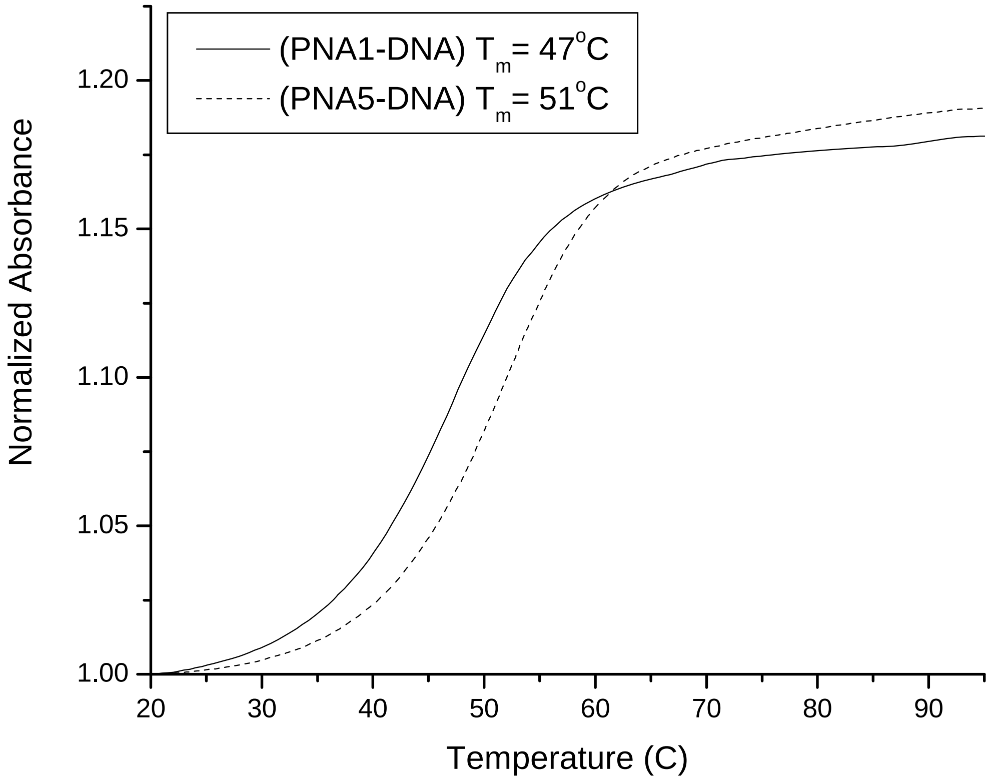

Figure S4. UV-melting curves of PNA1-DNA and PNA5-DNA duplexes containing perfectly matched sequences. The samples were prepared in buffer containing 0.1 mM EDTA, 100 mM NaCl, 10 mM sodium phosphate (pH 7.0) at 5μM duplex strand concentration each. The T_ms were determined by taking the first derivatives of the UV-melting curves.

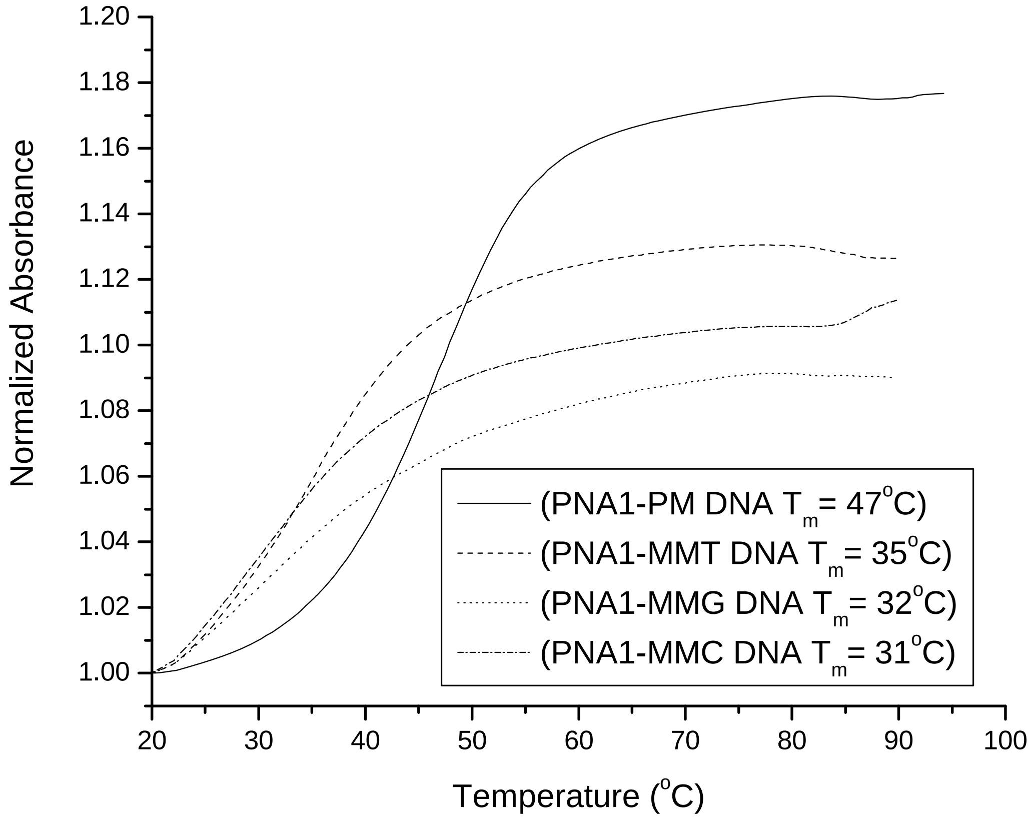

Figure S5. UV-melting curves of PNA1-DNA duplexes containing perfectly matched (PM) and mismatch (MM) sequences. The samples were prepared in buffer containing 0.1 mM EDTA, 100 mM NaCl, 10 mM sodium phosphate (pH 7.0) at 5μM duplex strand concentration each. The T_ms were determined by taking the first derivatives of the UV-melting curves.

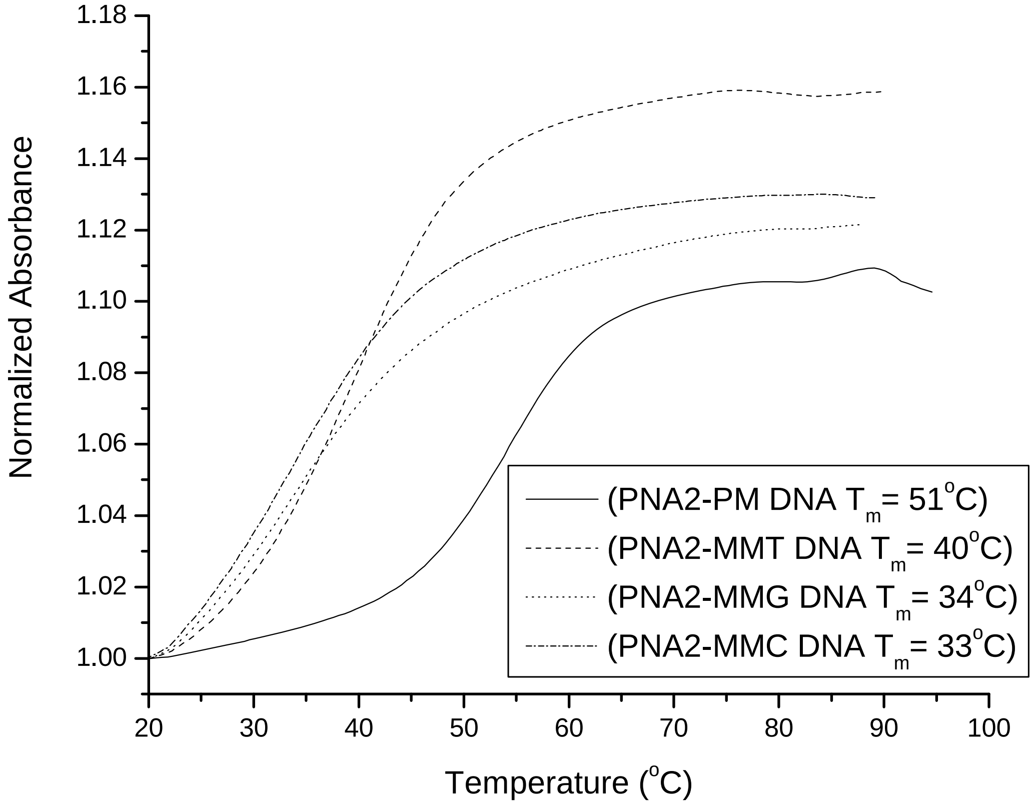

Figure S6. UV-melting curves of PNA2-DNA duplexes containing perfectly matched (PM) and mismatch (MM) sequences. The samples were prepared in buffer containing 0.1 mM EDTA, 100 mM NaCl, 10 mM sodium phosphate (pH 7.0) at 5μM duplex strand concentration each. The T_ms were determined by taking the first derivatives of the UV-melting curves.

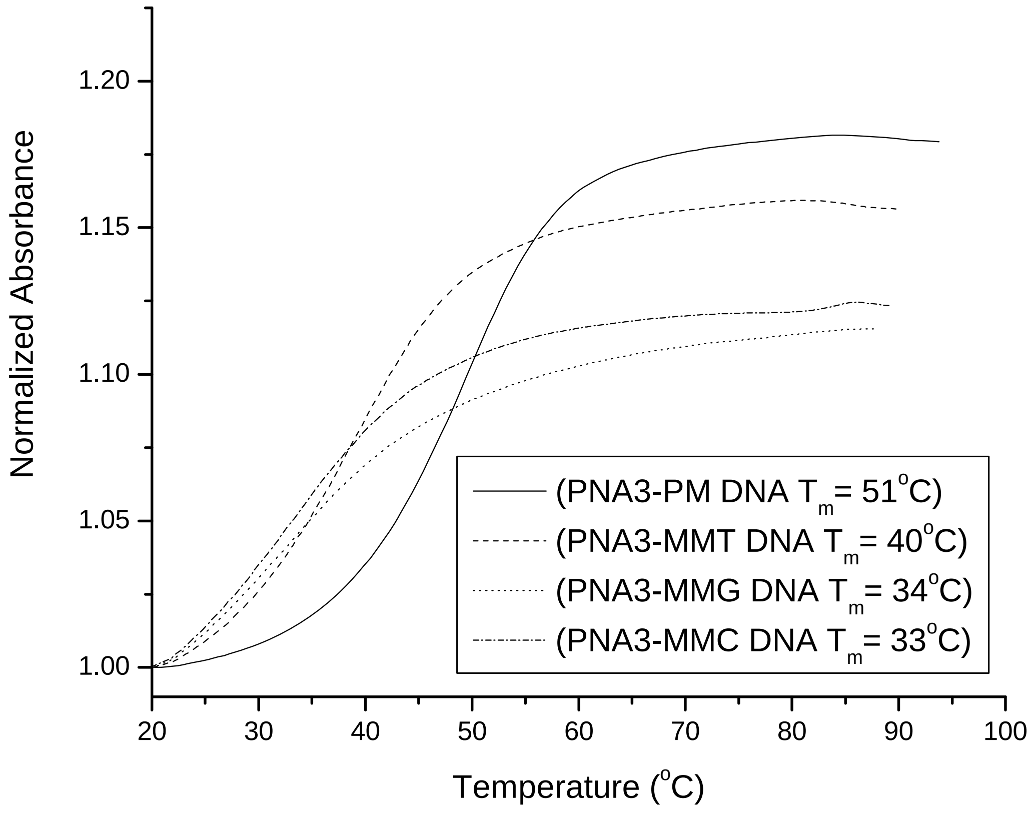

Figure S7. UV-melting curves of PNA3-DNA duplexes containing perfectly matched (PM) and mismatch (MM) sequences. The samples were prepared in buffer containing 0.1 mM EDTA, 100 mM NaCl, 10 mM sodium phosphate (pH 7.0) at 5μM duplex strand concentration each. The T_ms were determined by taking the first derivatives of the UV-melting curves.

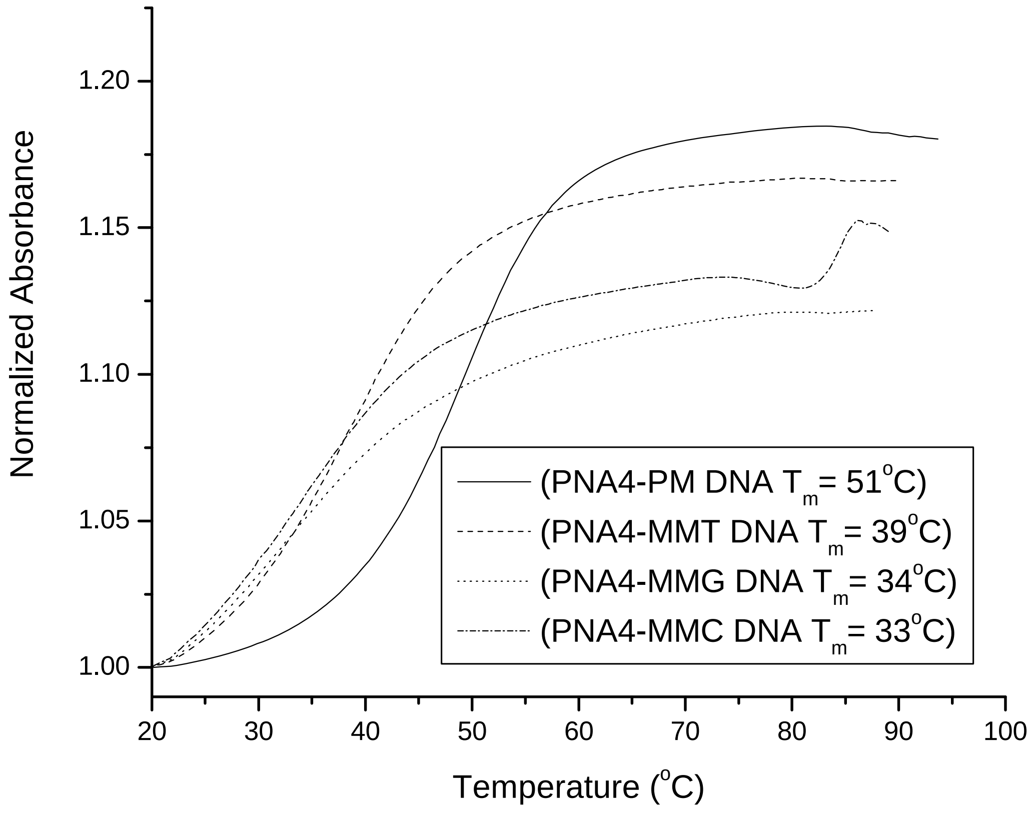

Figure S8. UV-melting curves of PNA4-DNA duplexes containing perfectly matched (PM) and mismatch (MM) sequences. The samples were prepared in buffer containing 0.1 mM EDTA, 100 mM NaCl, 10 mM sodium phosphate (pH 7.0) at 5μM duplex strand concentration each. The T_ms were determined by taking the first derivatives of the UV-melting curves.

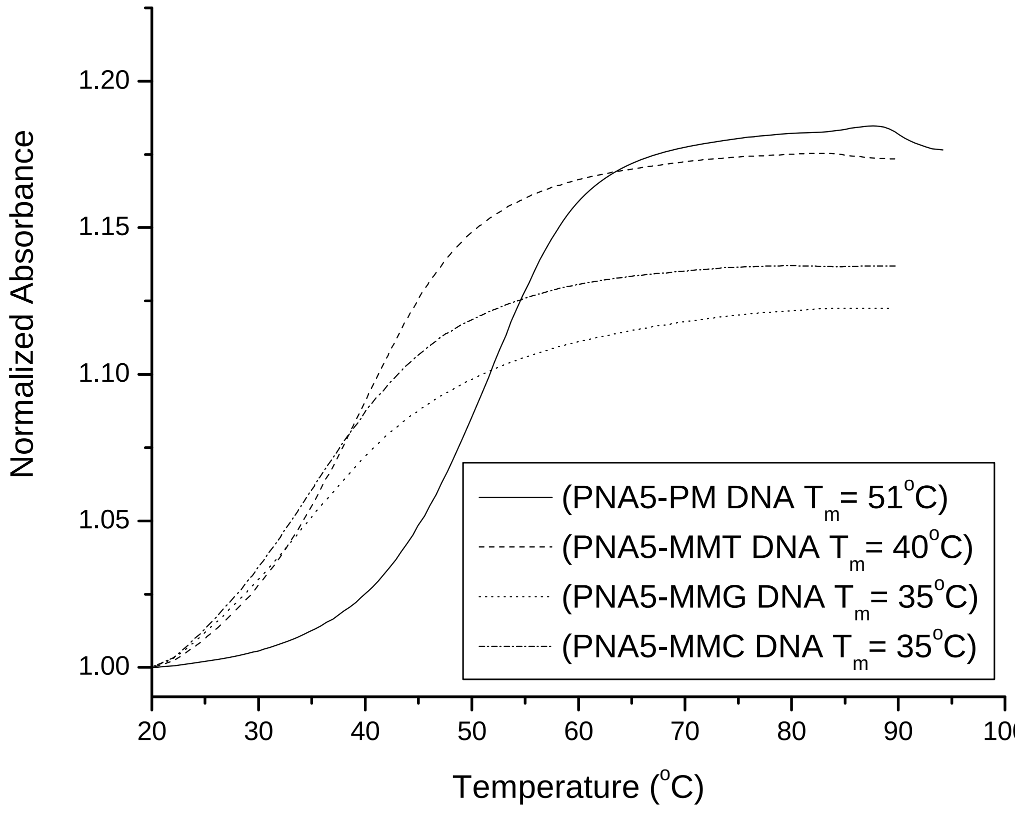

Figure S9. UV-melting curves of PNA5-DNA duplexes containing perfectly matched (PM) and mismatch (MM) sequences. The samples were prepared in buffer containing 0.1 mM EDTA, 100 mM NaCl, 10 mM sodium phosphate (pH 7.0) at 5μM duplex strand concentration each. The T_ms were determined by taking the first derivatives of the UV-melting curves.

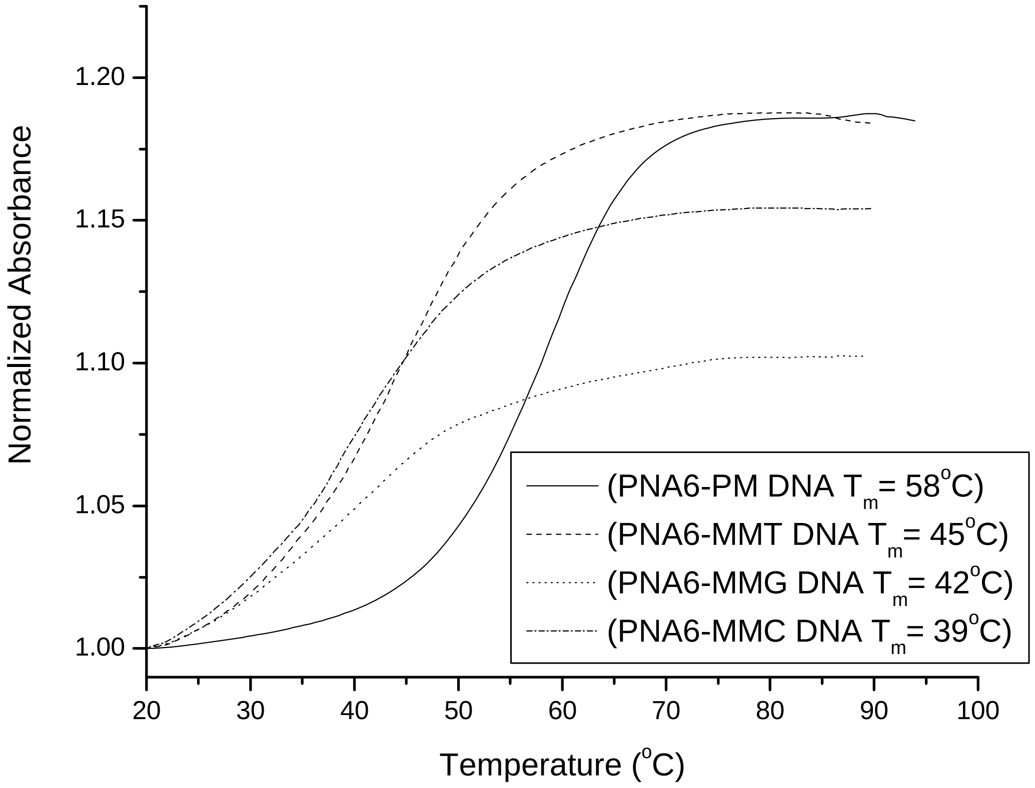

Figure S10. UV-melting curves of PNA6-DNA duplexes containing perfectly matched (PM) and mismatch (MM) sequences. The samples were prepared in buffer containing 0.1 mM EDTA, 100 mM NaCl, 10 mM sodium phosphate (pH 7.0) at 5μM duplex strand concentration each. The T_ms were determined by taking the first derivatives of the UV-melting curves.

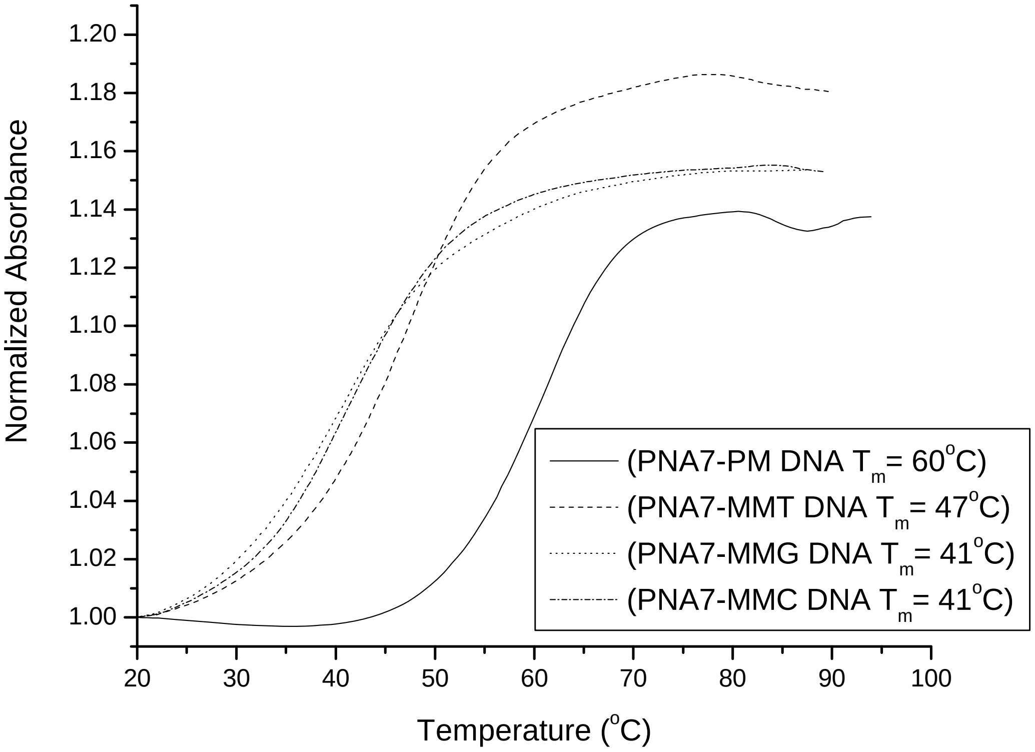

Figure S11. UV-melting curves of PNA7-DNA duplexes containing perfectly matched (PM) and mismatch (MM) sequences. The samples were prepared in buffer containing 0.1 mM EDTA, 100 mM NaCl, 10 mM sodium phosphate (pH 7.0) at 5μM duplex strand concentration each. The T_ms were determined by taking the first derivatives of the UV-melting curves.

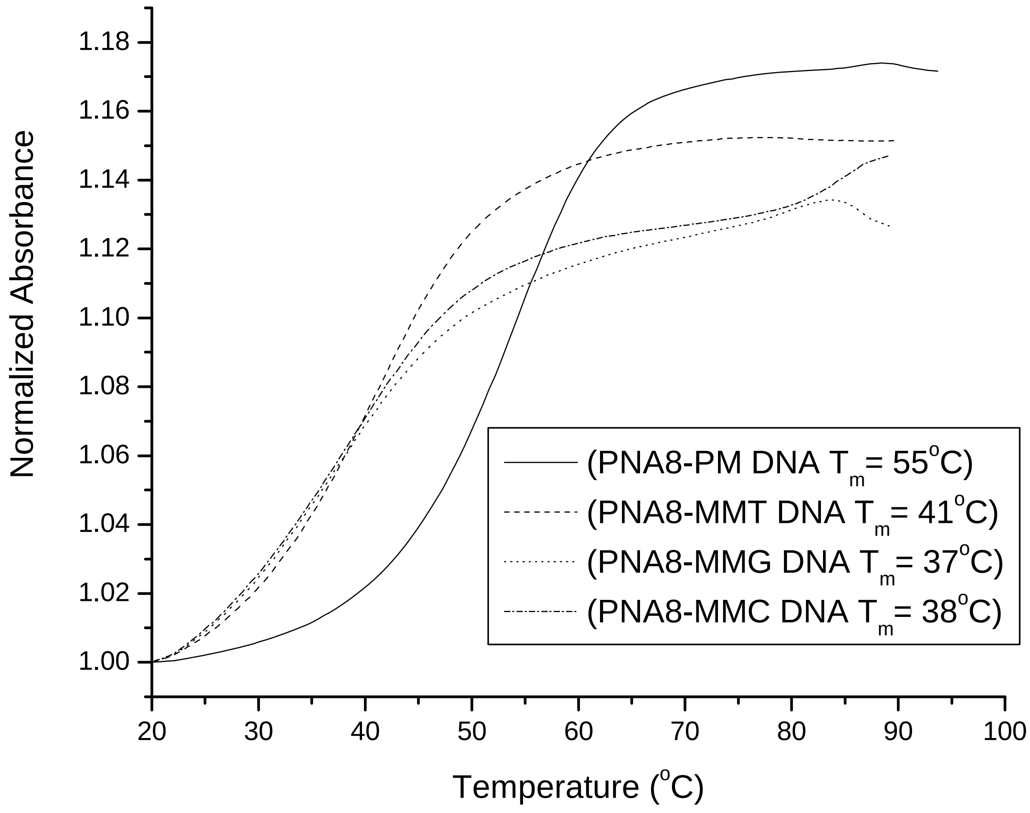

Figure S12. UV-melting curves of PNA8-DNA duplexes containing perfectly matched (PM) and mismatch (MM) sequences. The samples were prepared in buffer containing 0.1 mM EDTA, 100 mM NaCl, 10 mM sodium phosphate (pH 7.0) at 5μM duplex strand concentration each. The T_ms were determined by taking the first derivatives of the UV-melting curves.

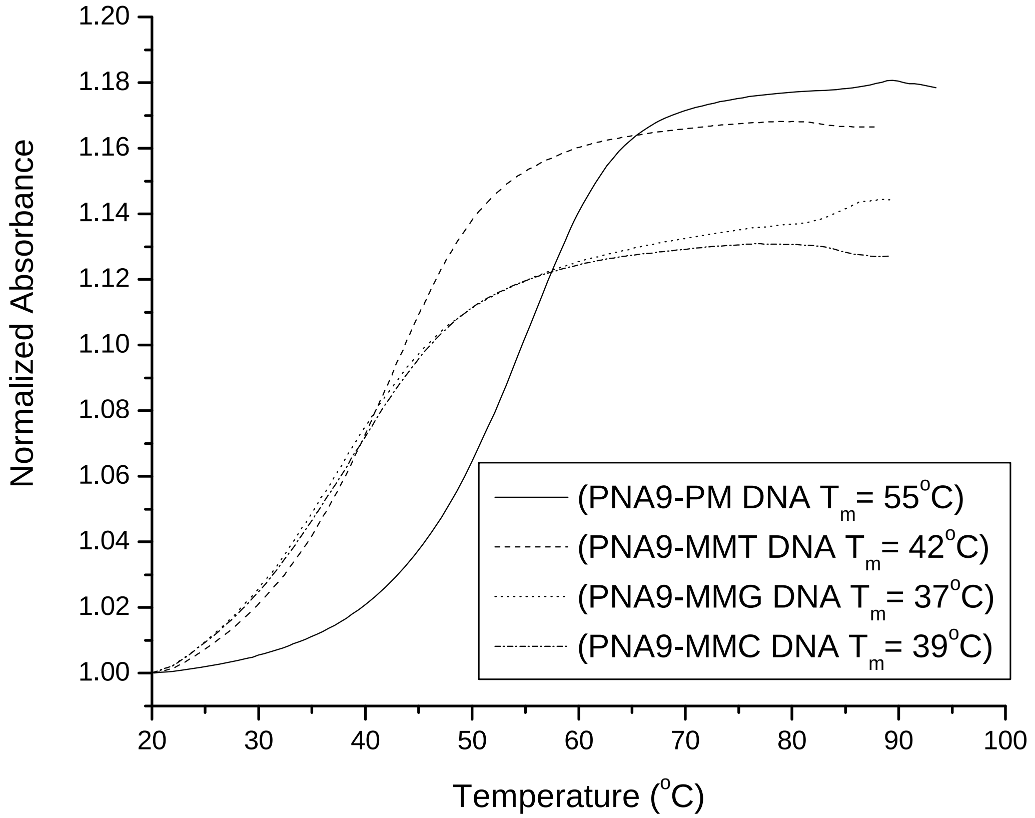

Figure S13. UV-melting curves of PNA9-DNA duplexes containing perfectly matched (PM) and mismatch (MM) sequences. The samples were prepared in buffer containing 0.1 mM EDTA, 100 mM NaCl, 10 mM sodium phosphate (pH 7.0) at 5μM duplex strand concentration each. The T_ms were determined by taking the first derivatives of the UV-melting curves.

¹HNMR

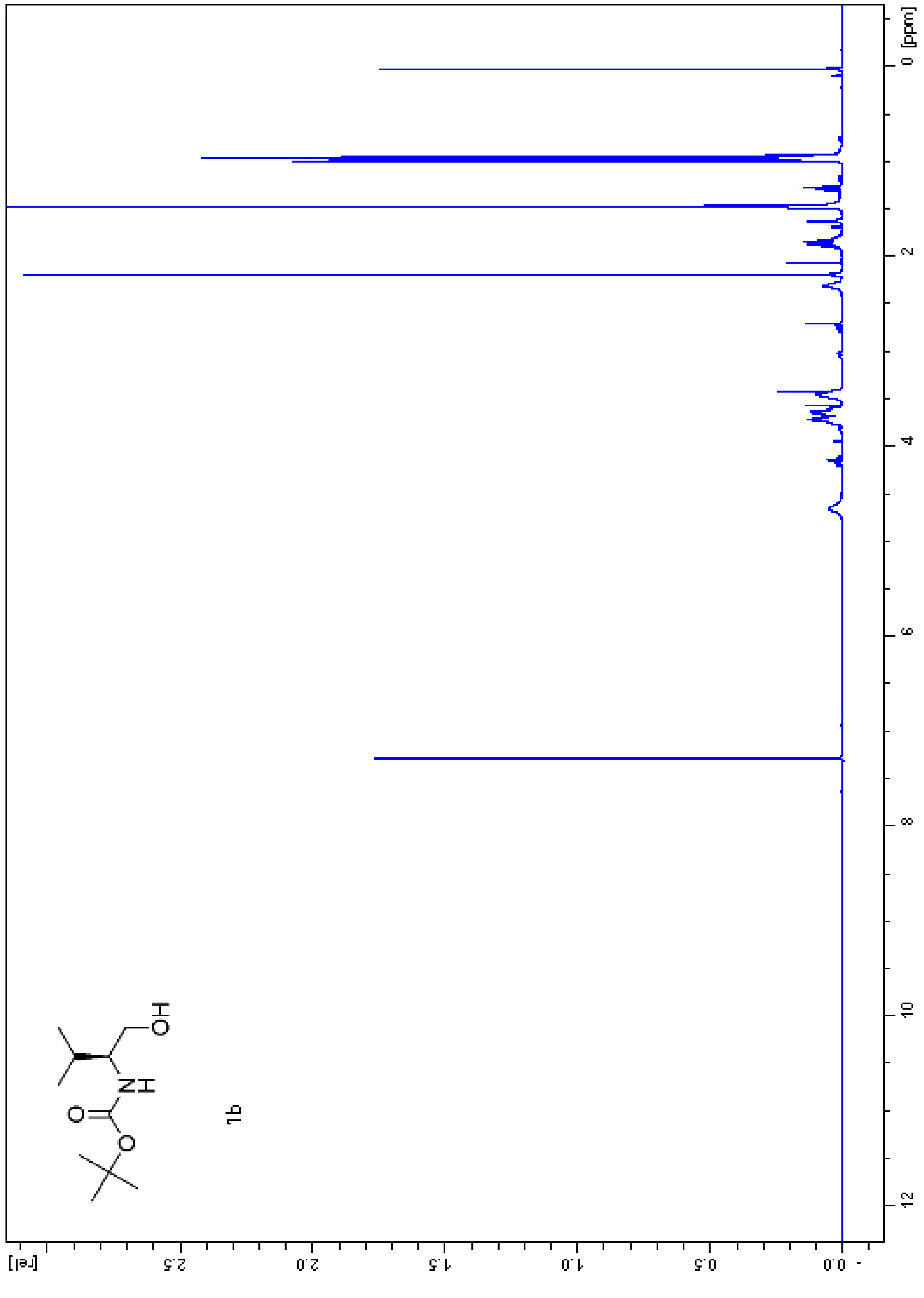

^{1}H NMR

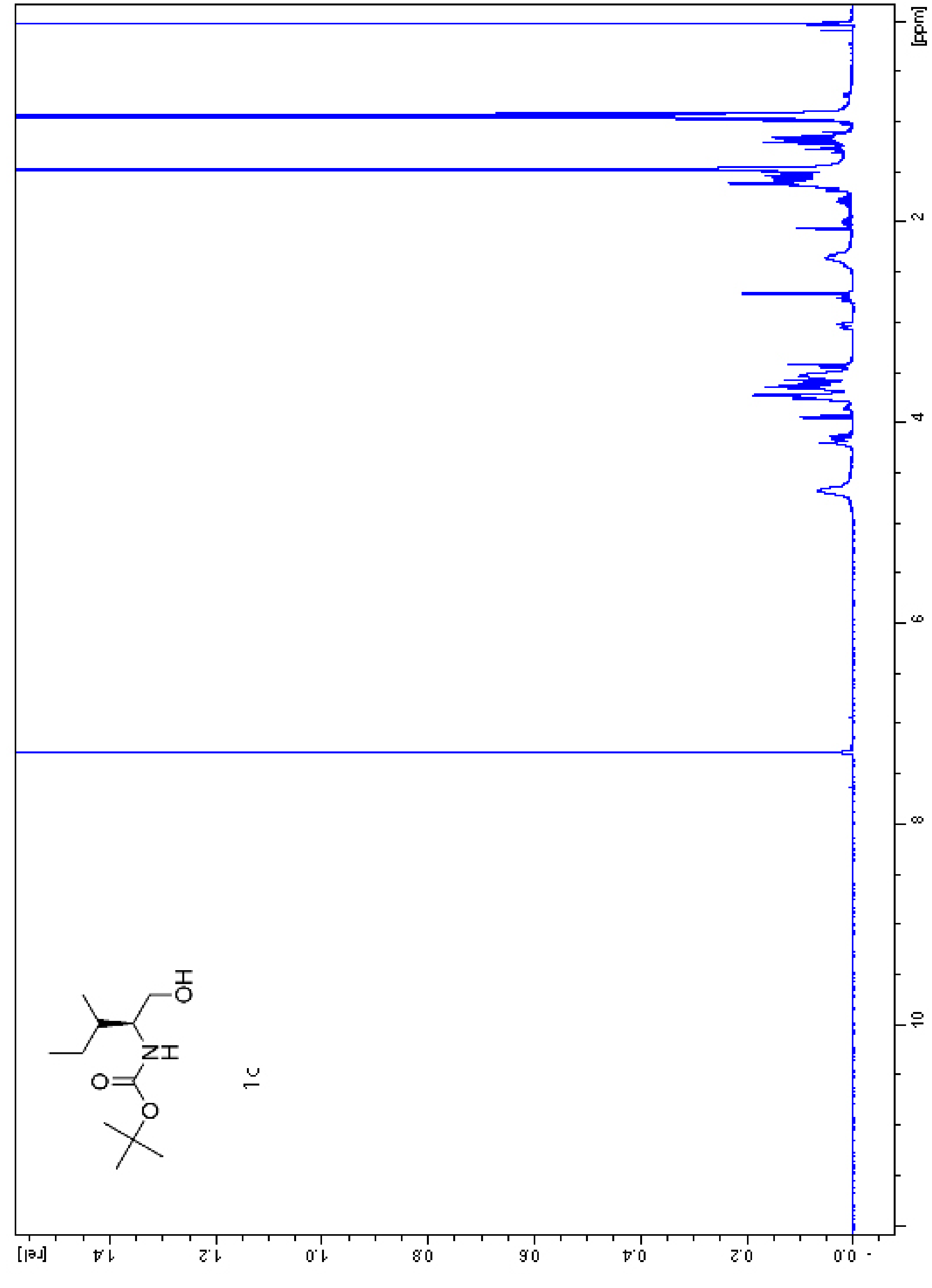

^{1}H NMR

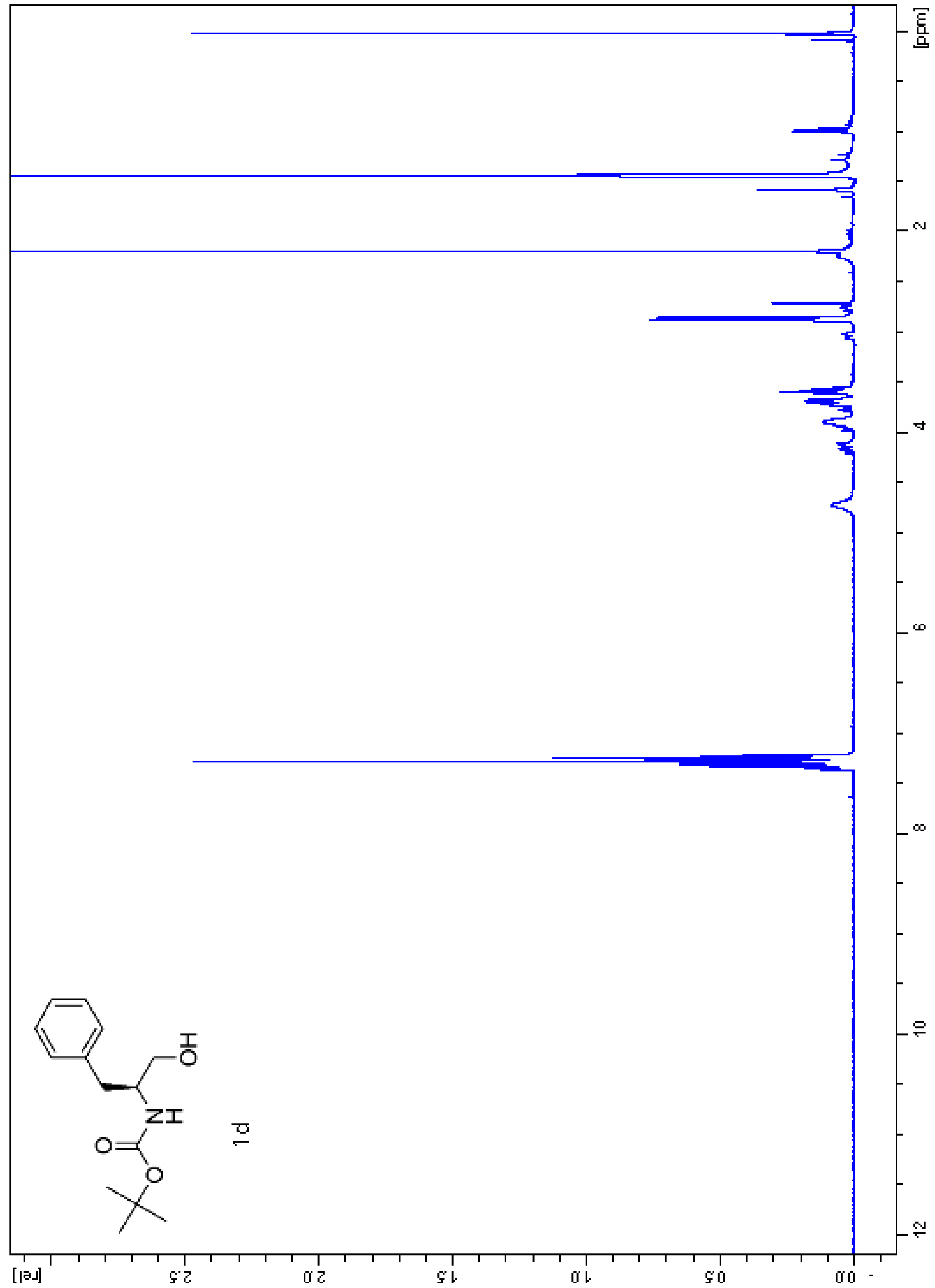

^{1}H NMR

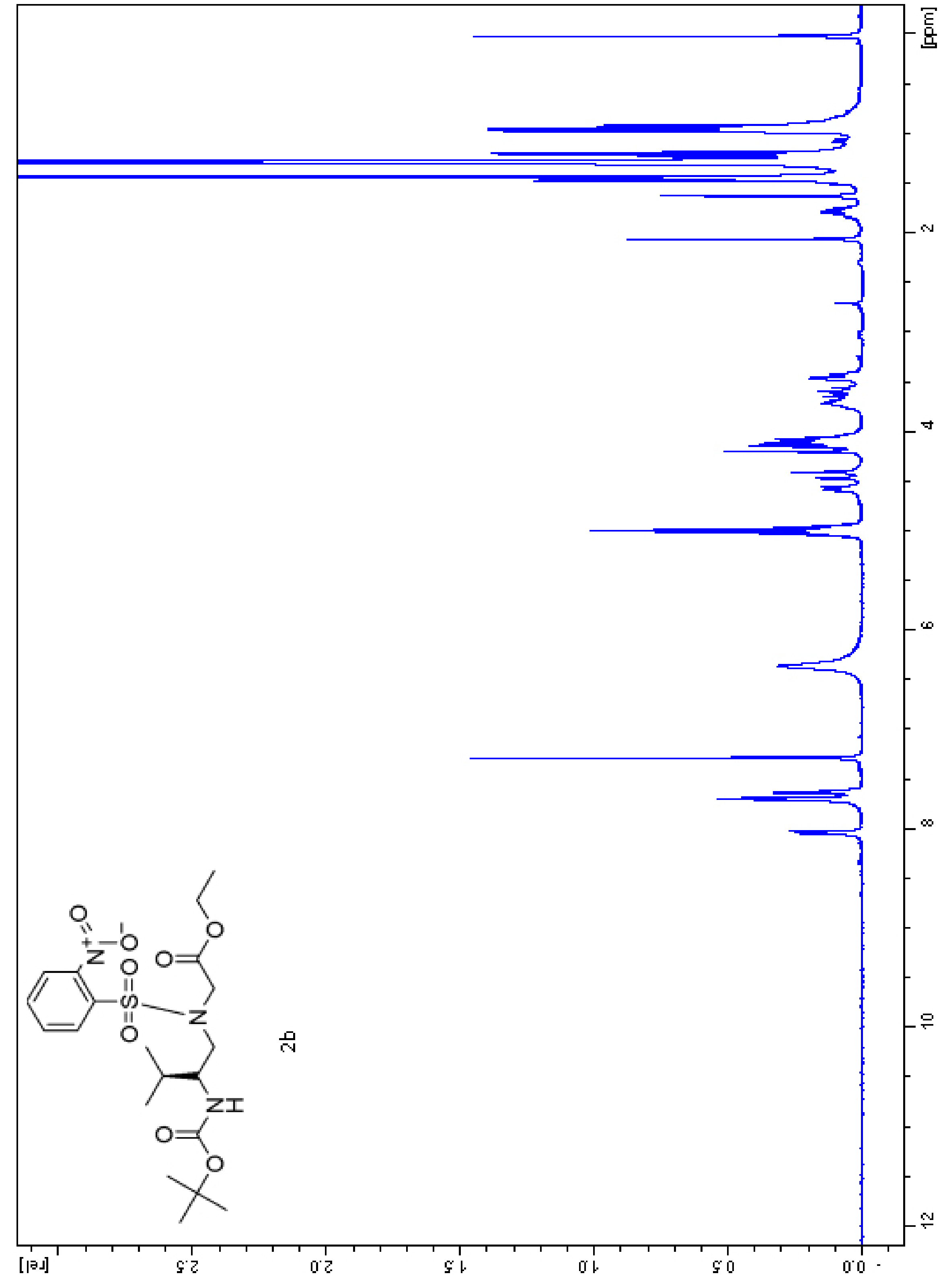

^{1}H NMR

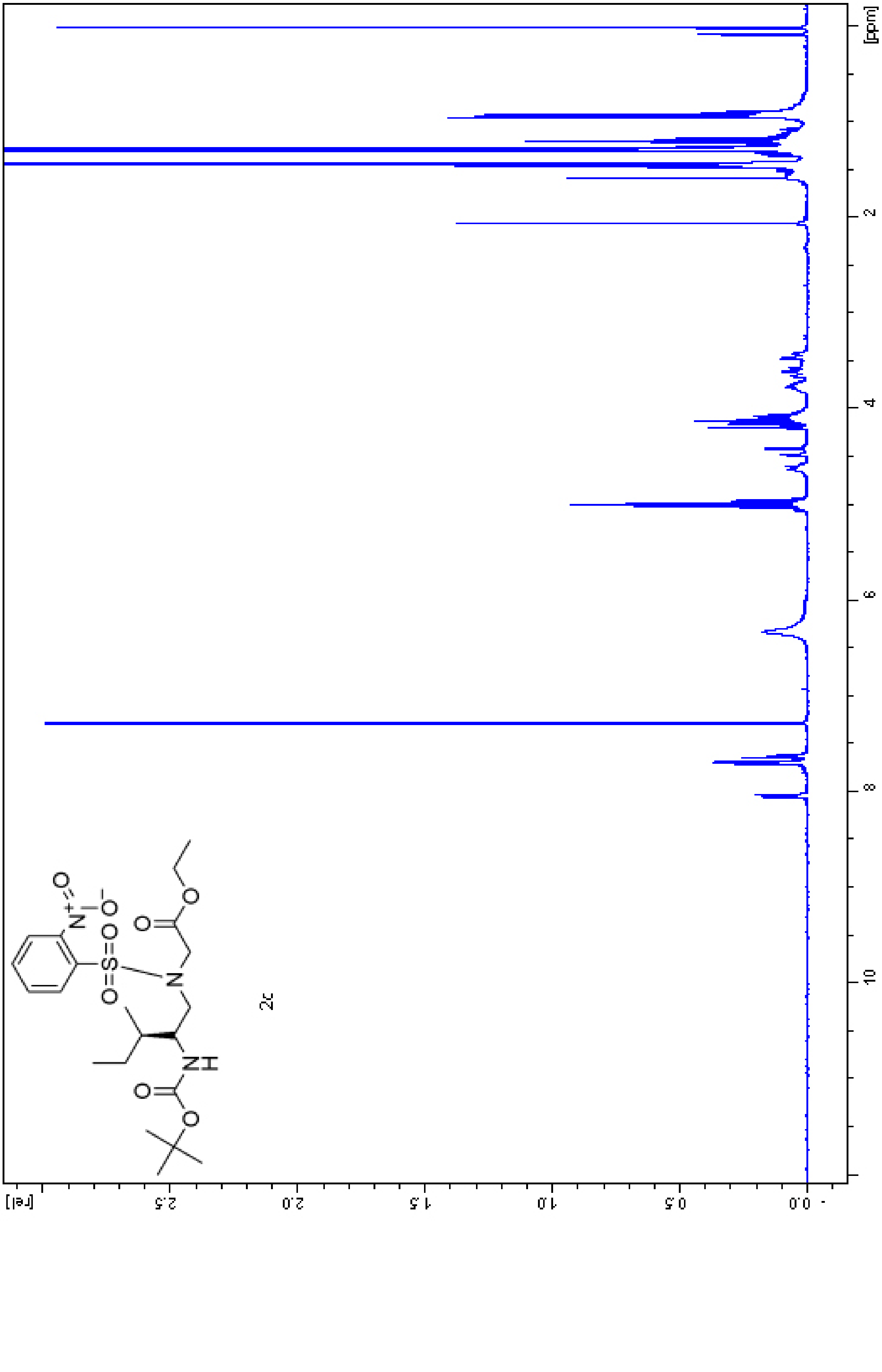

^{1}H NMR

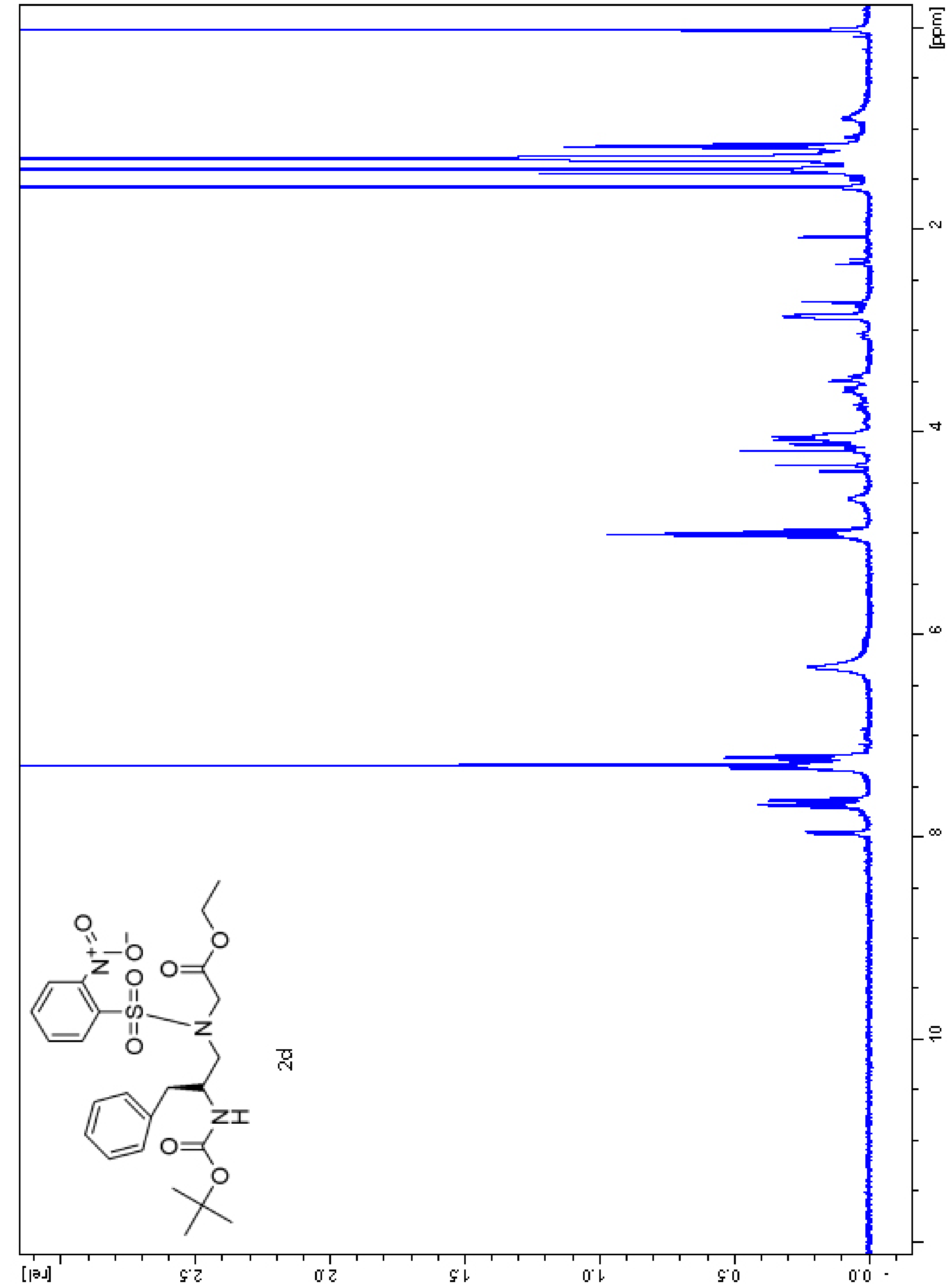

^{1}H NMR

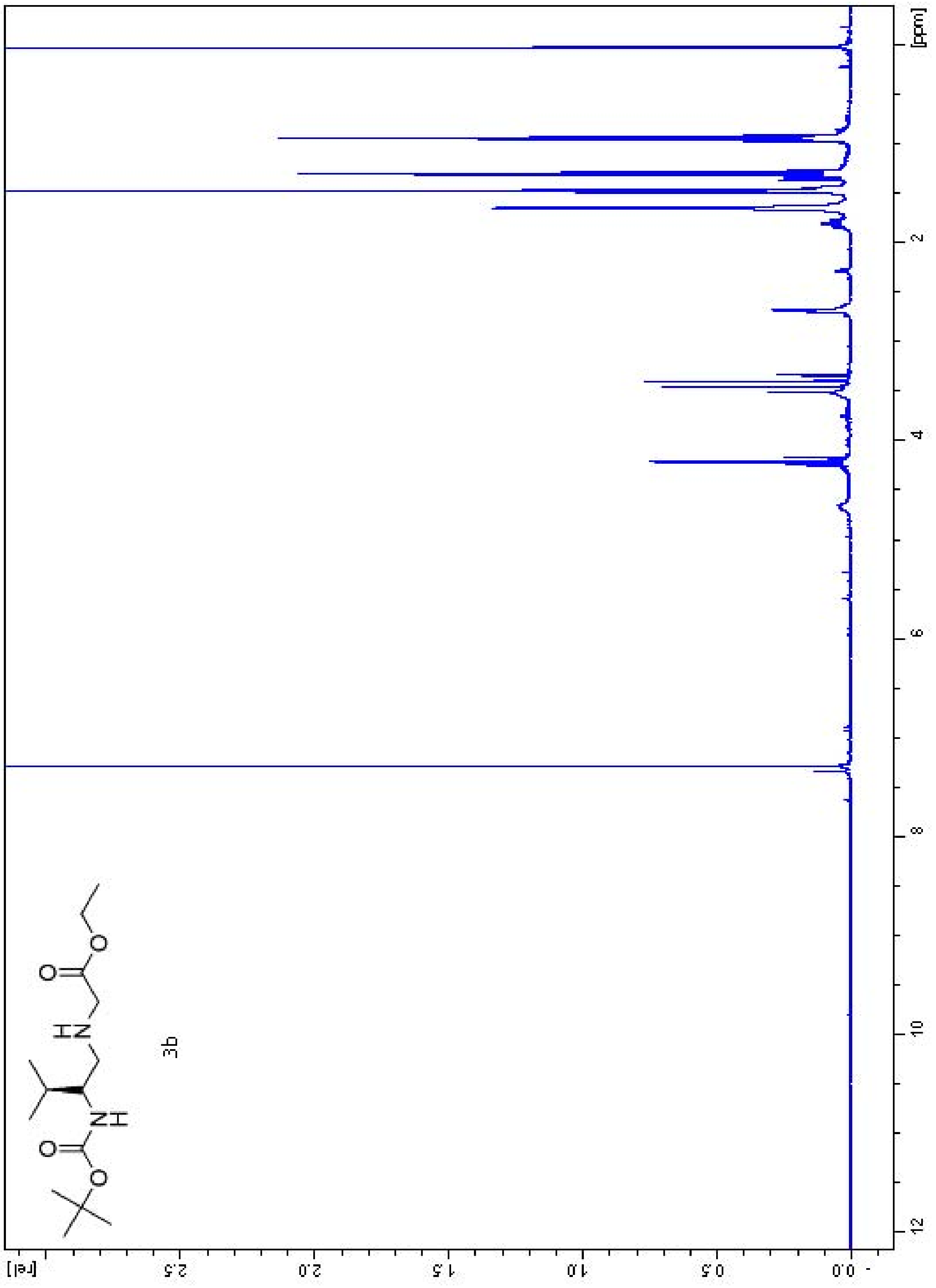

^{13}C NMR

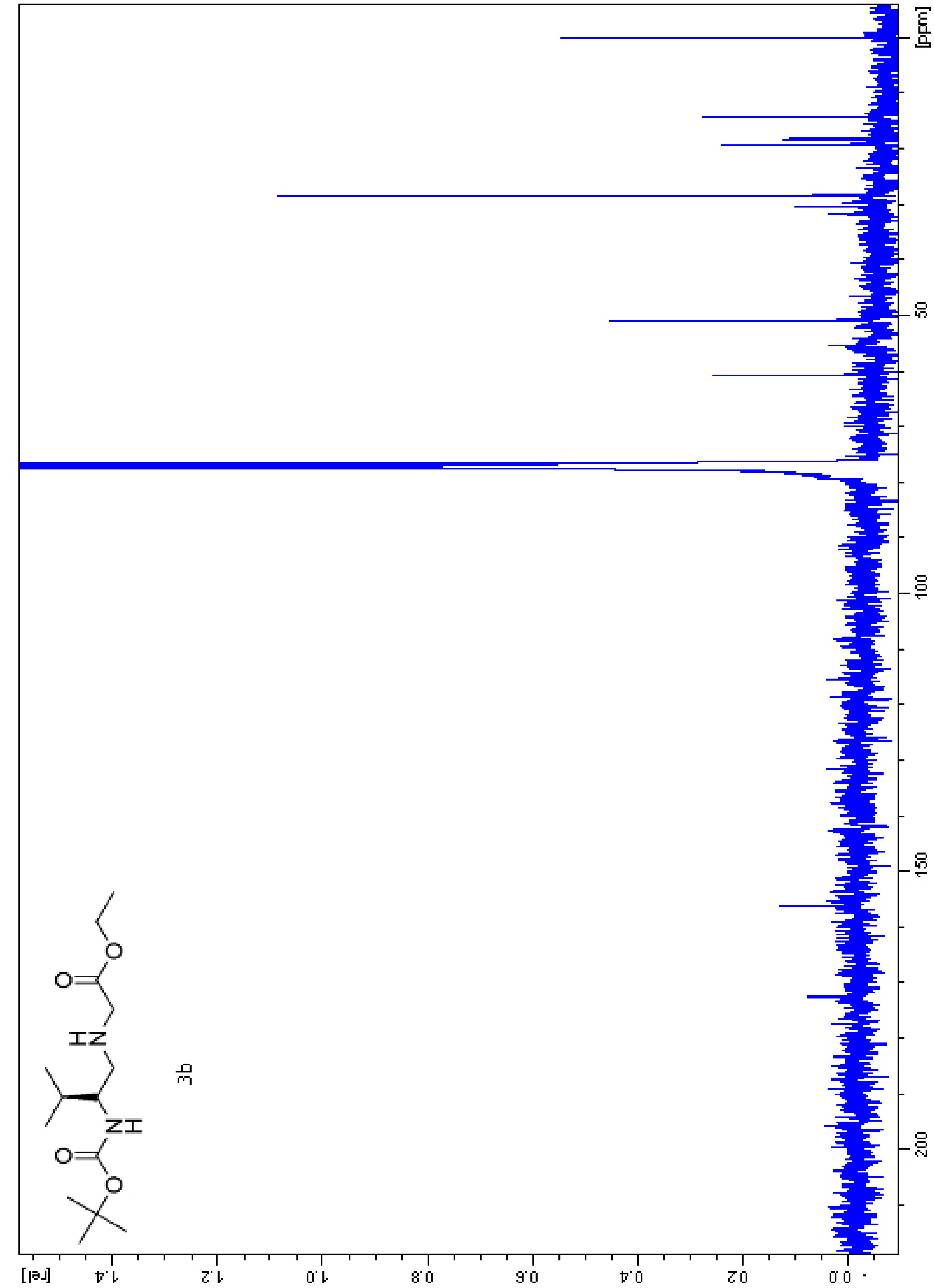

^{1}H NMR

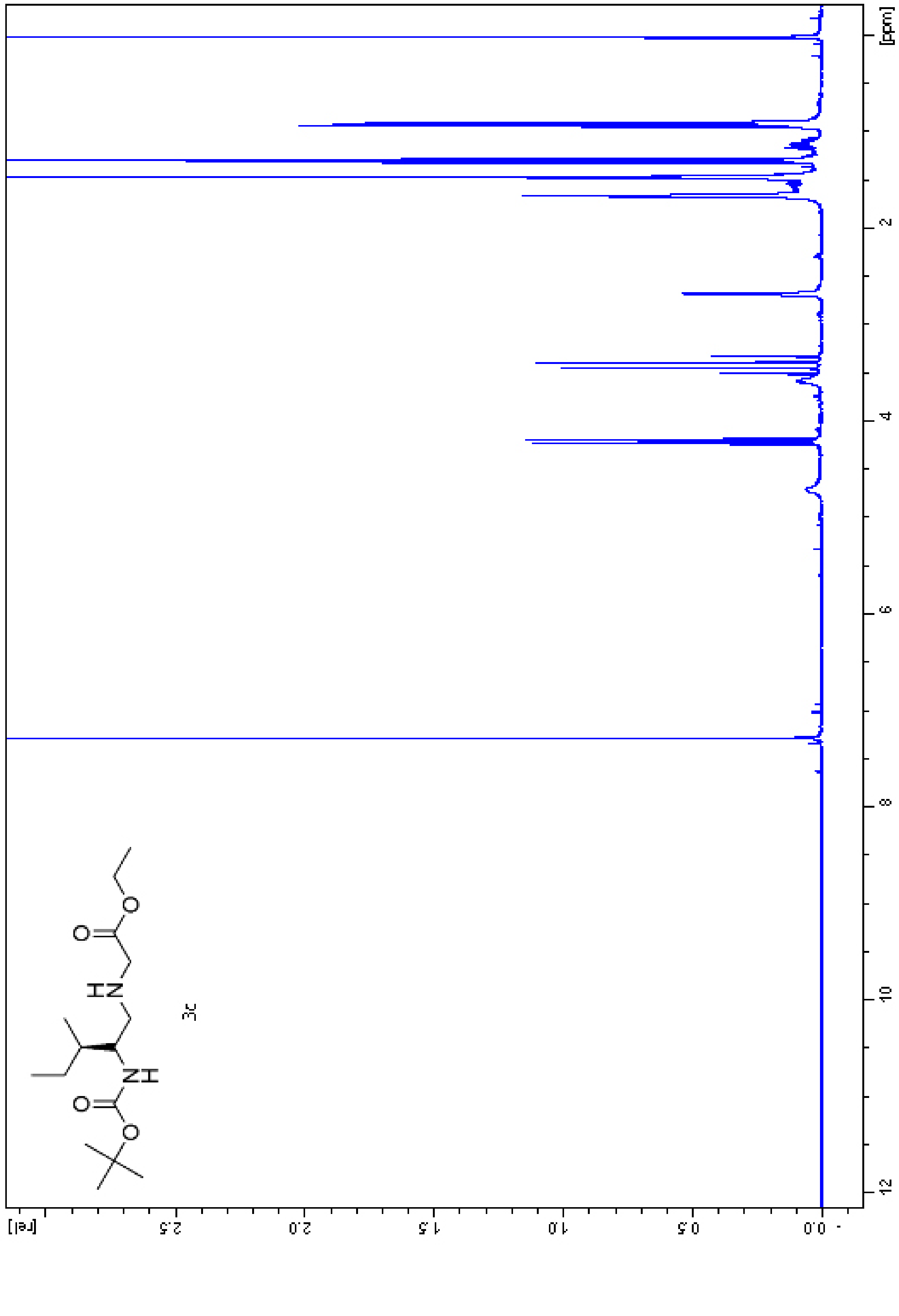

^{13}C NMR

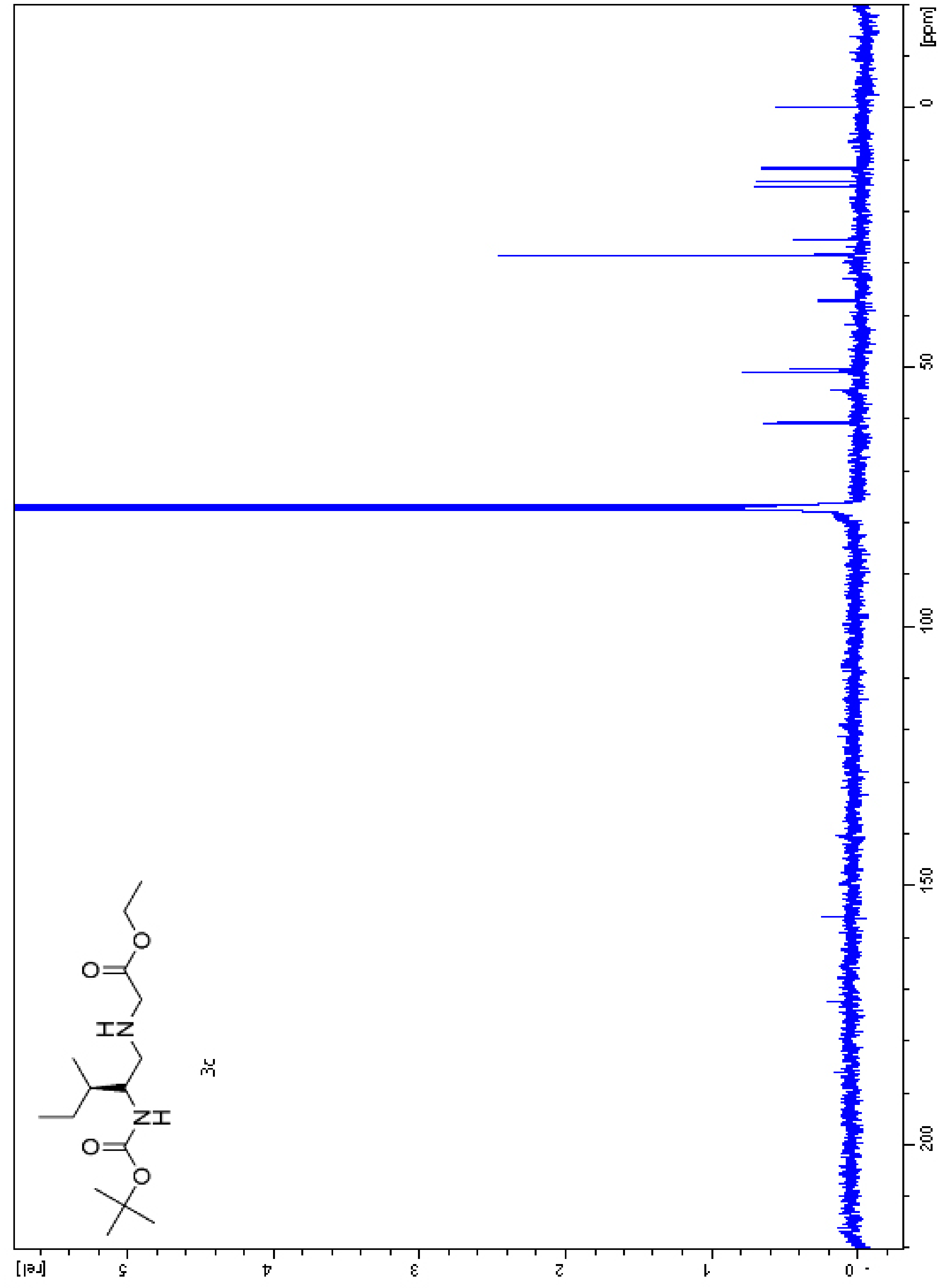

^{1}H NMR

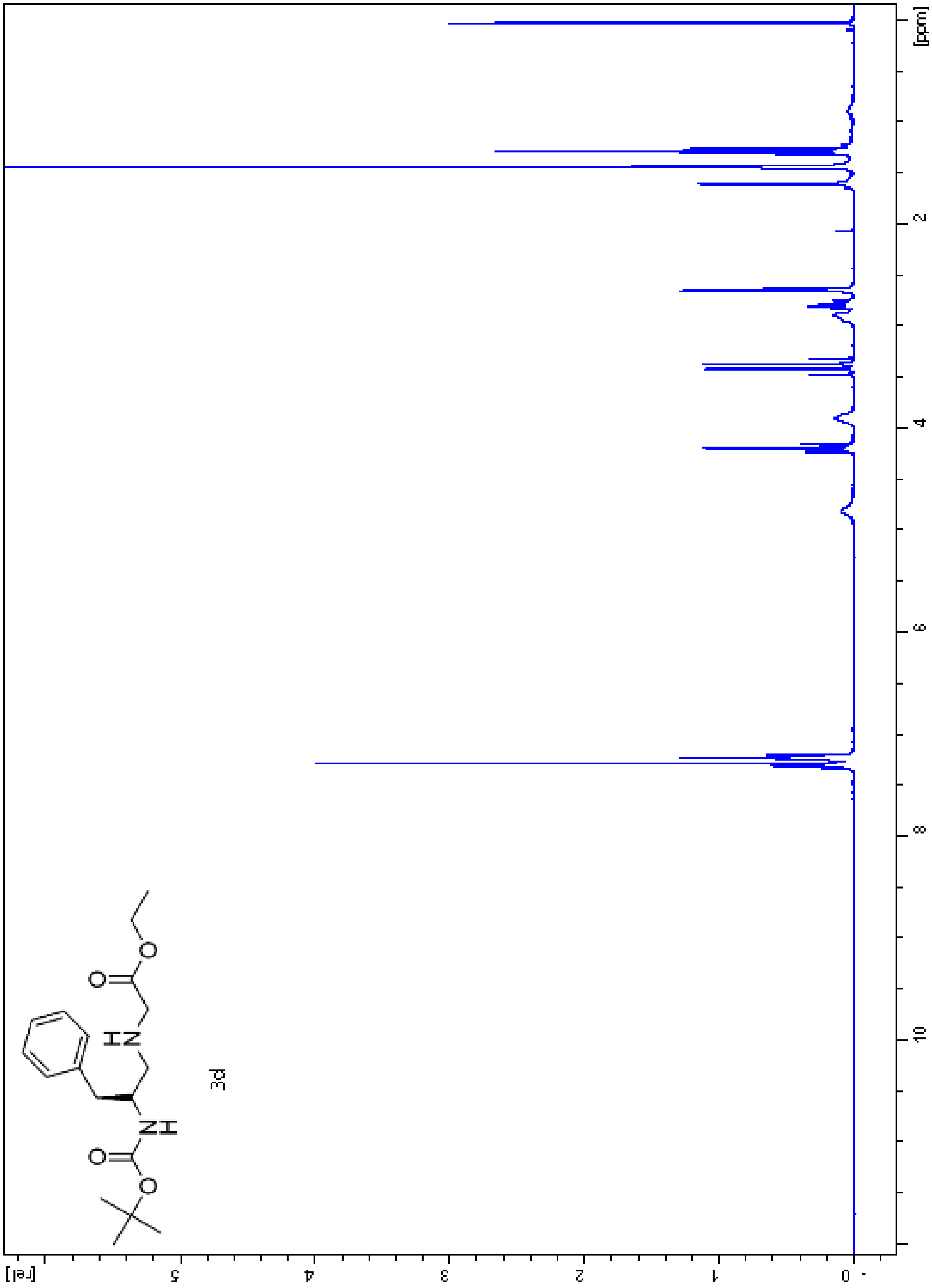

^{13}C NMR

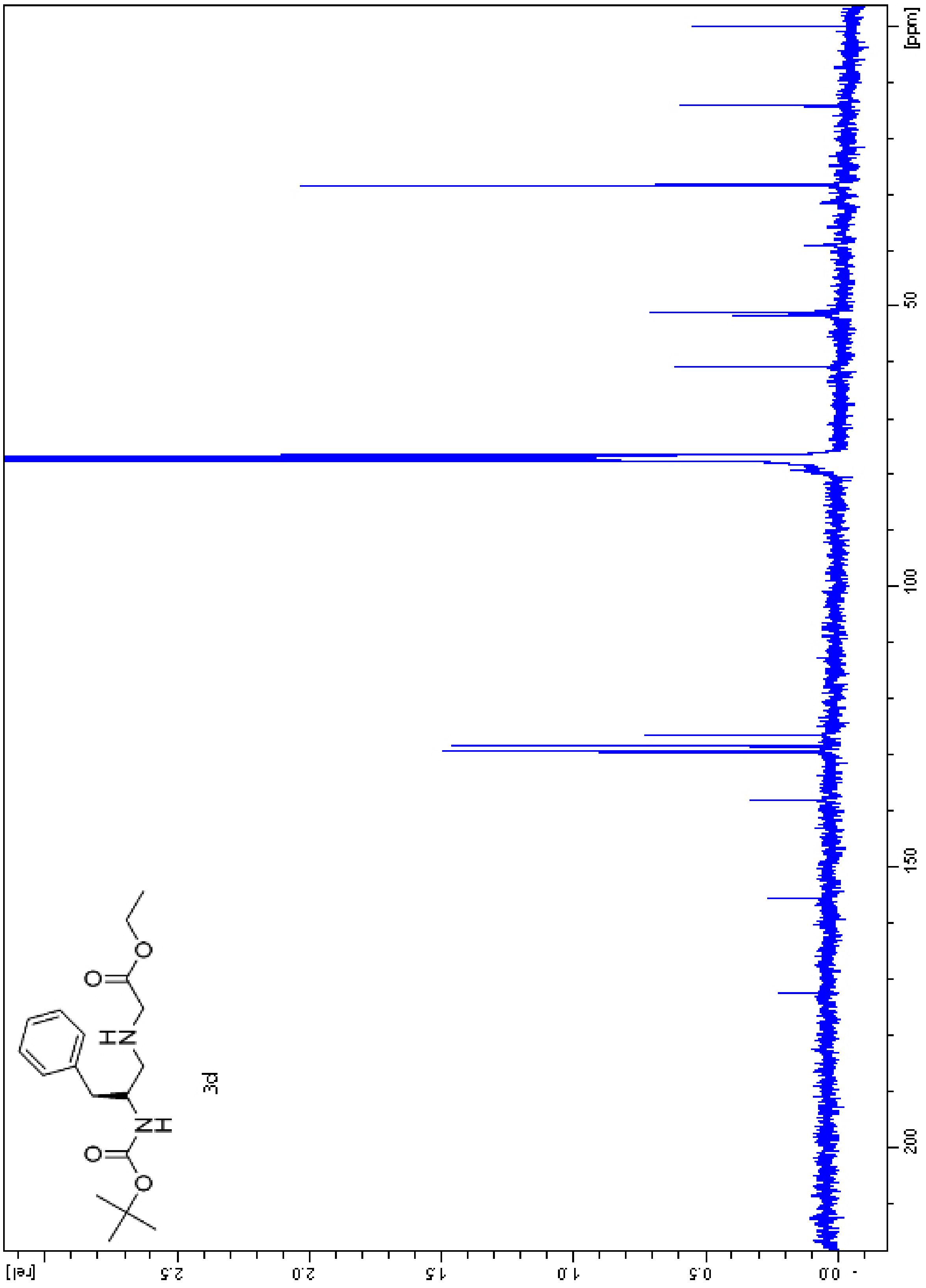

^{1}H NMR

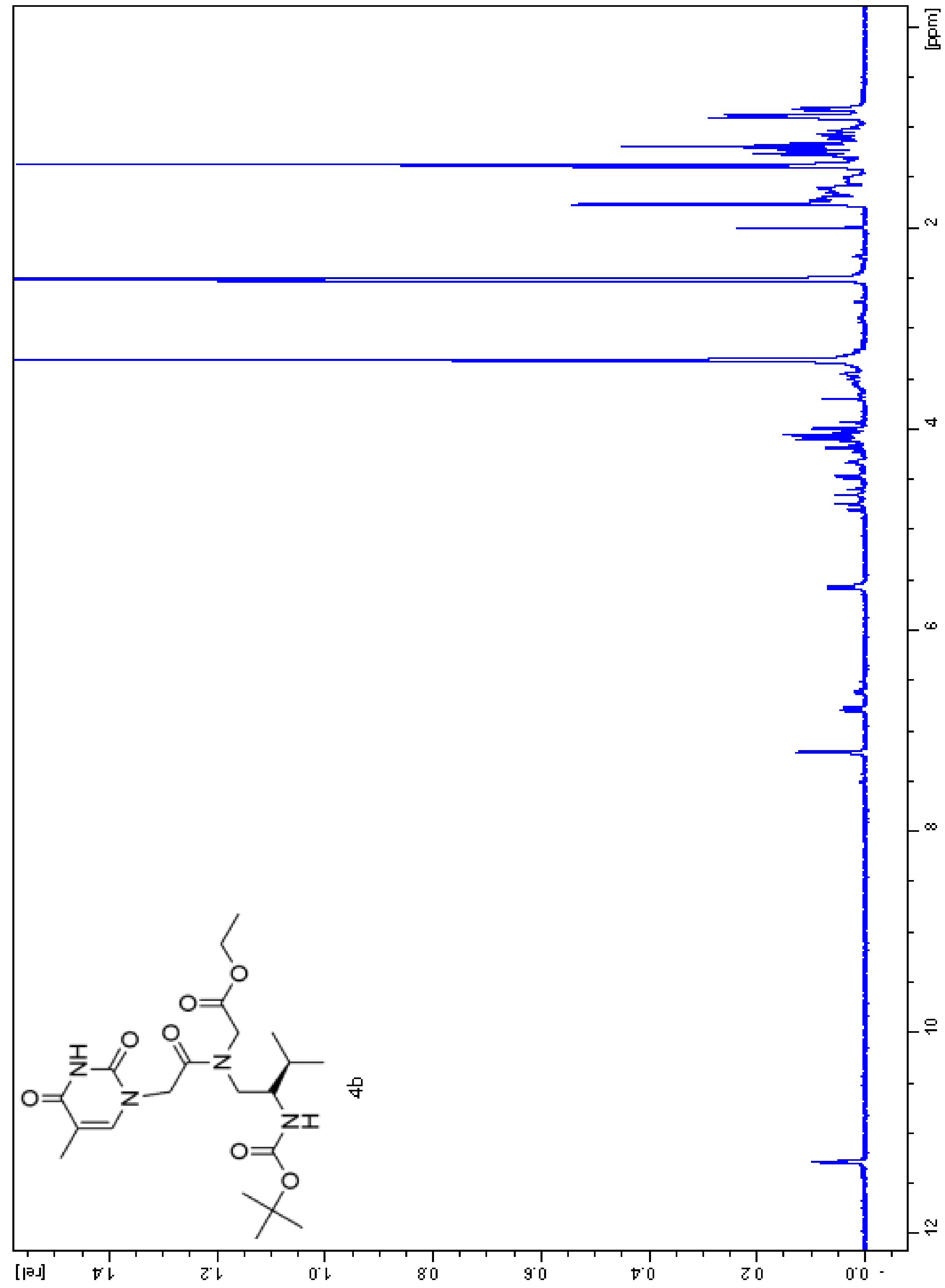

^{1}H NMR

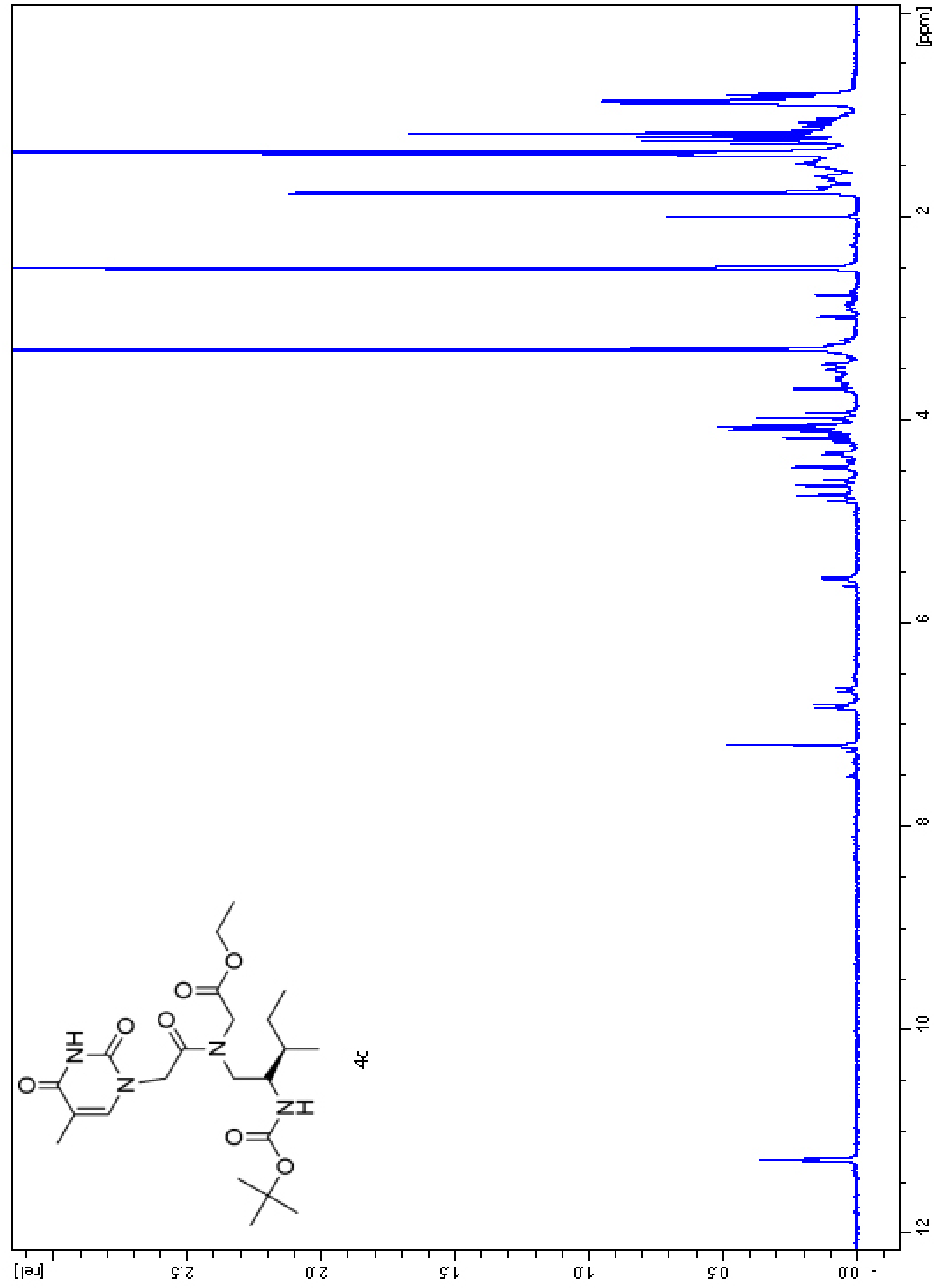

^{1}H NMR

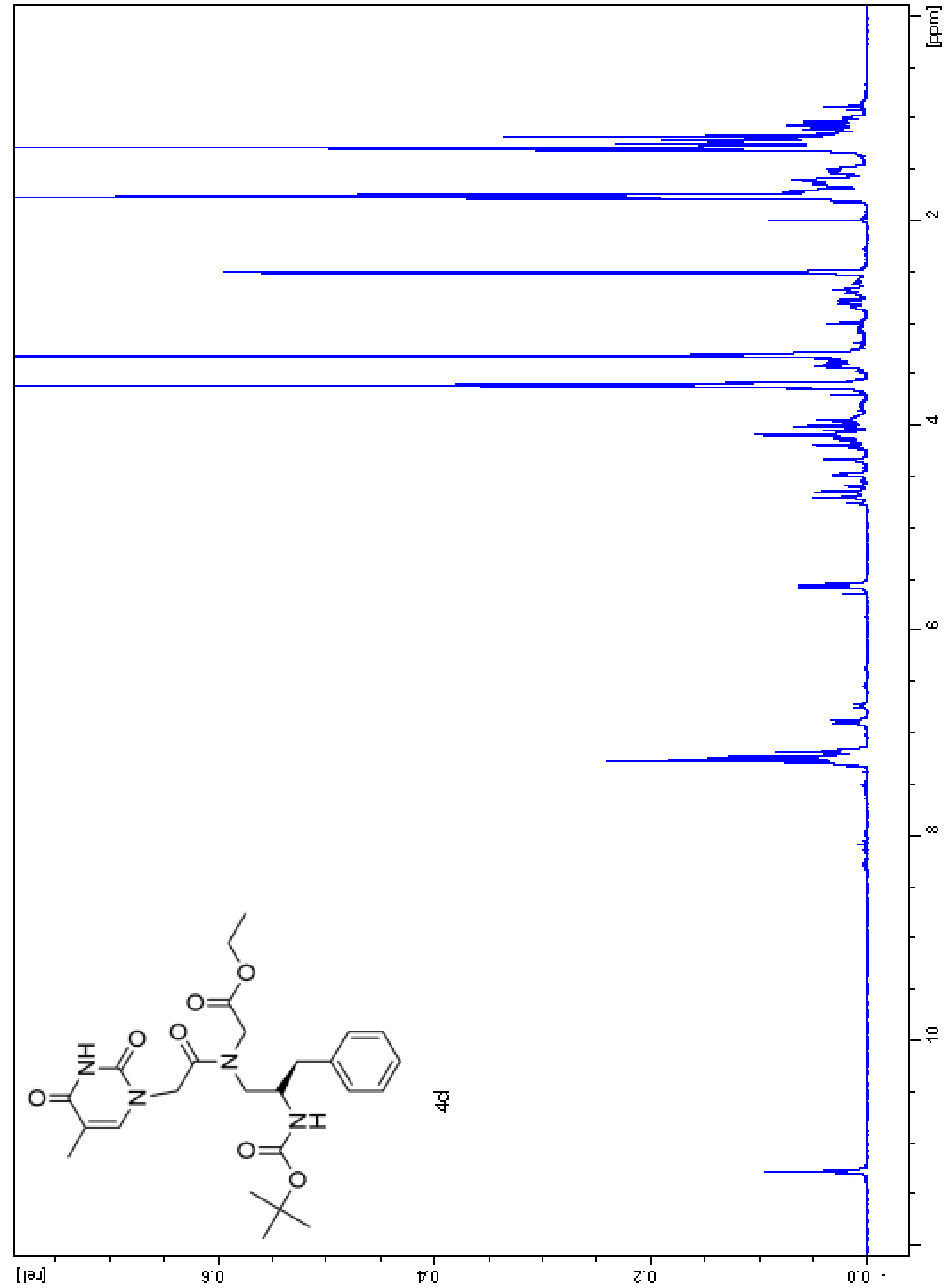

^{1}H NMR

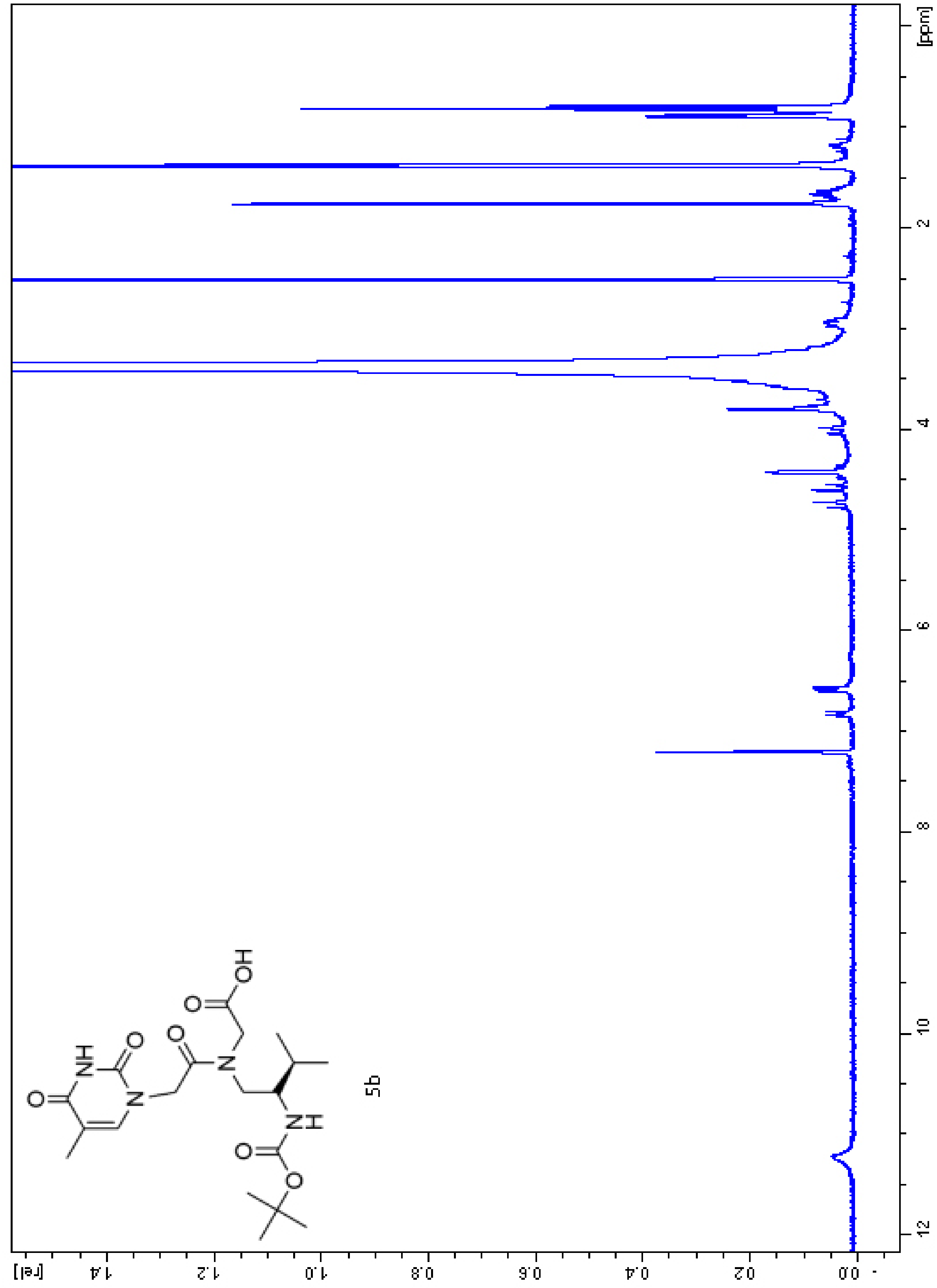

^{13}C NMR

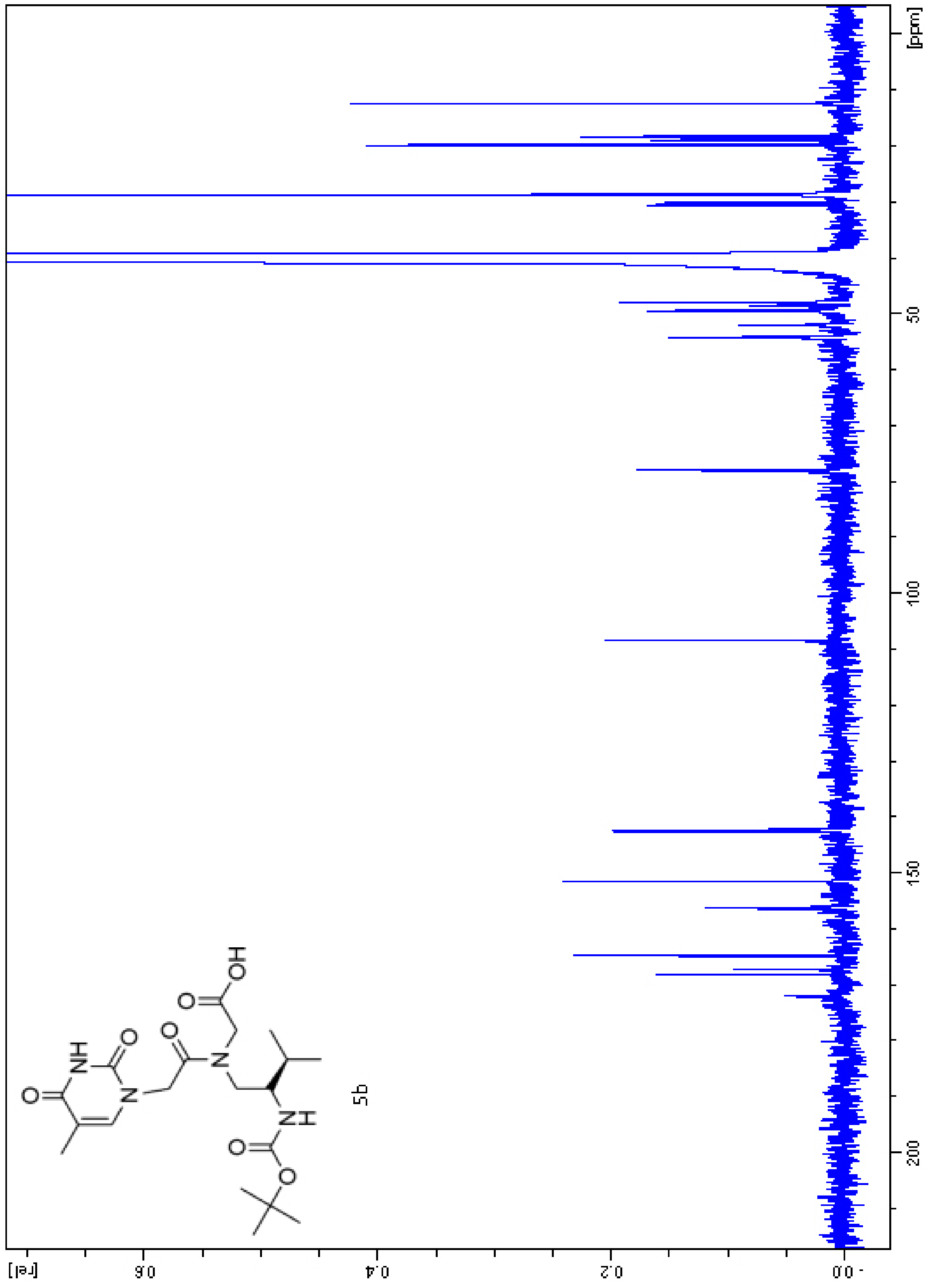

^{1}H NMR

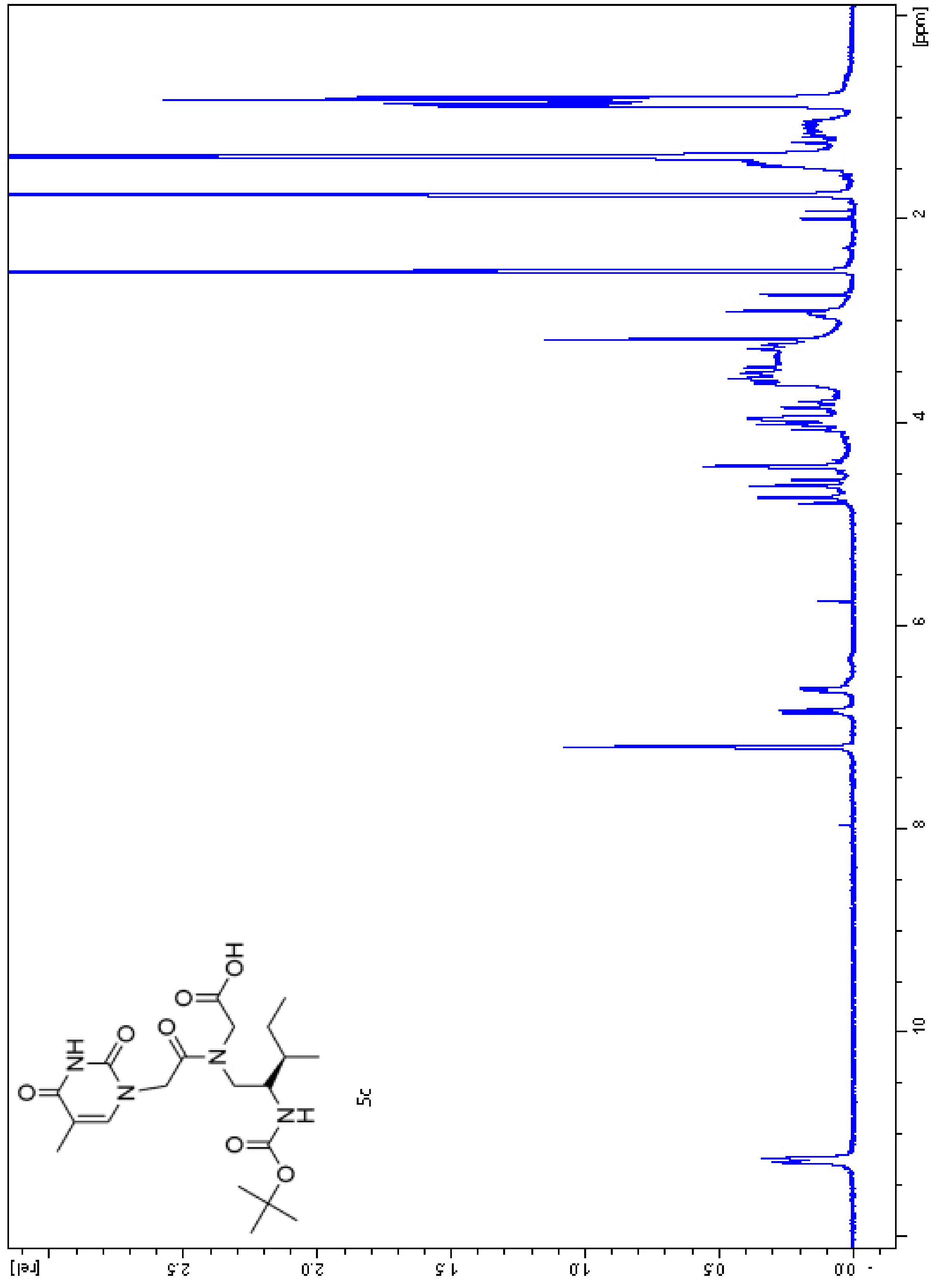

^{13}C NMR

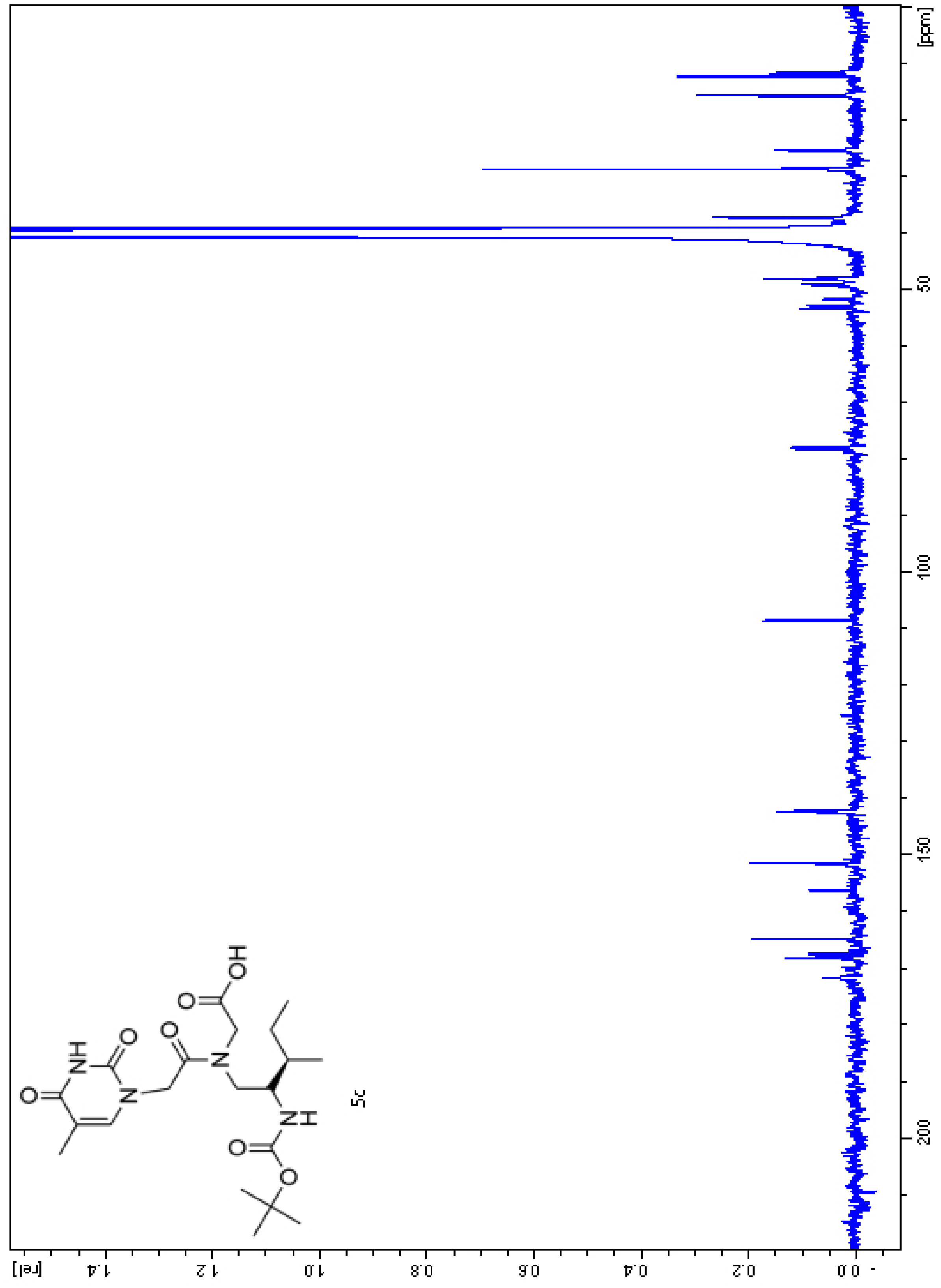

^{1}H NMR

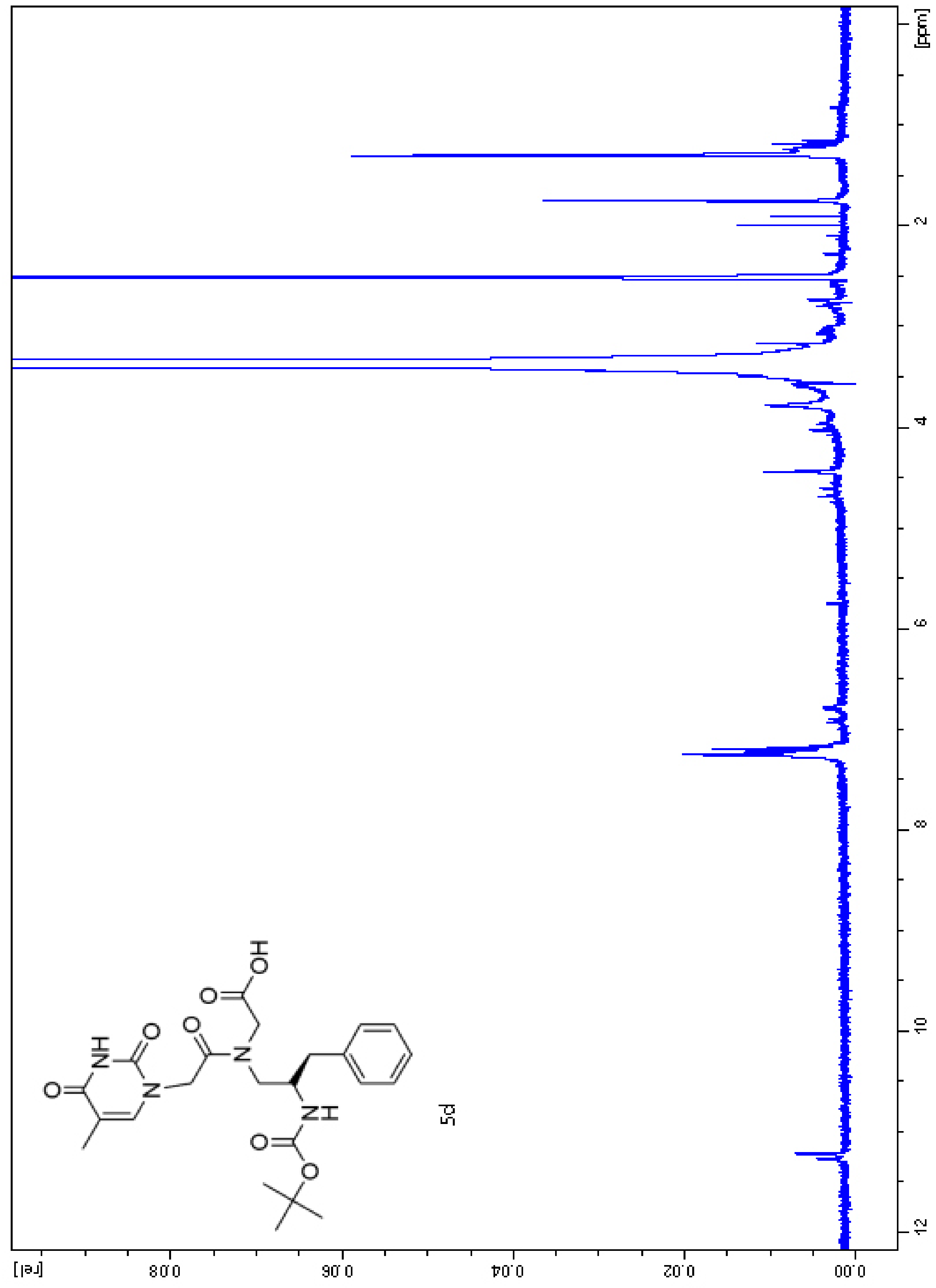

^{13}C NMR

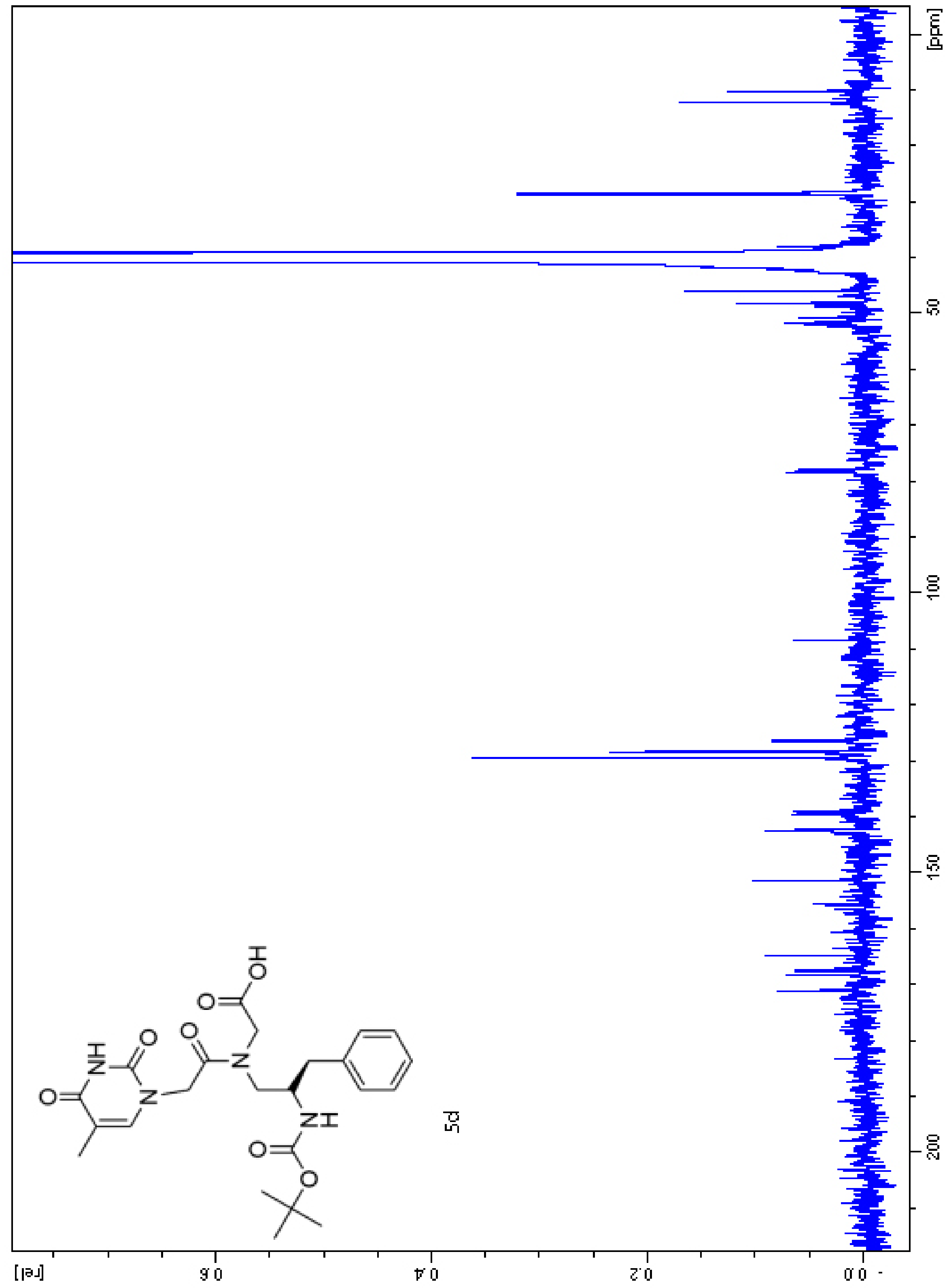

Supplement: Supplementary file 1 [file 652702.f1.pdf]
