# Supplementary material for: Effect of Steric Constraint at the γ-Backbone Position on the Conformations and Hybridization Properties of PNAs
Source: J Nucleic Acids. 2011 May 25;2011:652702. doi: 10.4061/2011/652702 (PMC3138043; doi:10.4061/2011/652702)

Effect of Steric Constraint at the γ-Backbone Position on the Conformations and Hybridization Properties of PNAs

Matthew J. Crawford, Srinivas Rapireddy, Raman Bahal, Iulia Sacui, and Danith H. Ly*

Department of Chemistry and Center for Nucleic Acids Science and Technology (CNAST), Carnegie Mellon University,
4400 Fifth Avenue, Pittsburgh, PA 15213

PNA1: H-GCATGTTTGA-^{L}Lys-NH2 (Unmodified PNA)

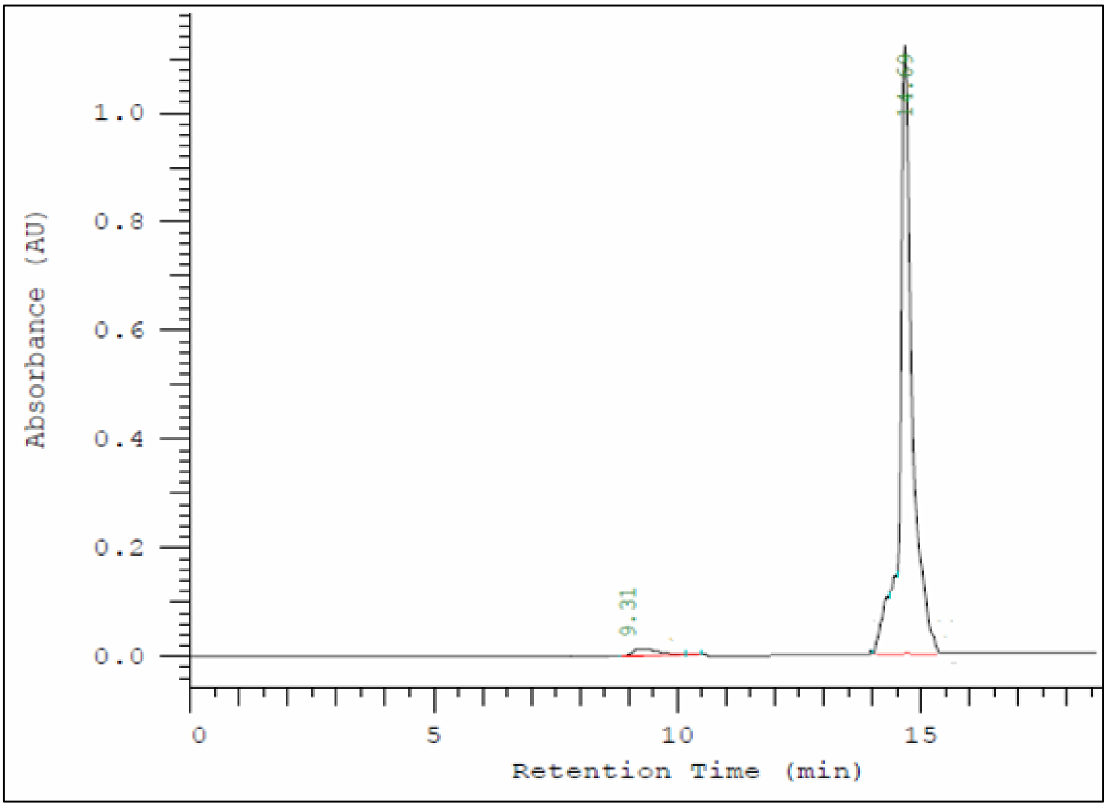

Figure S1a. Reinjected HPLC trace of PNA1. Eluent A: 0.1% TFA in water, and eluent B: 0.1% TFA in ACN. The gradient was 0-40% of eluent B in 40 minutes at 45°C with a flow rate of 3.0 mL/min.

Calculated Mass: 2898.1

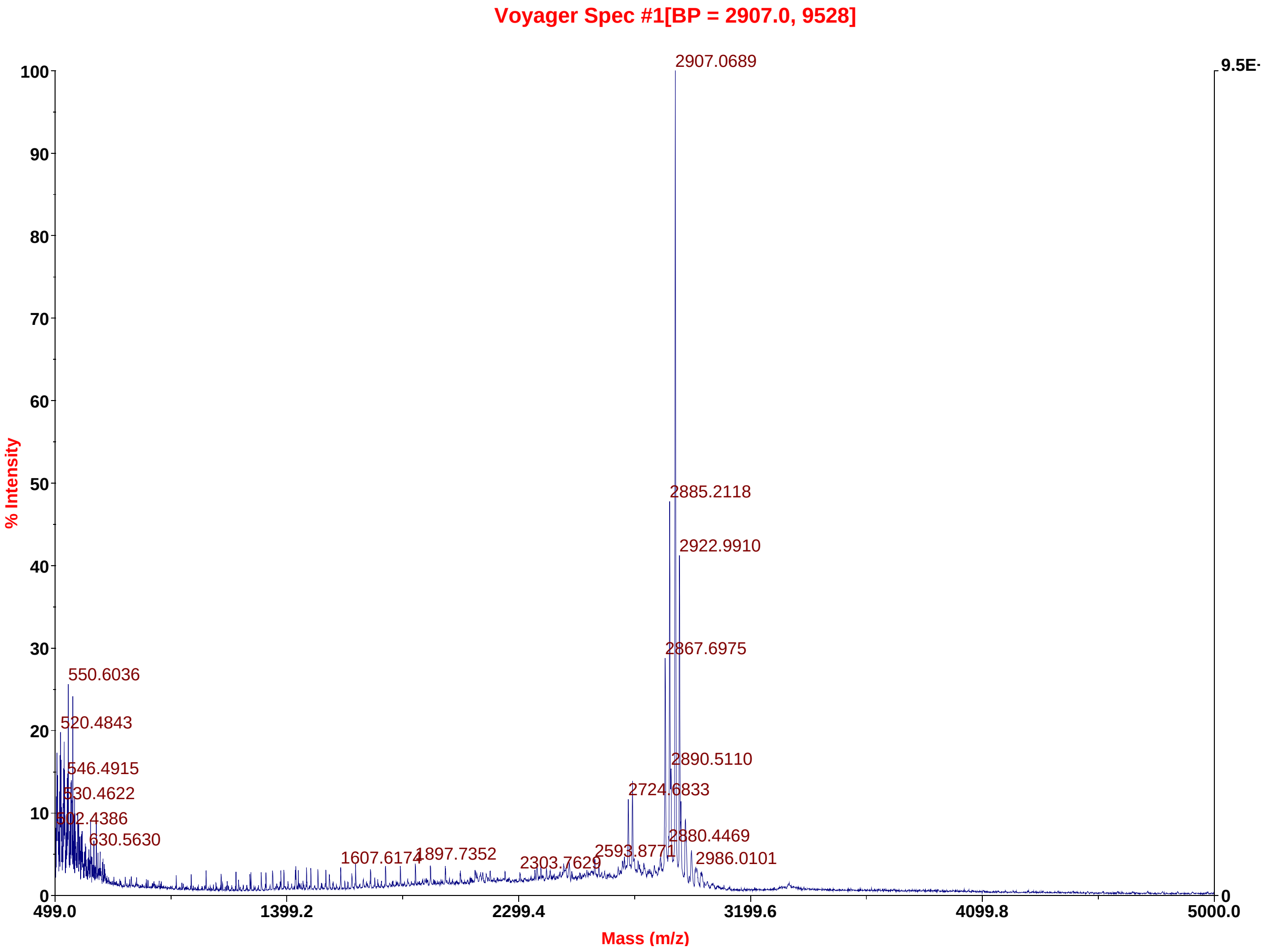

Figure S1b. MALDI-TOF spectrum of PNA1.

PNA2: H-GCATG**T**TTGA-[L]Lys-NH2 (Alanine PNA)

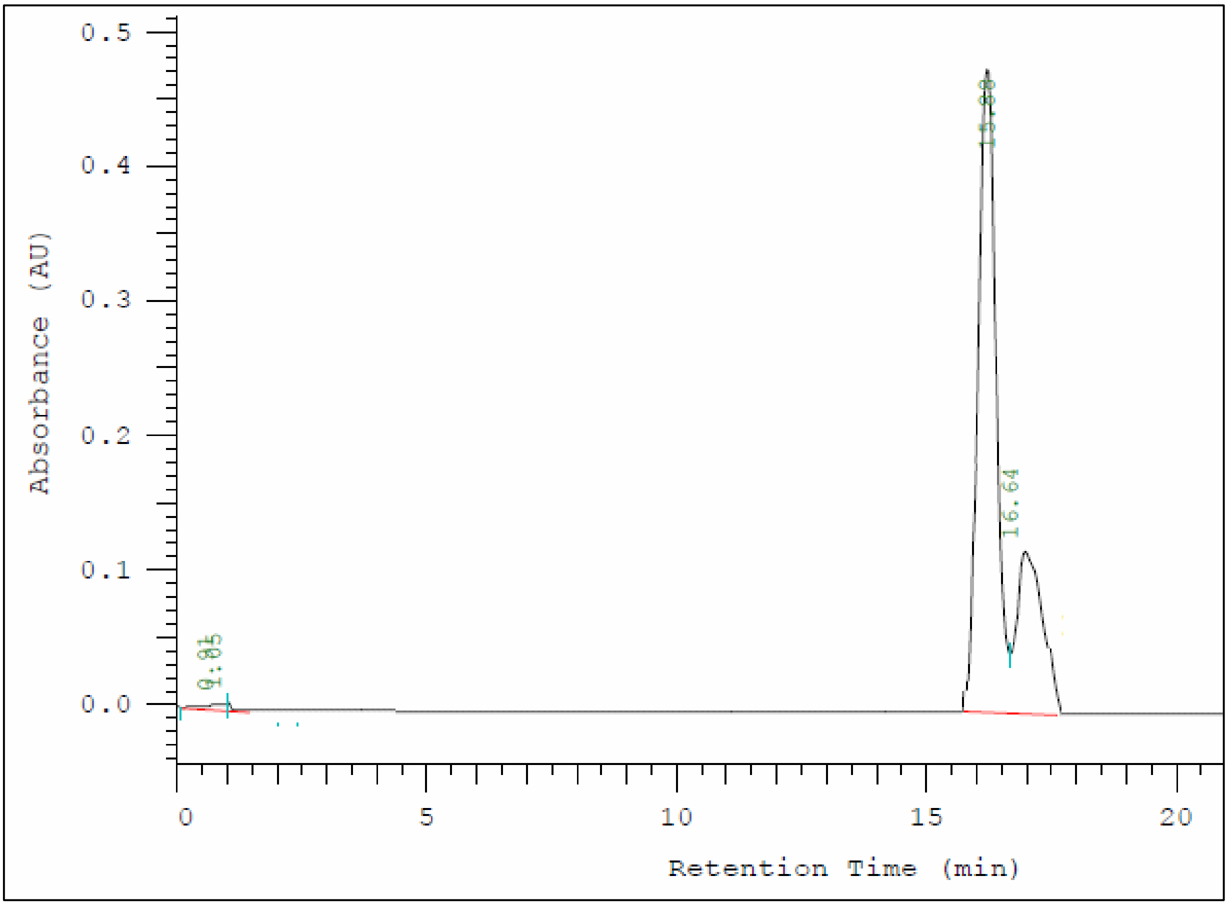

Figure S2a. Reinjected HPLC trace of PNA2.

Calculated Mass: 2912.1

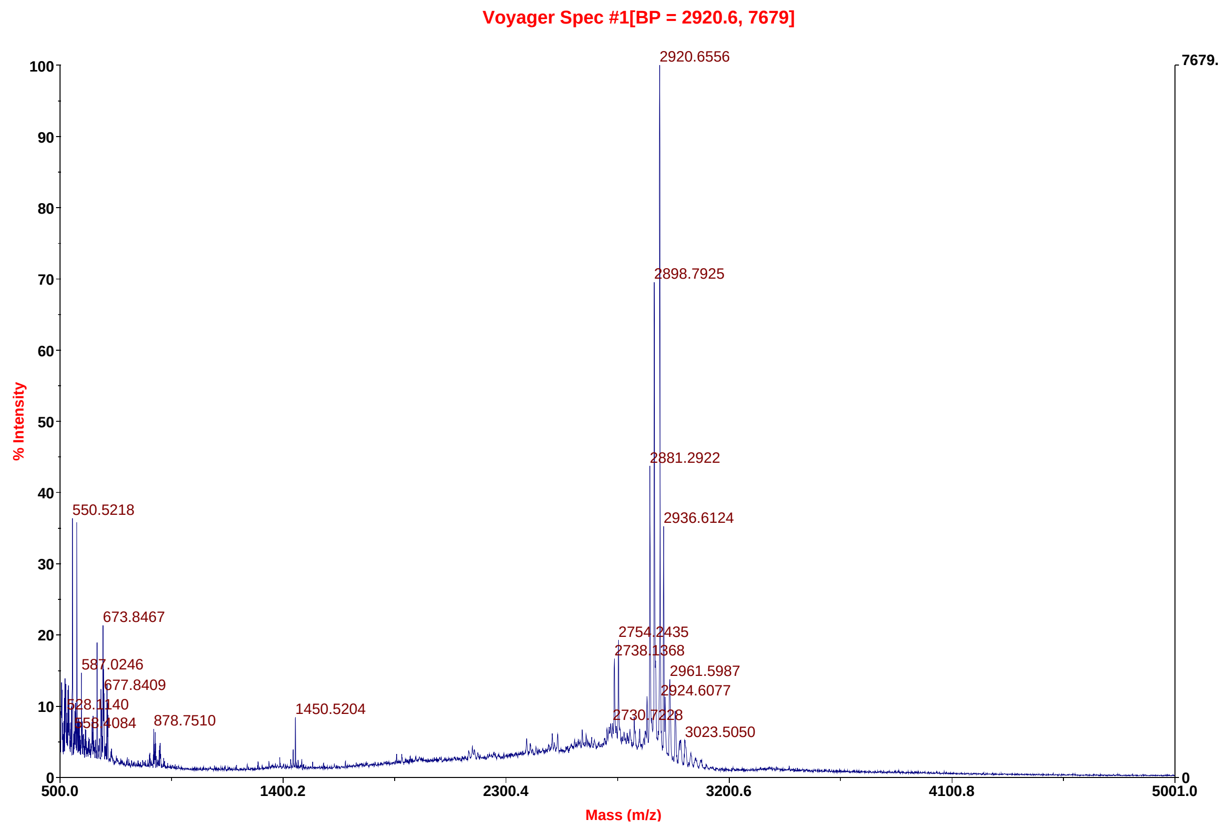

Figure S2b. MALDI-TOF spectrum of PNA2.

PNA 3: H-GCATG**T**TTGA-^{L}Lys-NH2 (Valine PNA)

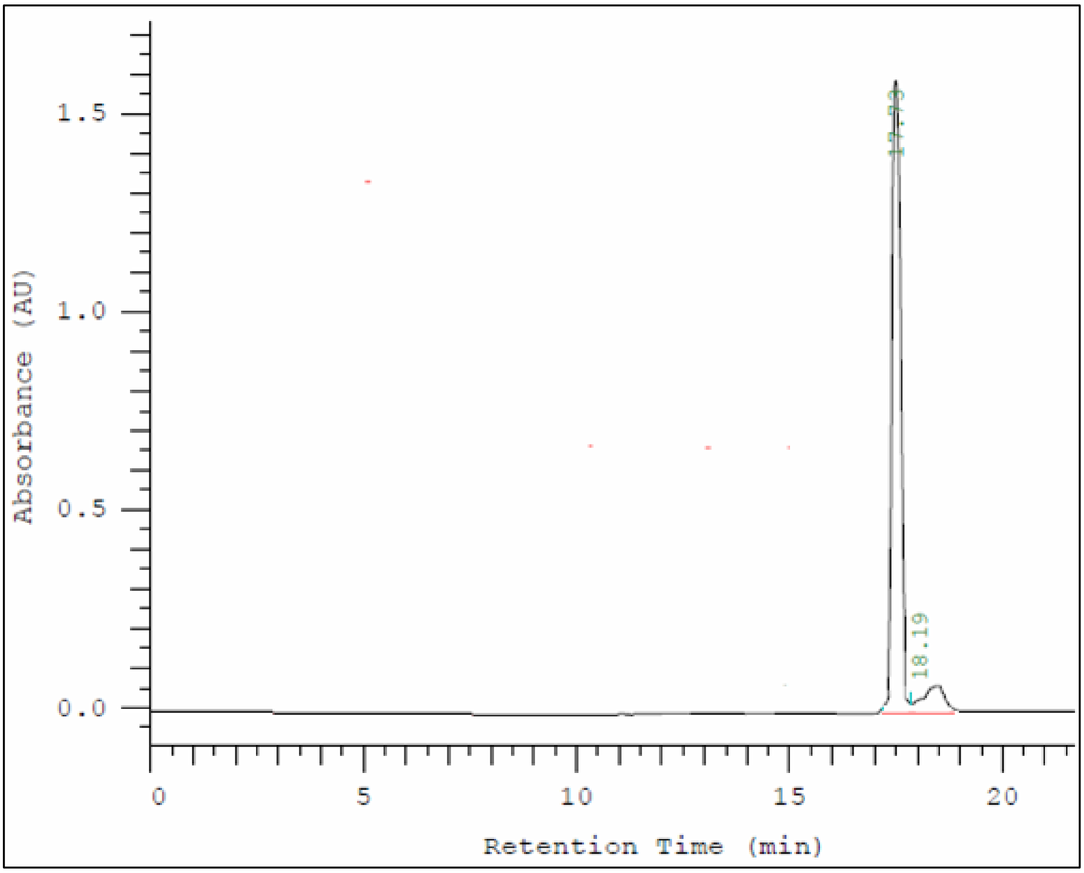

Figure S3a. Reinjected HPLC trace of PNA3.

Calculated Mass: 2941.1

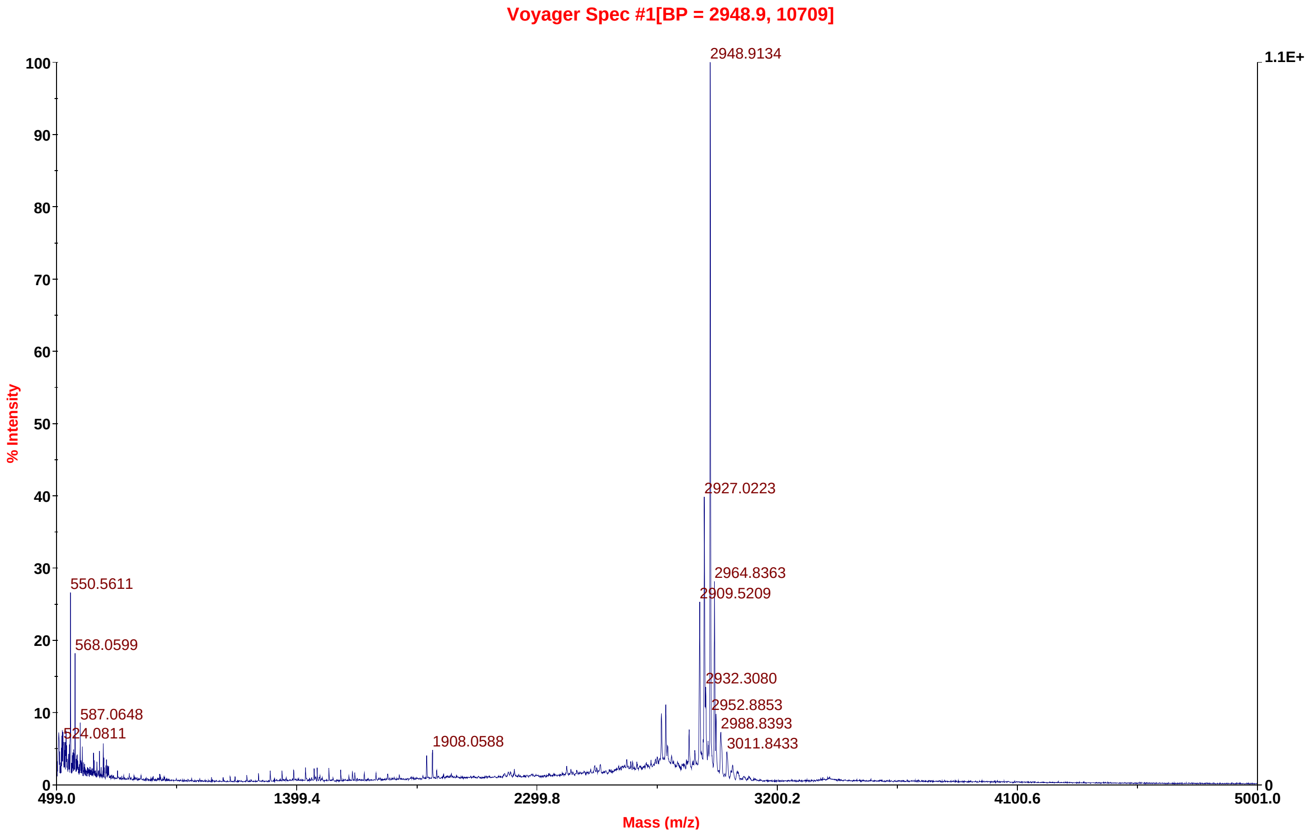

Figure S3b. MALDI-TOF spectrum of PNA3.

PNA 4: H-GCATG**T**TTGA-^{L}Lys-NH2 (Isoleucine PNA)

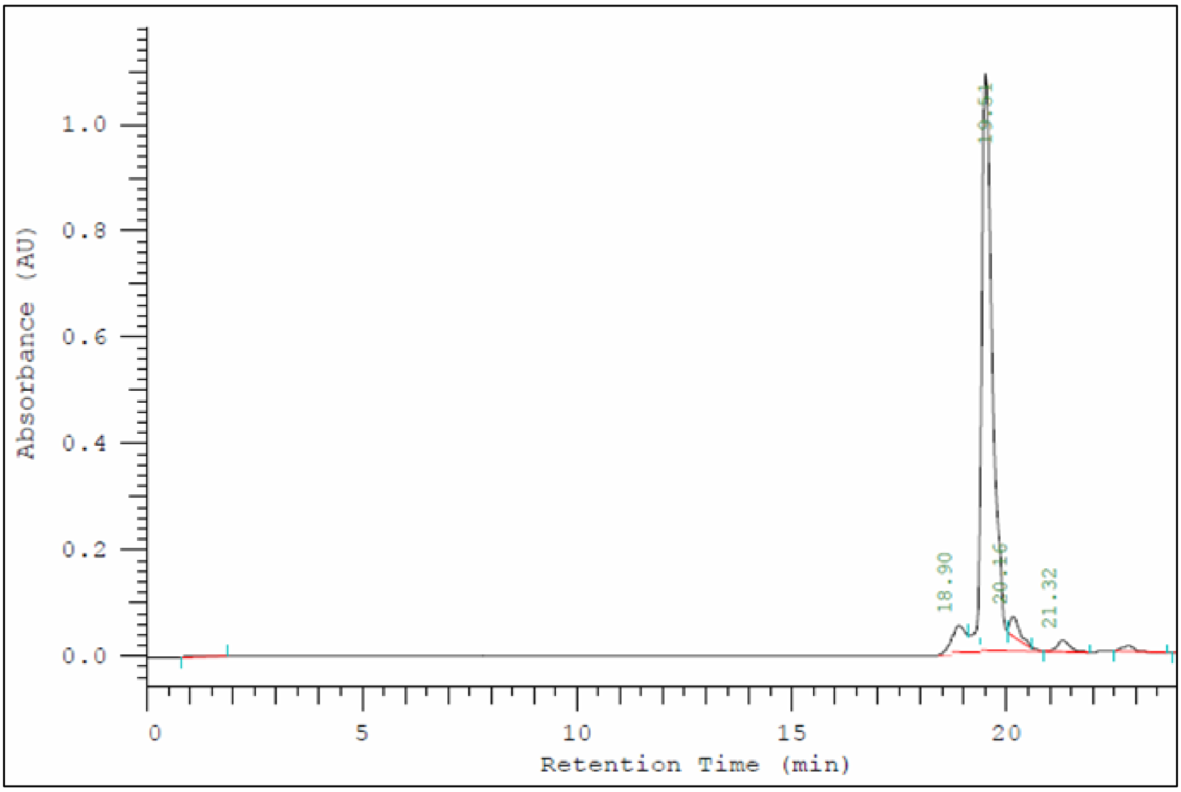

Figure S4a. Reinjected HPLC trace of PNA4.

Calculated Mass: 2955.1

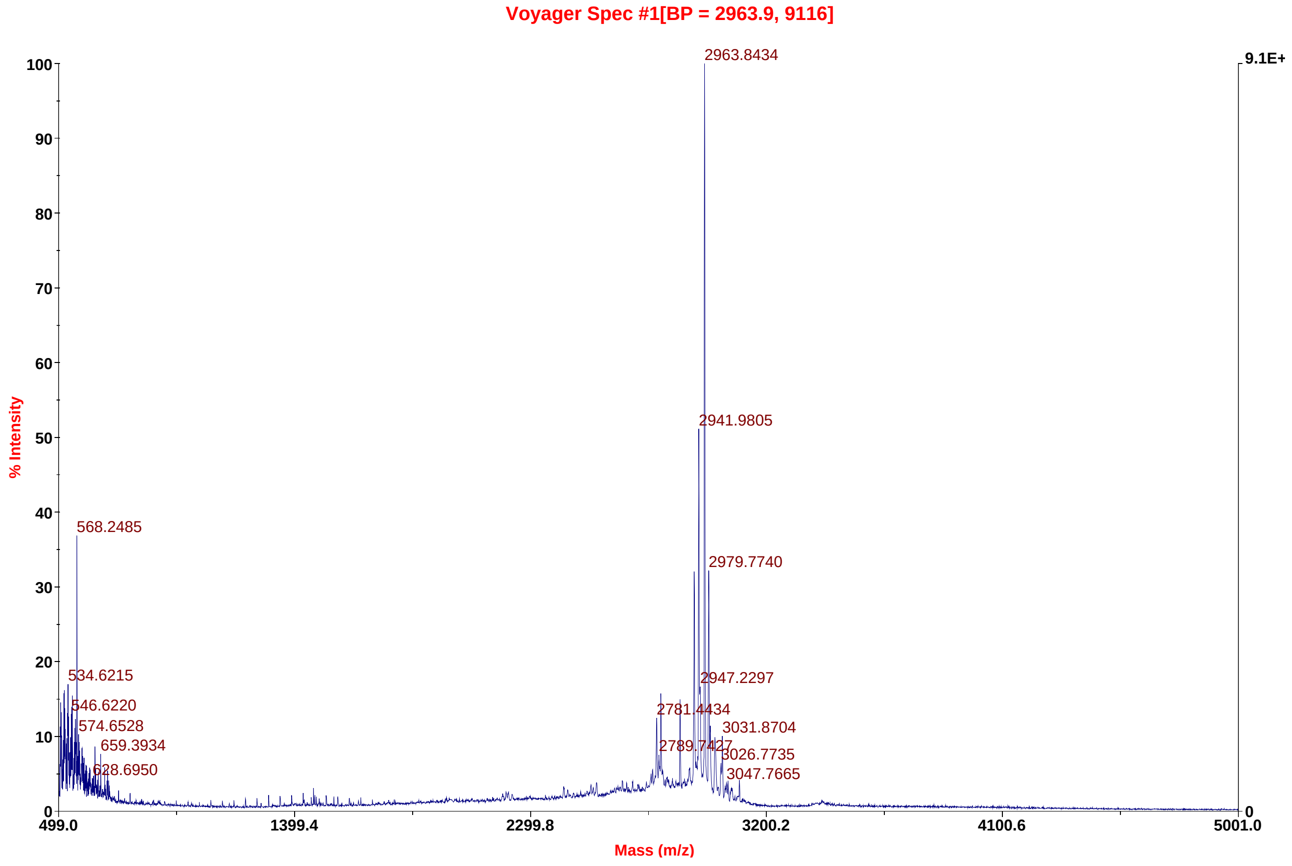

Figure S4b. MALDI-TOF spectrum of PNA4.

PNA 5: H-GCATG**T**TTGA-^{L}Lys-NH2 (Phenylalanine PNA)

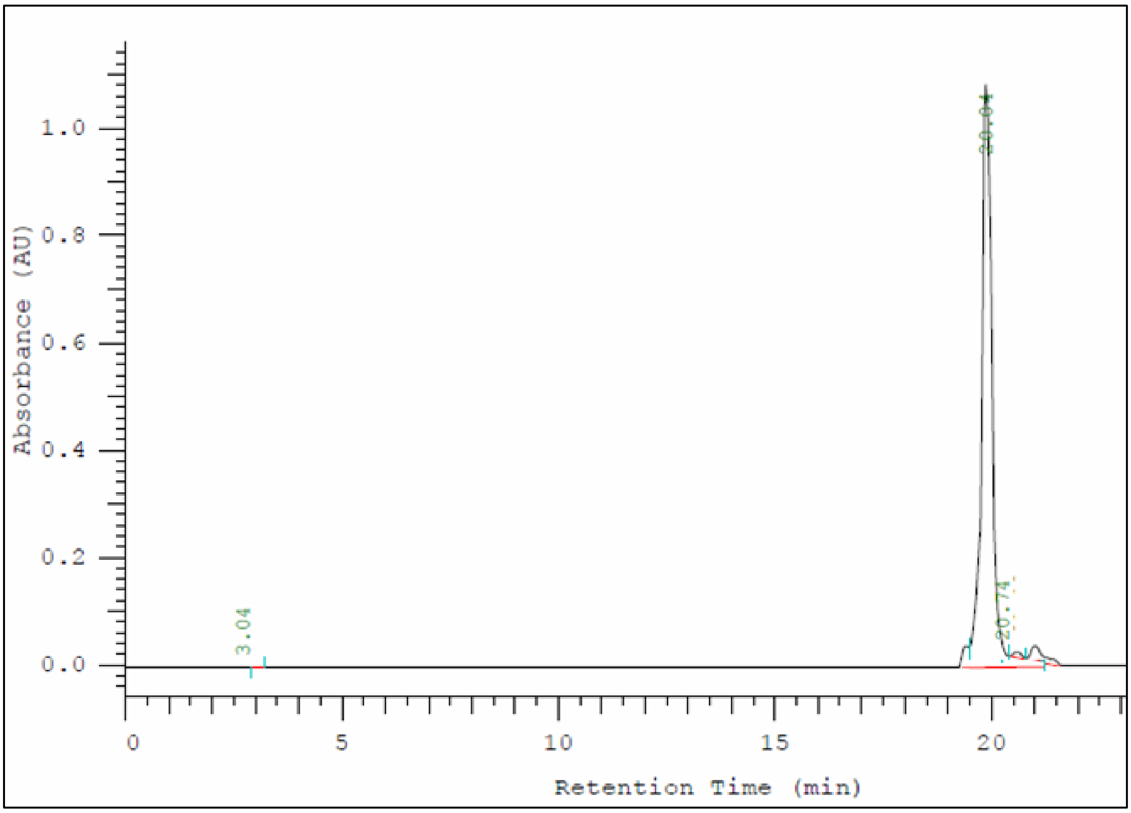

Figure S5a. Reinjected HPLC trace of PNA5.

Calculated Mass: 2994.2

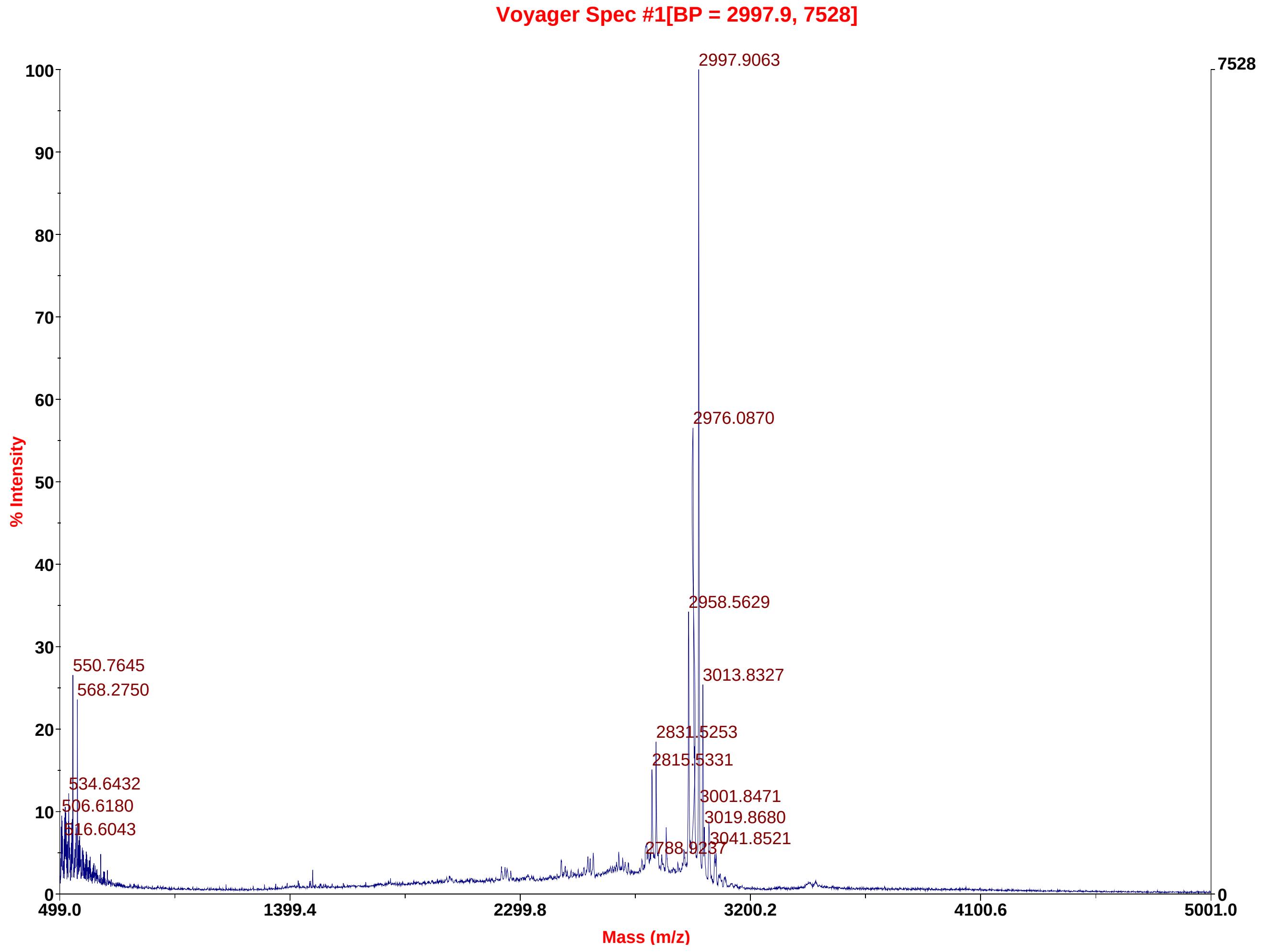

Figure S5b. MALDI-TOF spectrum of PNA5.

PNA 6: H-GCA**T**G**T**T**T**GA-^{L}Lys-NH2 (Phenylalanine PNA)

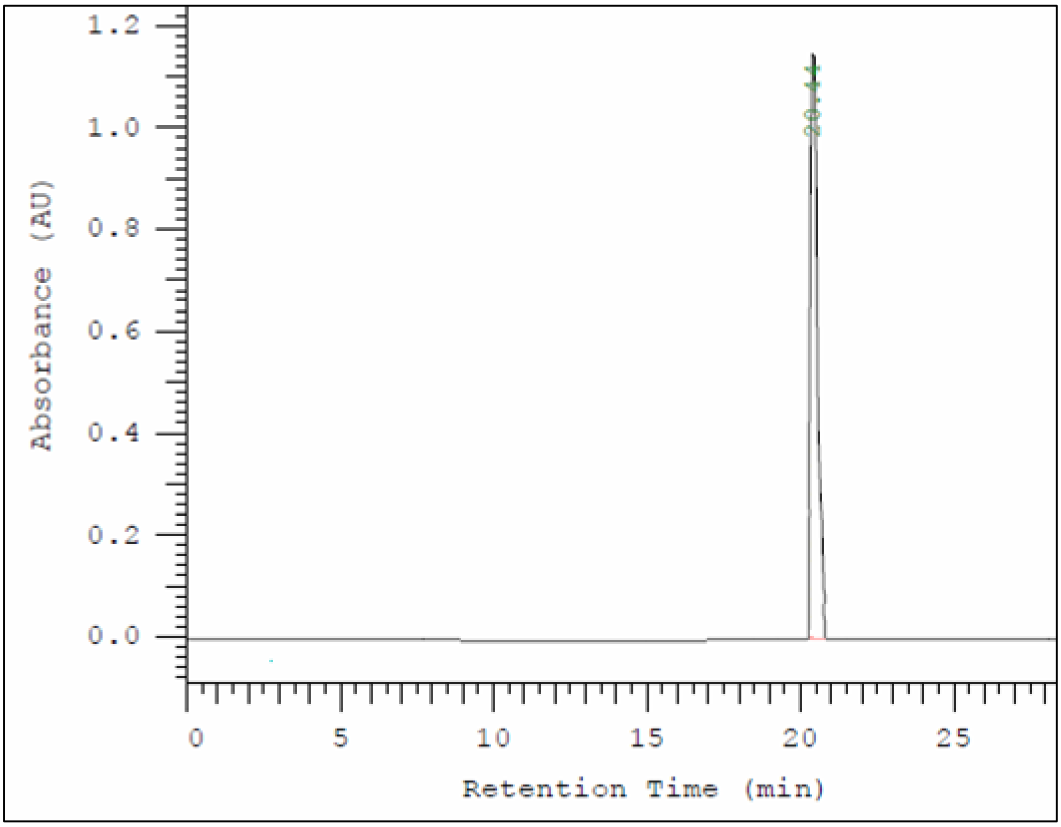

Figure S6a. Reinjected HPLC trace of PNA6.

Calculated Mass: 3176.2

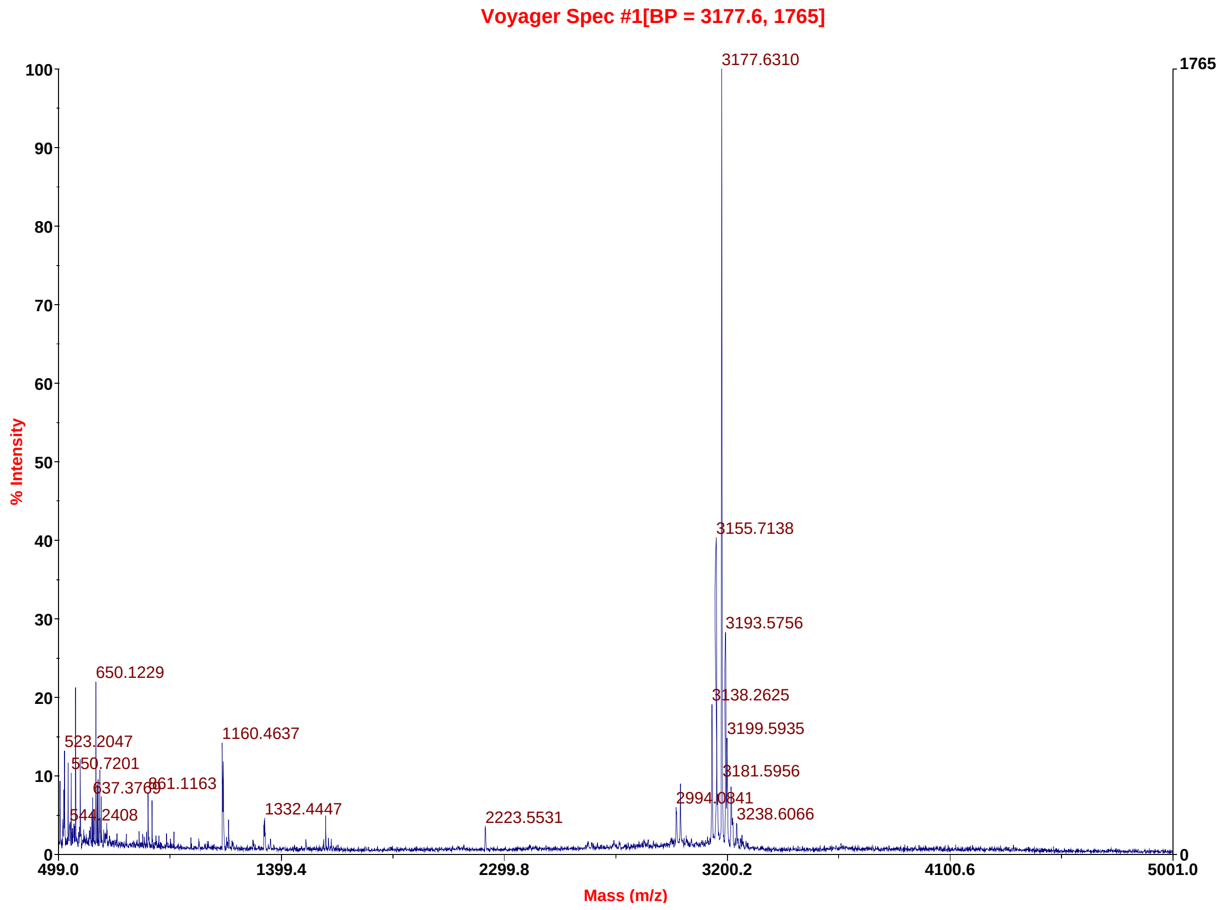

Figure S6b. MALDI-TOF spectrum of PNA6.

PNA 7: H-GCATG**TTT**GA-^{L}Lys-NH2 (Phenylalanine PNA)

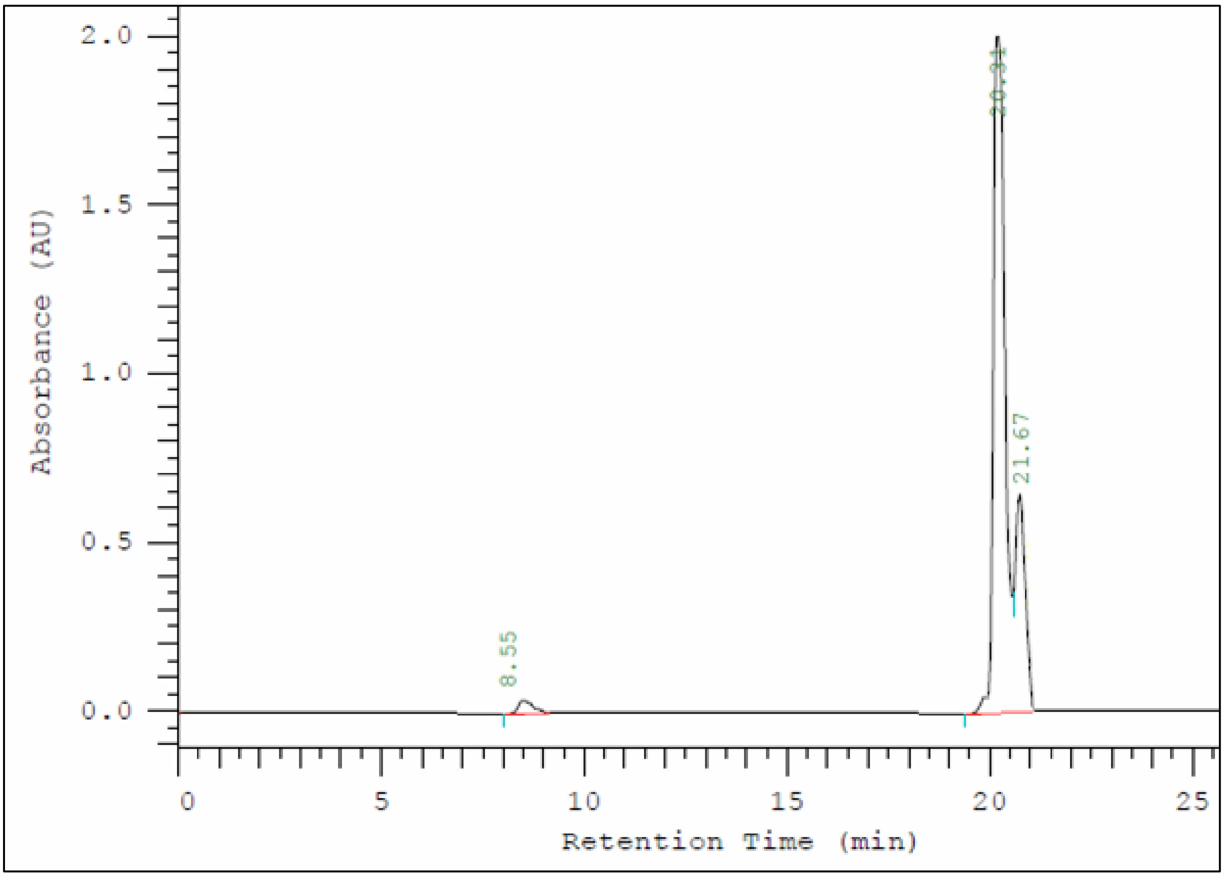

Figure S7a. Reinjected HPLC trace of PNA7.

Calculated Mass: 3176.2

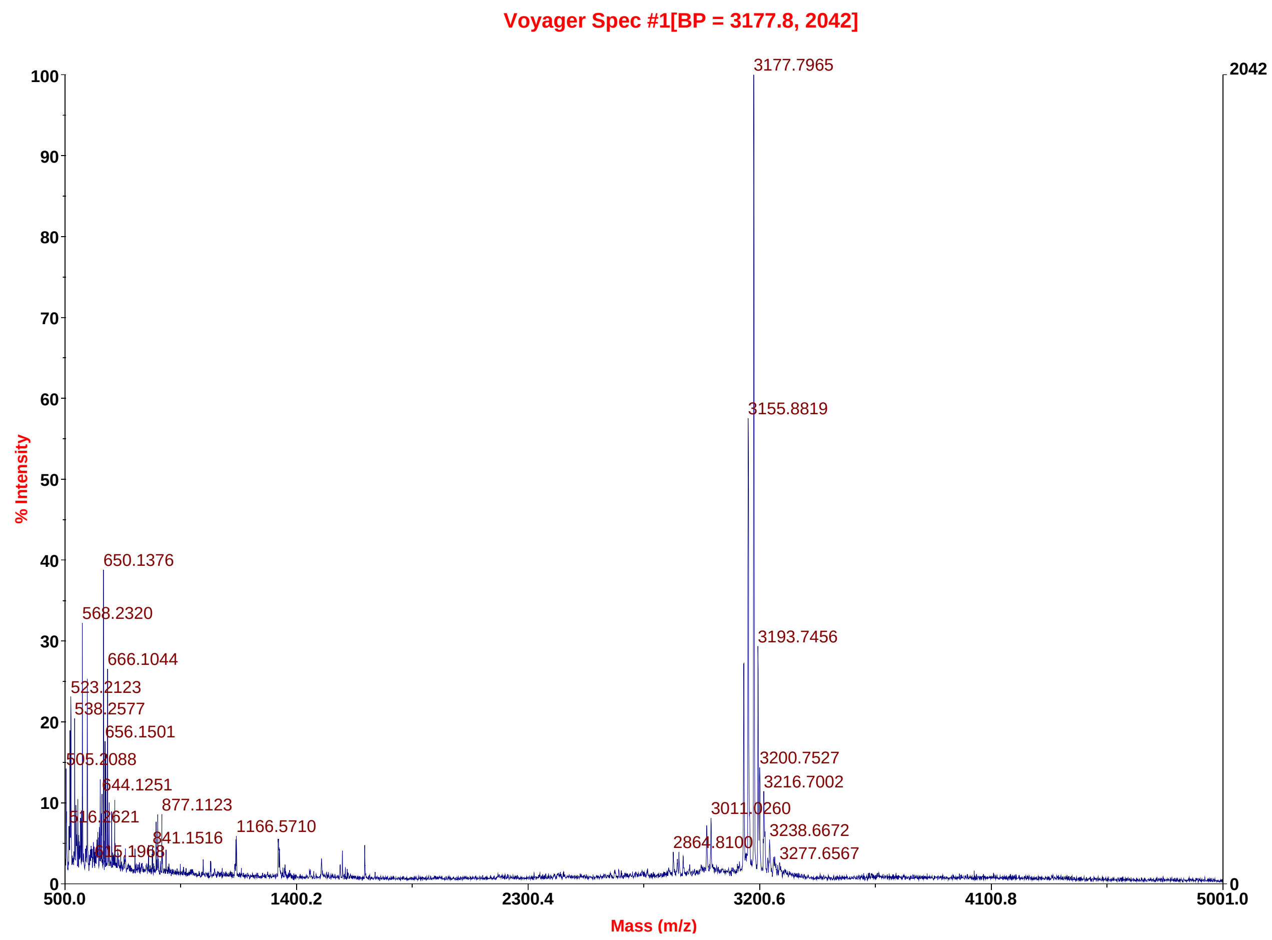

Figure S7b. MALDI-TOF spectrum of PNA7.

PNA 8: H-GCA**T**G**T**T**T**GA-^{L}Lys-NH2 (Valine PNA)

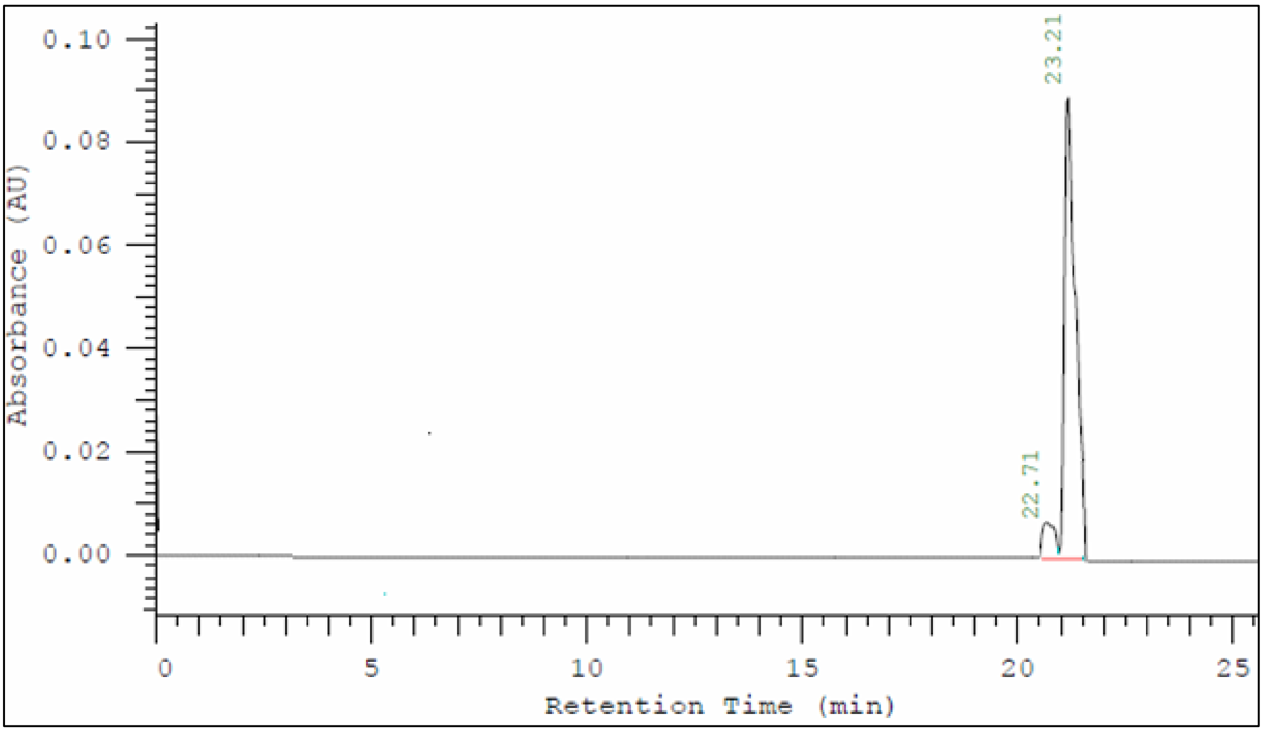

Figure S8a. Reinjected HPLC trace of PNA8.

Calculated Mass: 3029.1

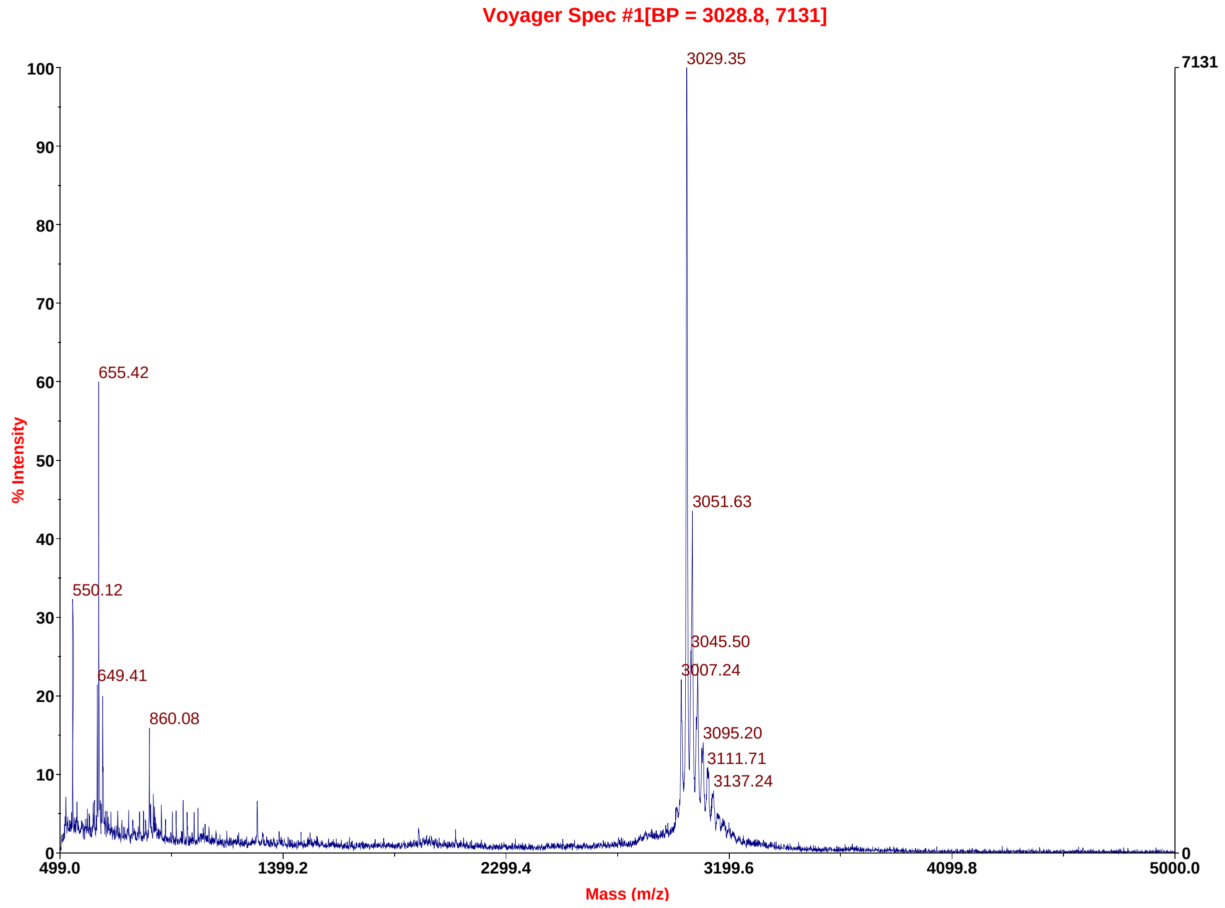

Figure S8b. MALDI-TOF spectrum of PNA8.

PNA 9: H-GCATG<u>**TTT**</u>GA-^{L}Lys-NH2 (Valine PNA)

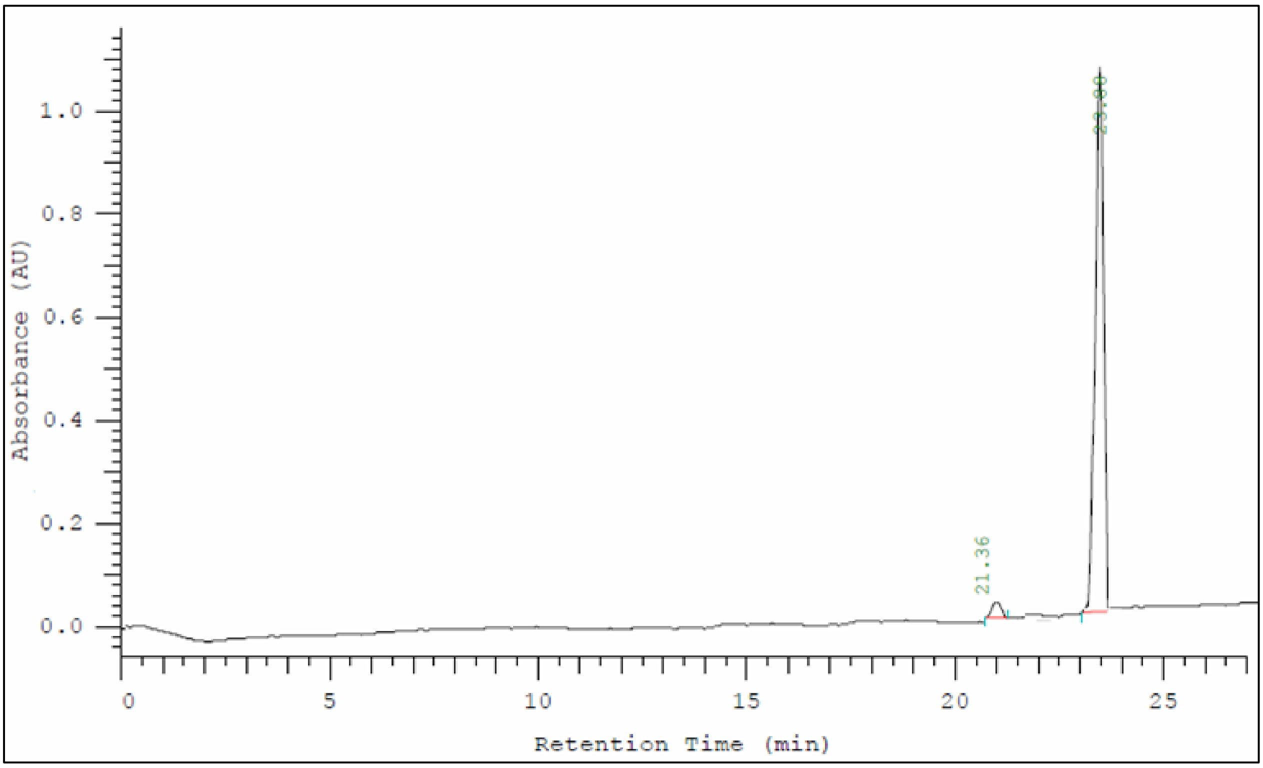

Figure S9a. Reinjected HPLC trace of PNA9.

Calculated Mass: 3029.1

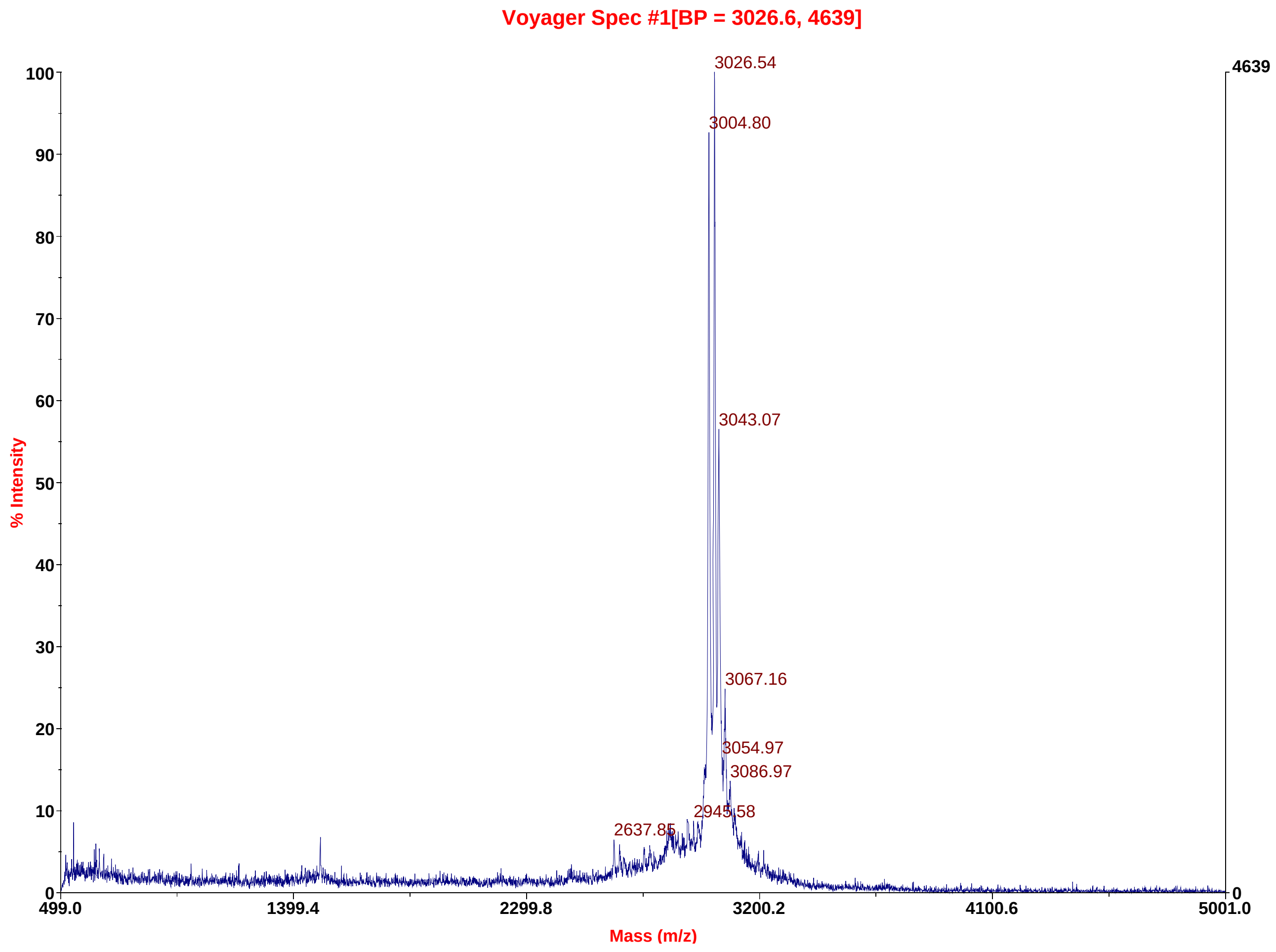

Figure S9b. MALDI-TOF spectrum of PNA9.

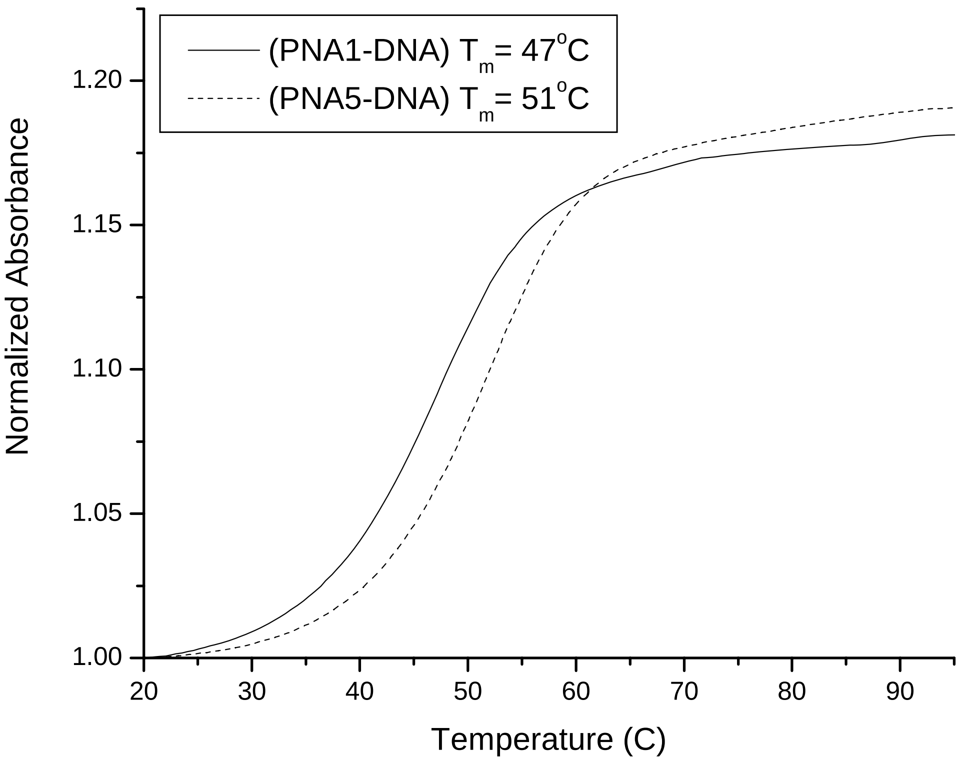

Figure S10. UV-melting curves of PNA1-DNA and PNA5-DNA duplexes containing perfectly matched sequences. The samples were prepared in buffer containing 0.1 mM EDTA, 100 mM NaCl, 10 mM sodium phosphate (pH 7.0) at 5μM duplex strand concentration each. The T_ms were determined by taking the first derivatives of the UV-melting curves.

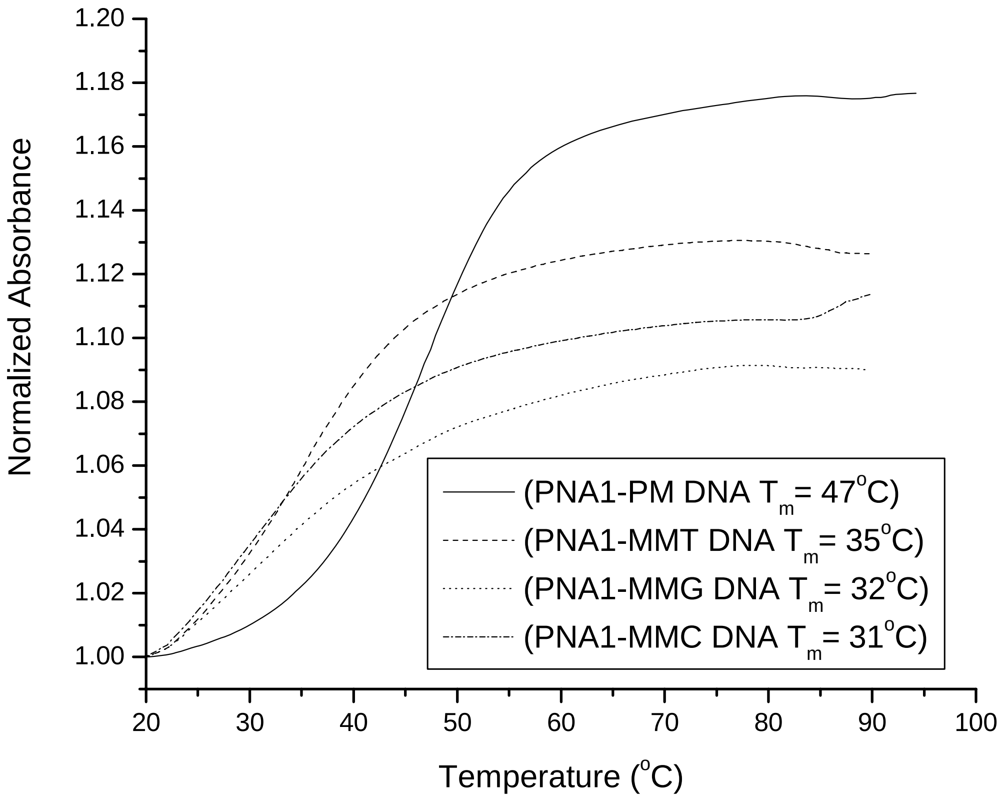

Figure S11. UV-melting curves of PNA1-DNA duplexes containing perfectly matched (PM) and mismatch (MM) sequences. The samples were prepared in buffer containing 0.1 mM EDTA, 100 mM NaCl, 10 mM sodium phosphate (pH 7.0) at 5μM duplex strand concentration each. The T_ms were determined by taking the first derivatives of the UV-melting curves.

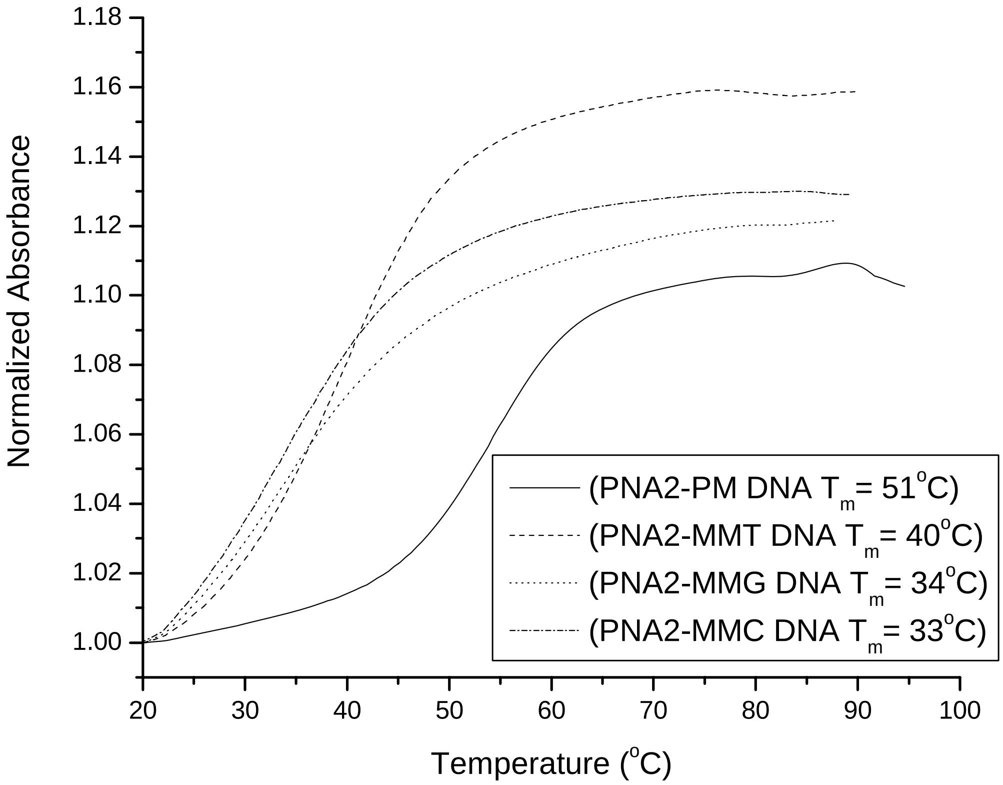

Figure S12. UV-melting curves of PNA2-DNA duplexes containing perfectly matched (PM) and mismatch (MM) sequences. The samples were prepared in buffer containing 0.1 mM EDTA, 100 mM NaCl, 10 mM sodium phosphate (pH 7.0) at 5µM duplex strand concentration each. The T_ms were determined by taking the first derivatives of the UV-melting curves.

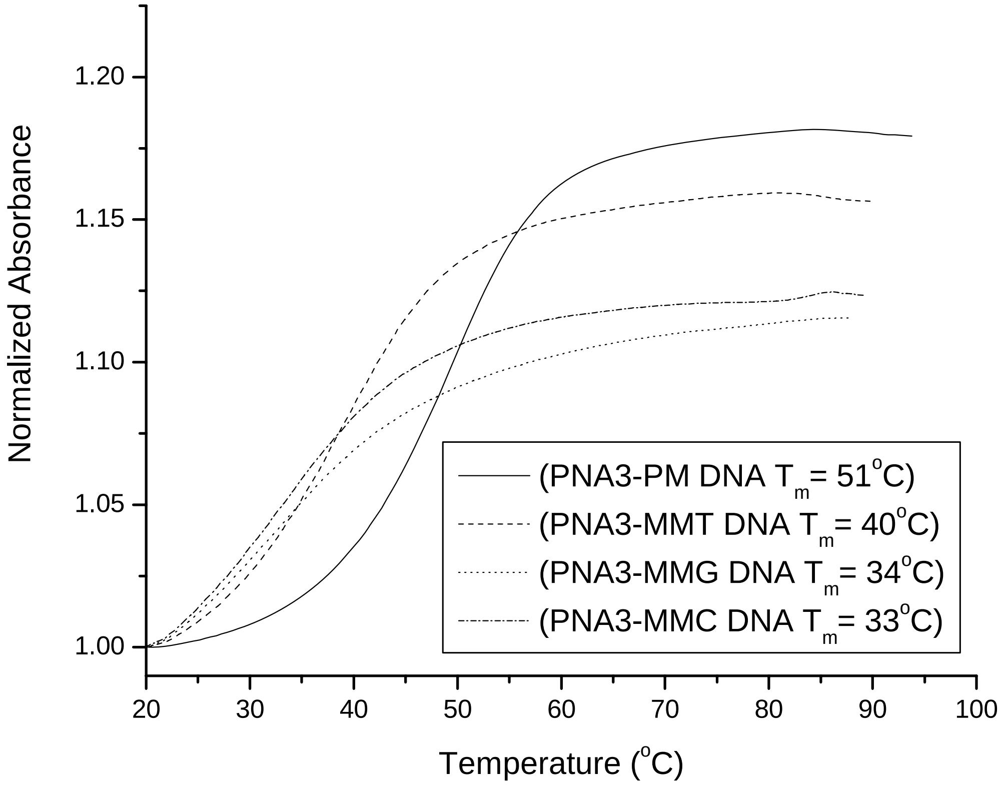

Figure S13. UV-melting curves of PNA3-DNA duplexes containing perfectly matched (PM) and mismatch (MM) sequences. The samples were prepared in buffer containing 0.1 mM EDTA, 100 mM NaCl, 10 mM sodium phosphate (pH 7.0) at 5μM duplex strand concentration each. The T_ms were determined by taking the first derivatives of the UV-melting curves.

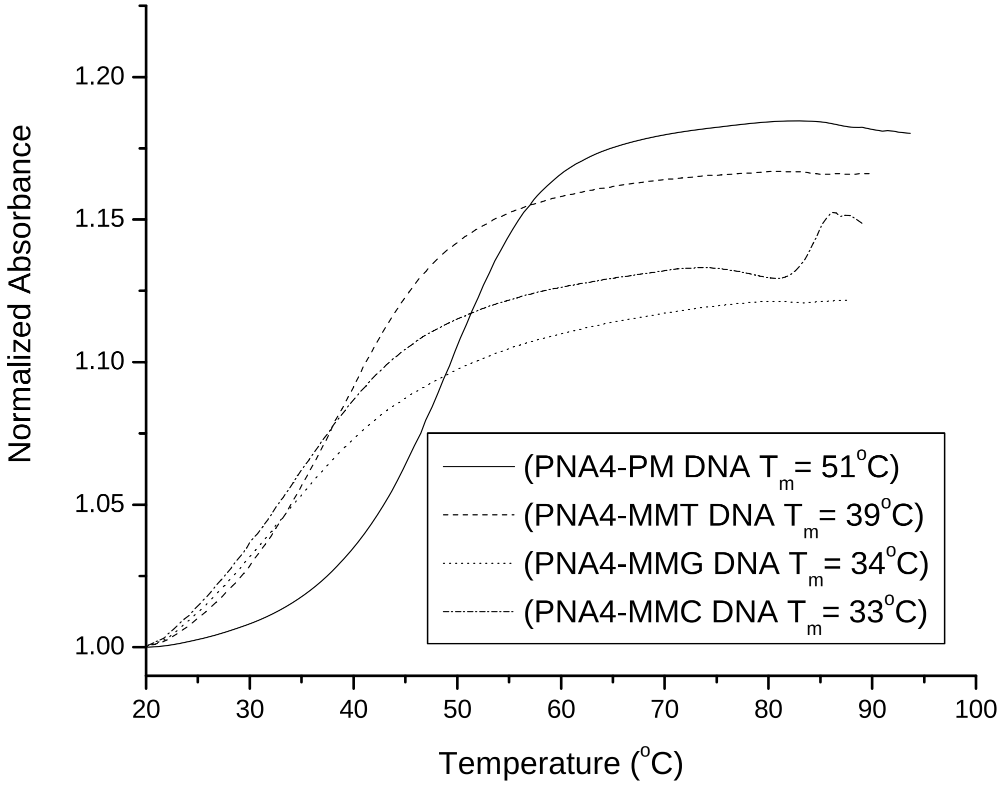

Figure S14. UV-melting curves of PNA4-DNA duplexes containing perfectly matched (PM) and mismatch (MM) sequences. The samples were prepared in buffer containing 0.1 mM EDTA, 100 mM NaCl, 10 mM sodium phosphate (pH 7.0) at 5μM duplex strand concentration each. The T_ms were determined by taking the first derivatives of the UV-melting curves.

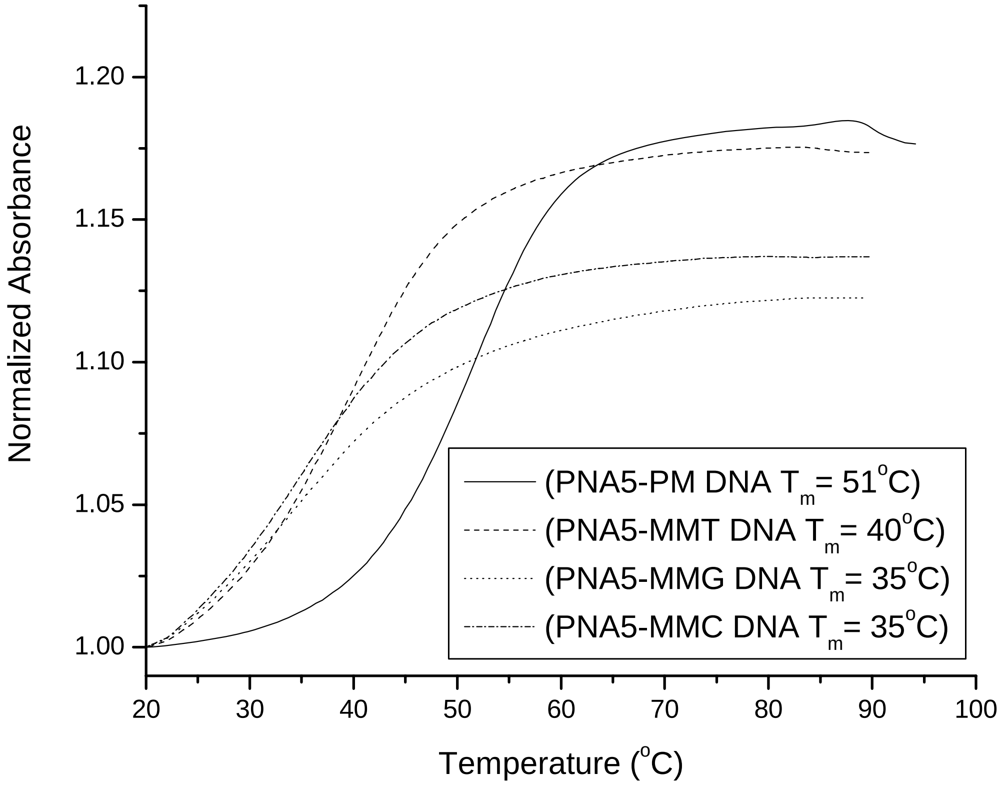

Figure S15. UV-melting curves of PNA5-DNA duplexes containing perfectly matched (PM) and mismatch (MM) sequences. The samples were prepared in buffer containing 0.1 mM EDTA, 100 mM NaCl, 10 mM sodium phosphate (pH 7.0) at 5μM duplex strand concentration each. The T_ms were determined by taking the first derivatives of the UV-melting curves.

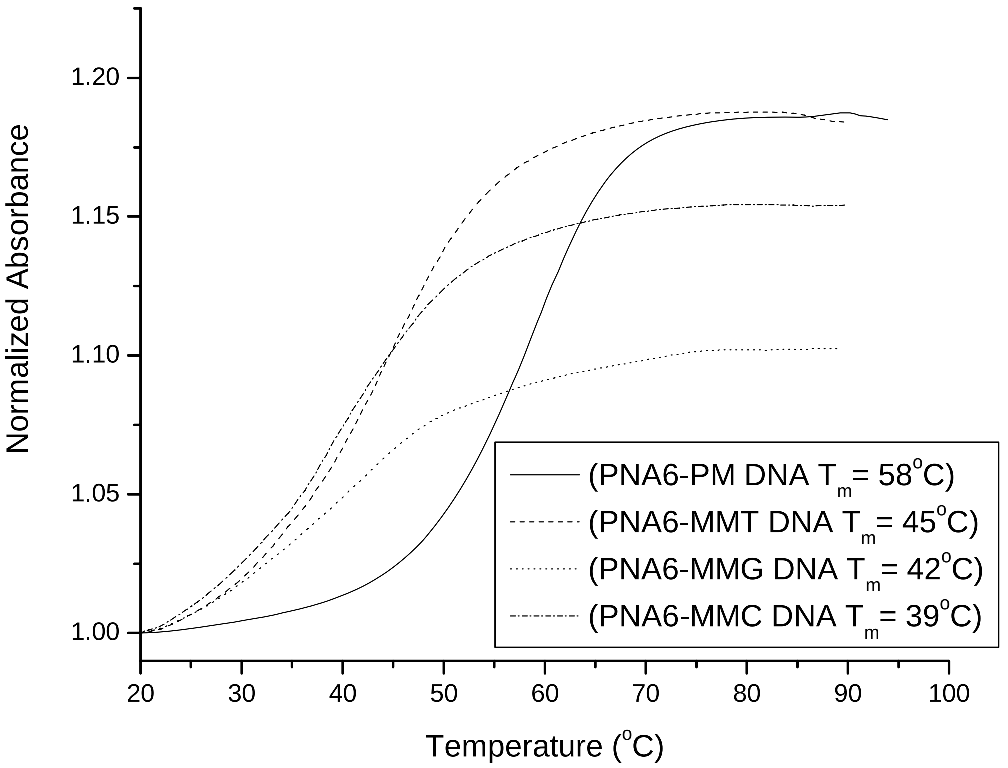

Figure S16. UV-melting curves of PNA6-DNA duplexes containing perfectly matched (PM) and mismatch (MM) sequences. The samples were prepared in buffer containing 0.1 mM EDTA, 100 mM NaCl, 10 mM sodium phosphate (pH 7.0) at 5μM duplex strand concentration each. The T_ms were determined by taking the first derivatives of the UV-melting curves.

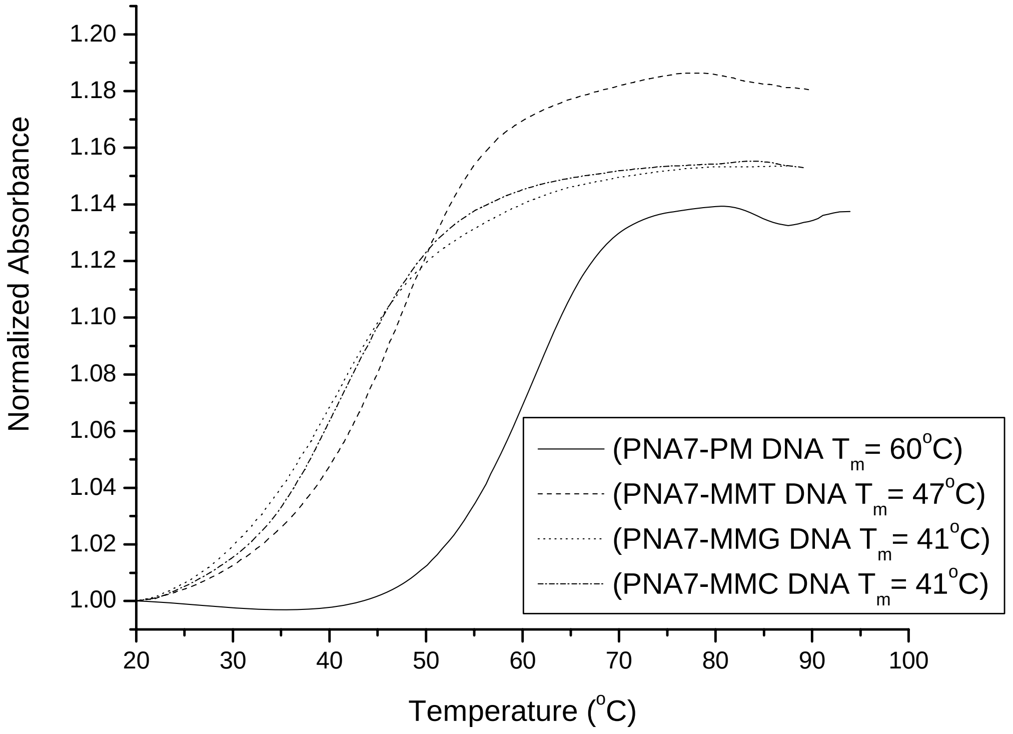

Figure S17. UV-melting curves of PNA7-DNA duplexes containing perfectly matched (PM) and mismatch (MM) sequences. The samples were prepared in buffer containing 0.1 mM EDTA, 100 mM NaCl, 10 mM sodium phosphate (pH 7.0) at 5μM duplex strand concentration each. The T_ms were determined by taking the first derivatives of the UV-melting curves.

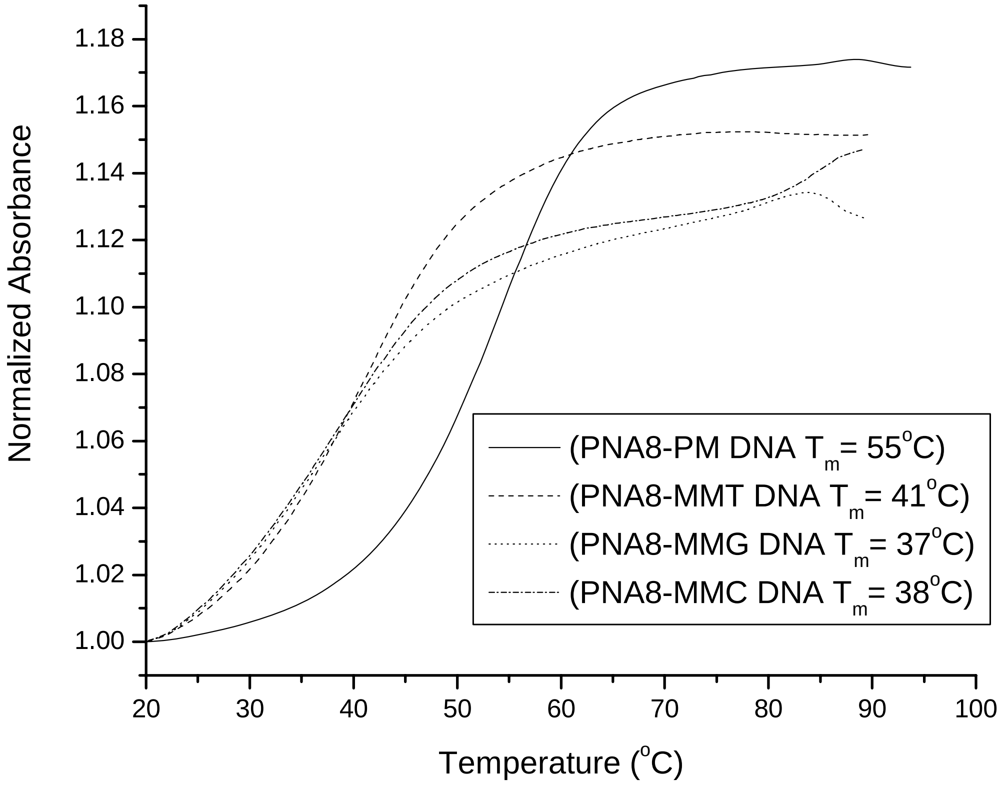

Figure S18. UV-melting curves of PNA8-DNA duplexes containing perfectly matched (PM) and mismatch (MM) sequences. The samples were prepared in buffer containing 0.1 mM EDTA, 100 mM NaCl, 10 mM sodium phosphate (pH 7.0) at 5μM duplex strand concentration each. The T_ms were determined by taking the first derivatives of the UV-melting curves.

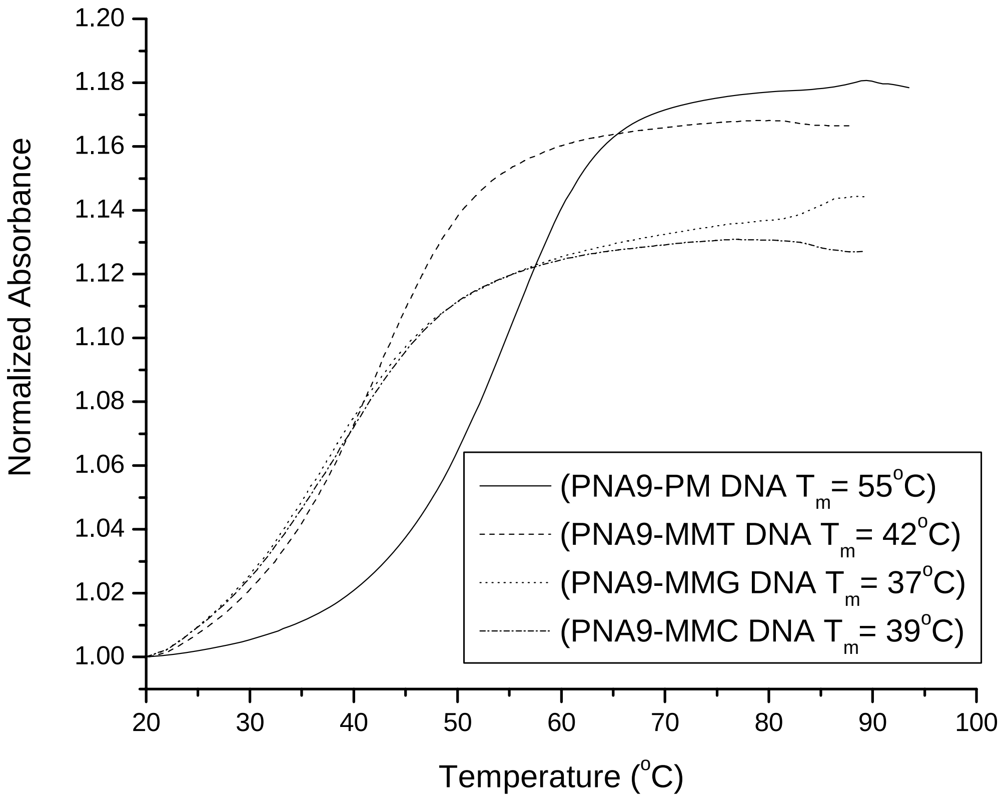

Figure S19. UV-melting curves of PNA9-DNA duplexes containing perfectly matched (PM) and mismatch (MM) sequences. The samples were prepared in buffer containing 0.1 mM EDTA, 100 mM NaCl, 10 mM sodium phosphate (pH 7.0) at 5μM duplex strand concentration each. The T_ms were determined by taking the first derivatives of the UV-melting curves.

^{1}HNMR

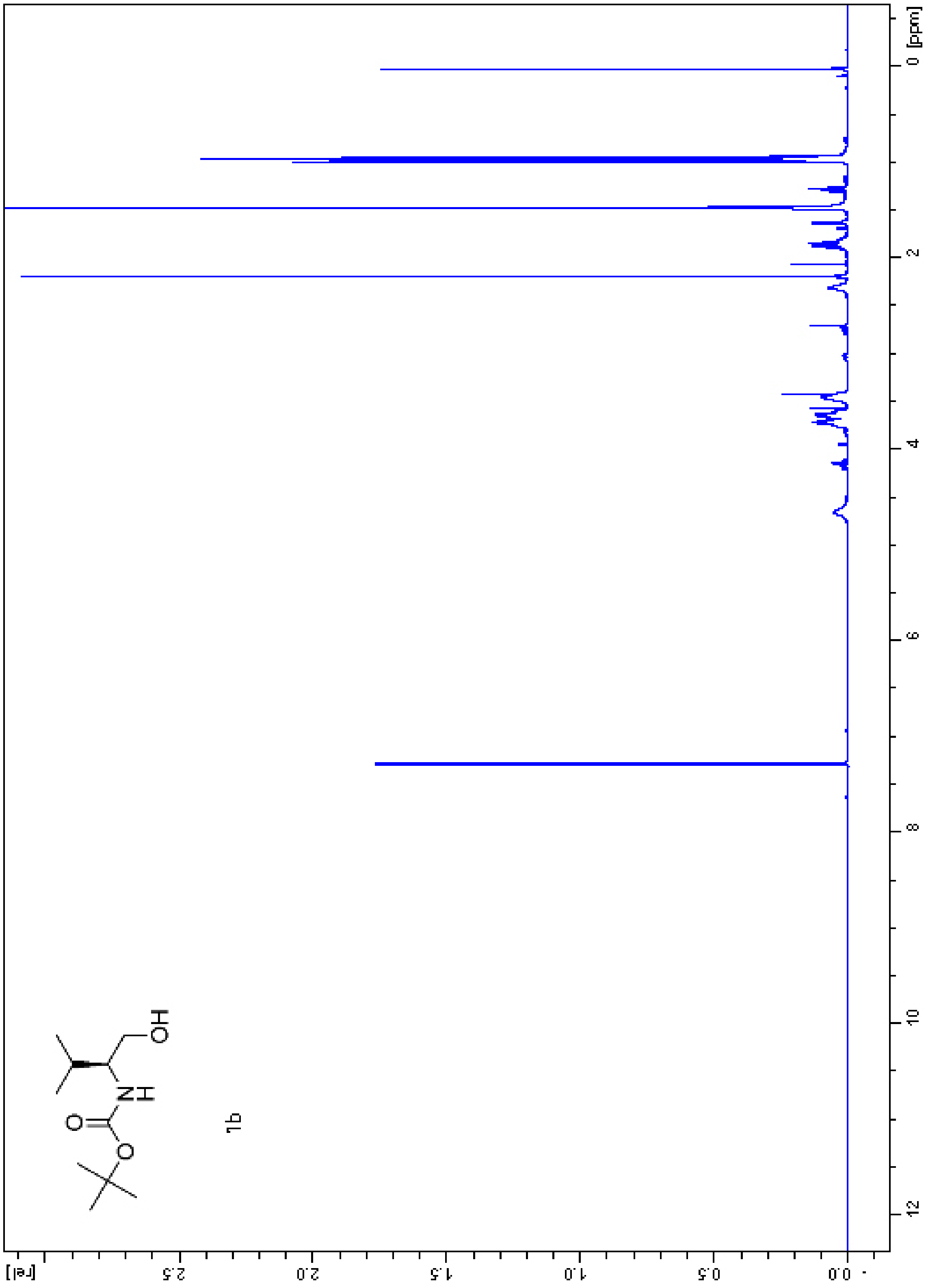

^{1}H NMR

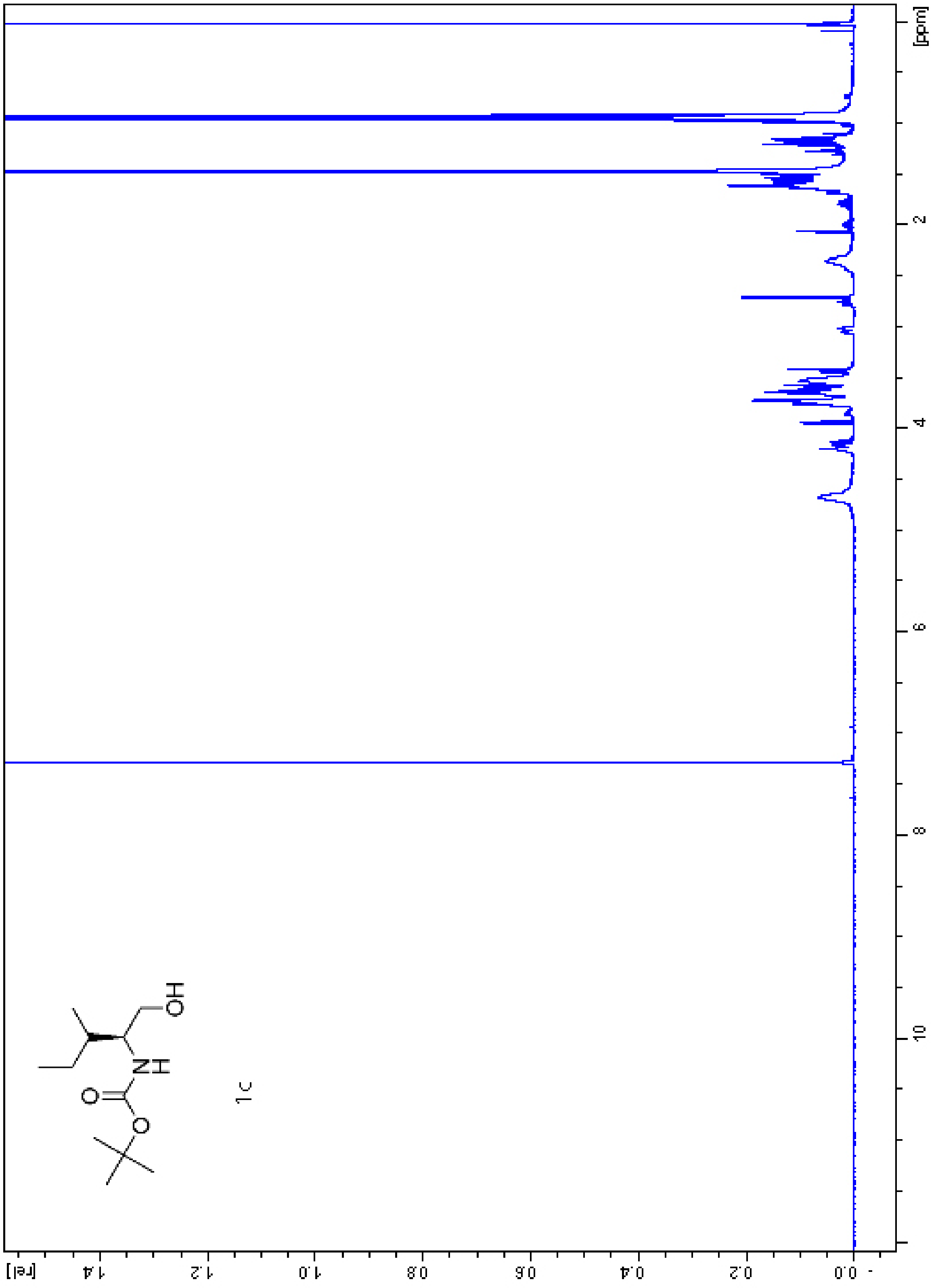

^{1}H NMR

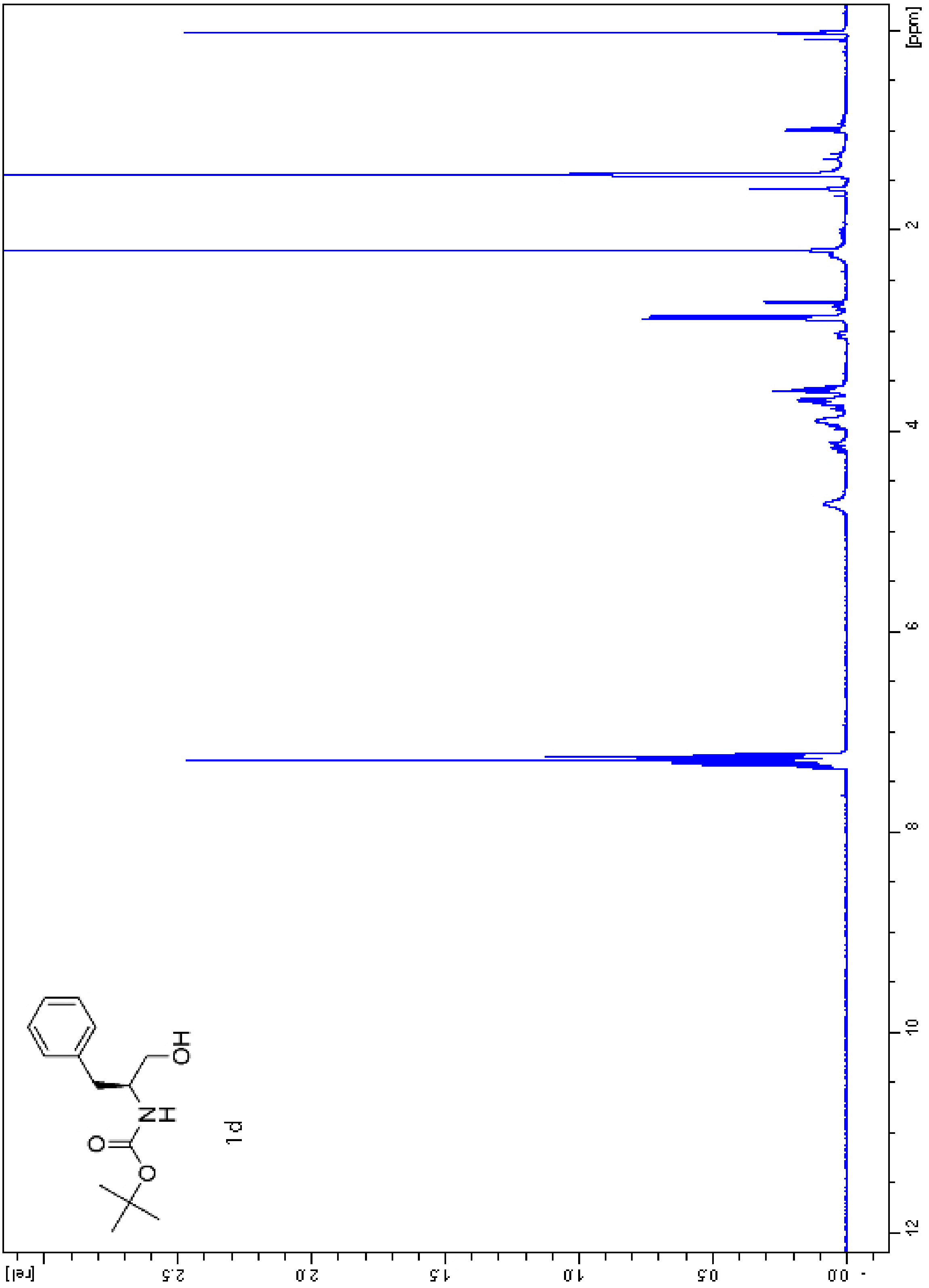

^{1}H NMR

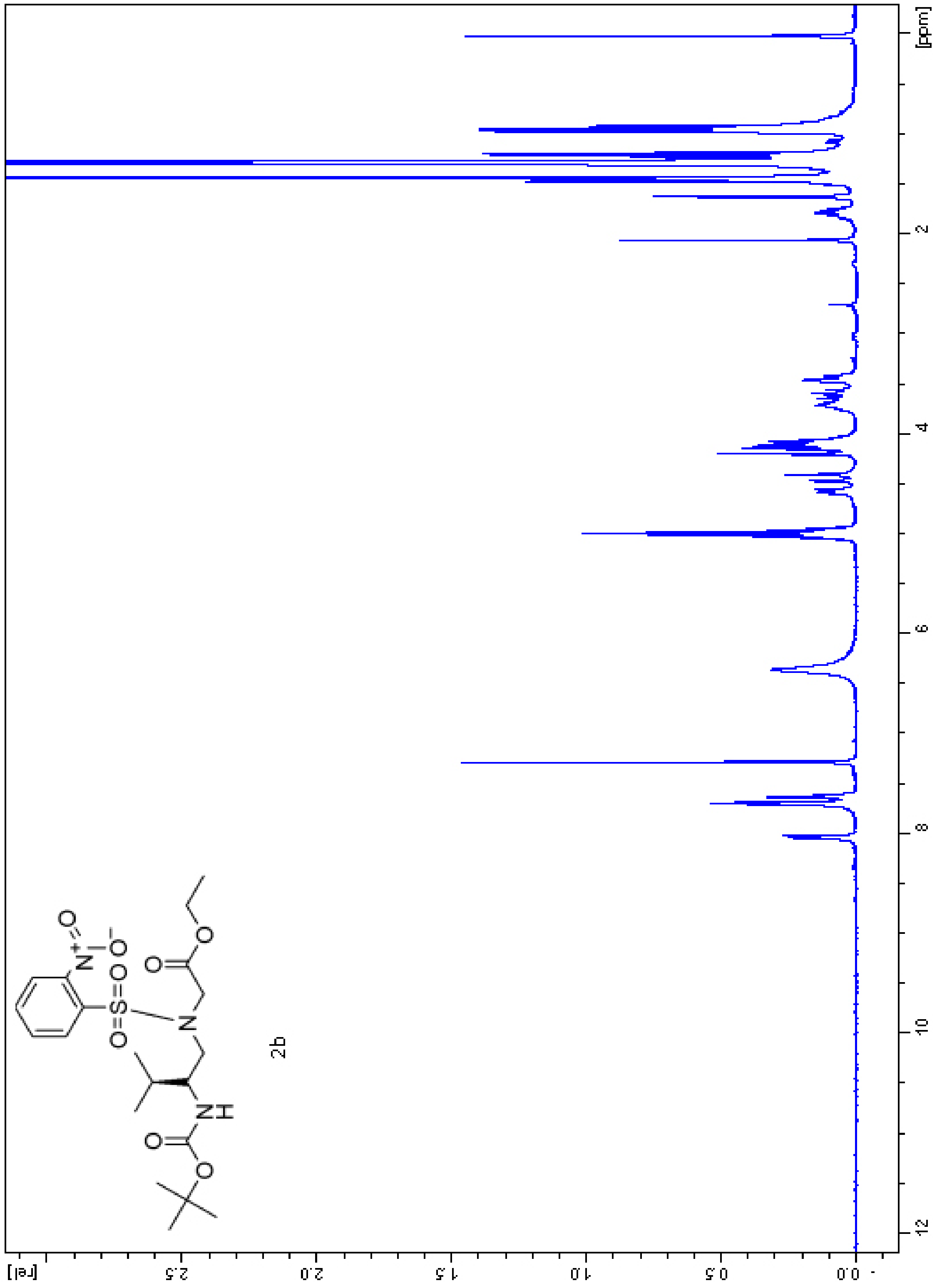

^{1}H NMR

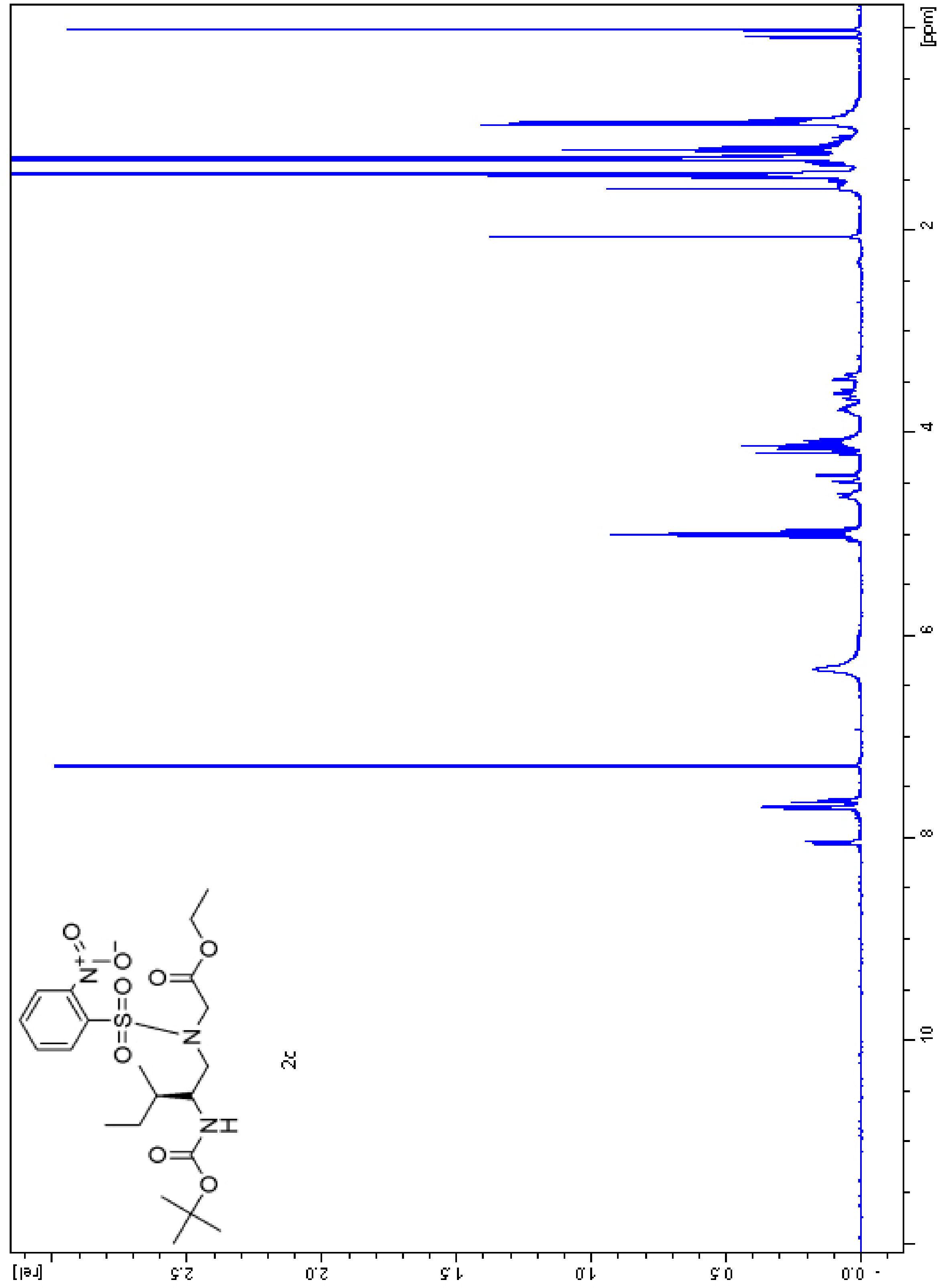

^{1}H NMR

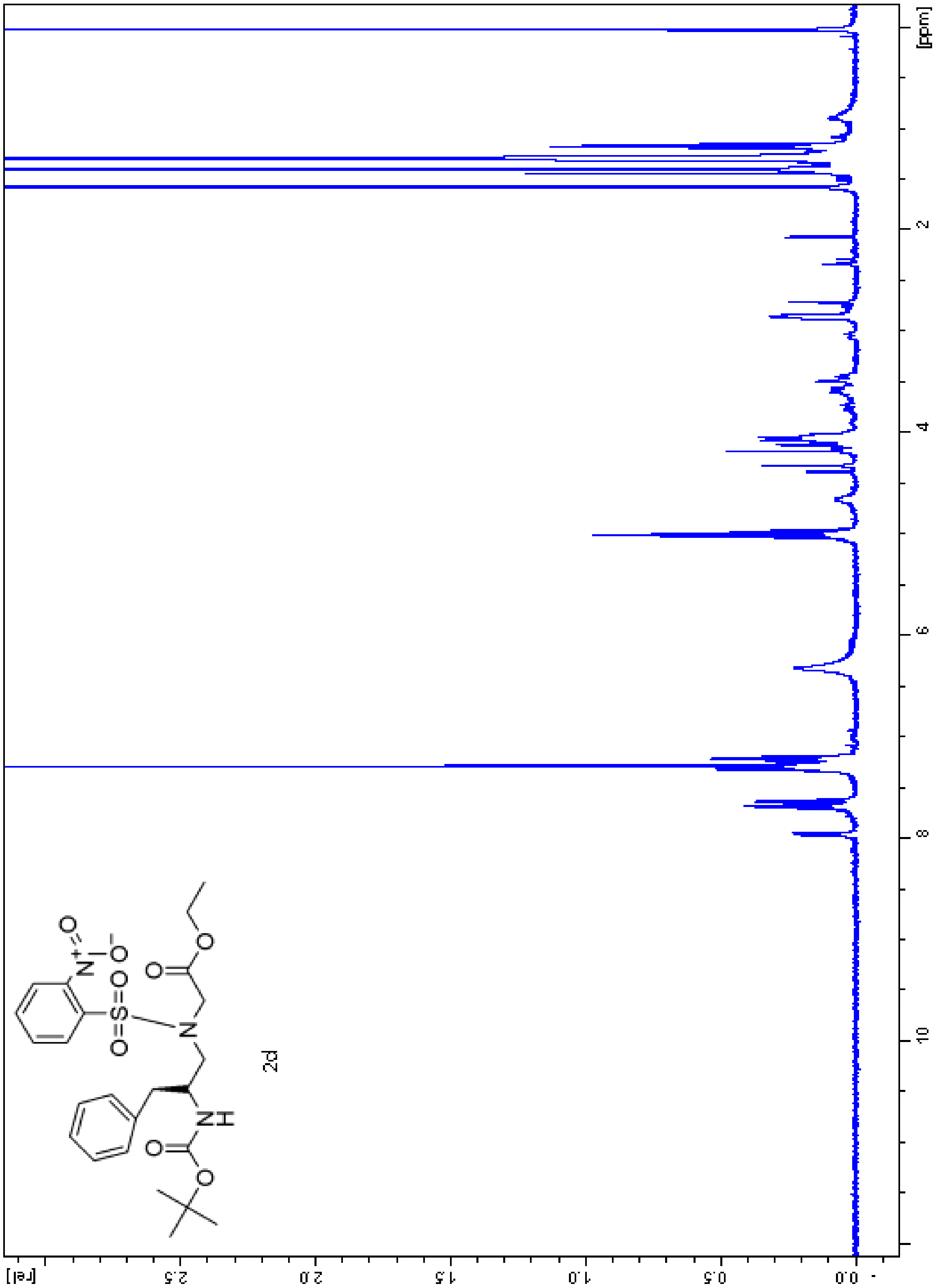

^{1}H NMR

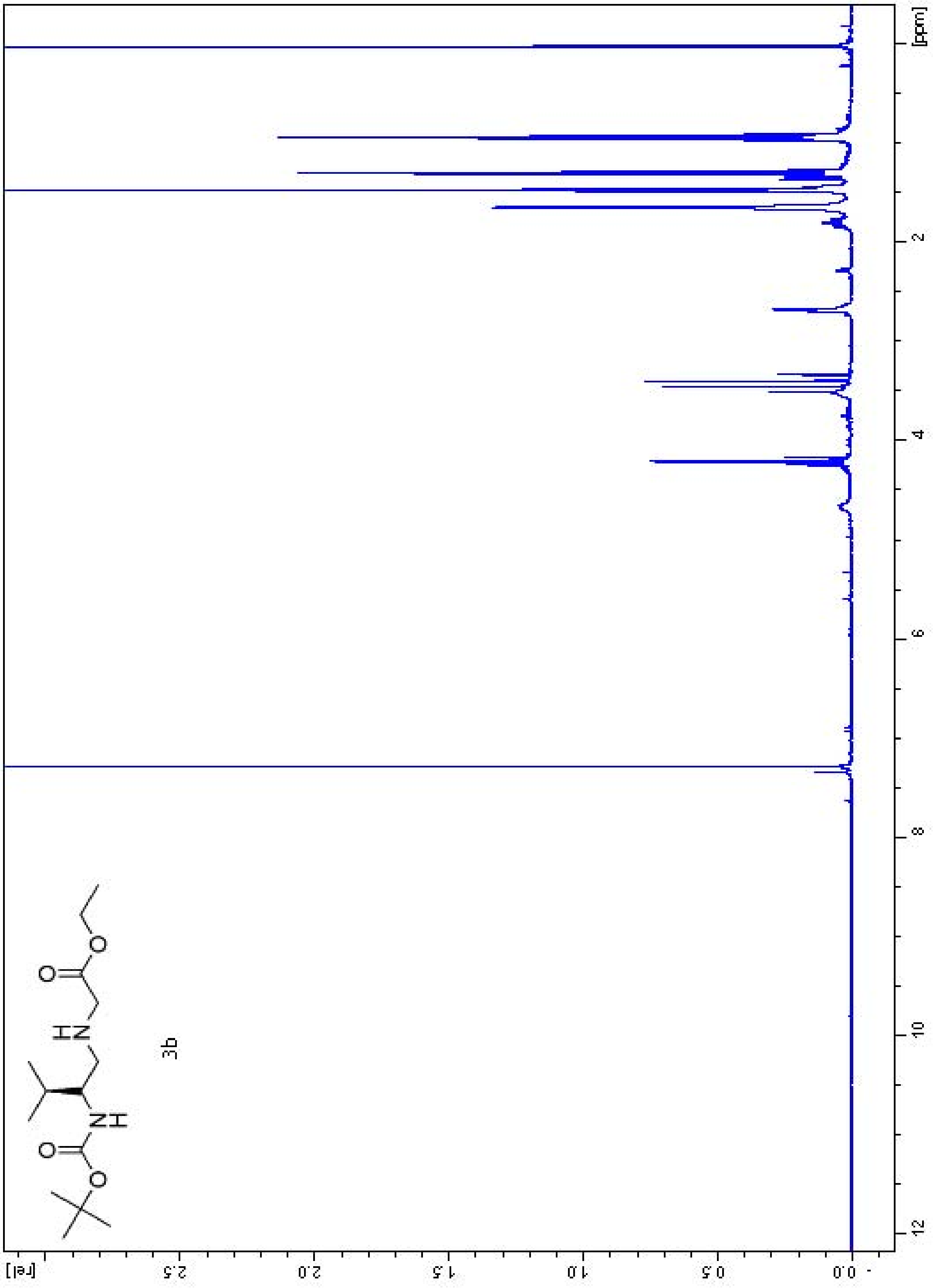

^{13}C NMR

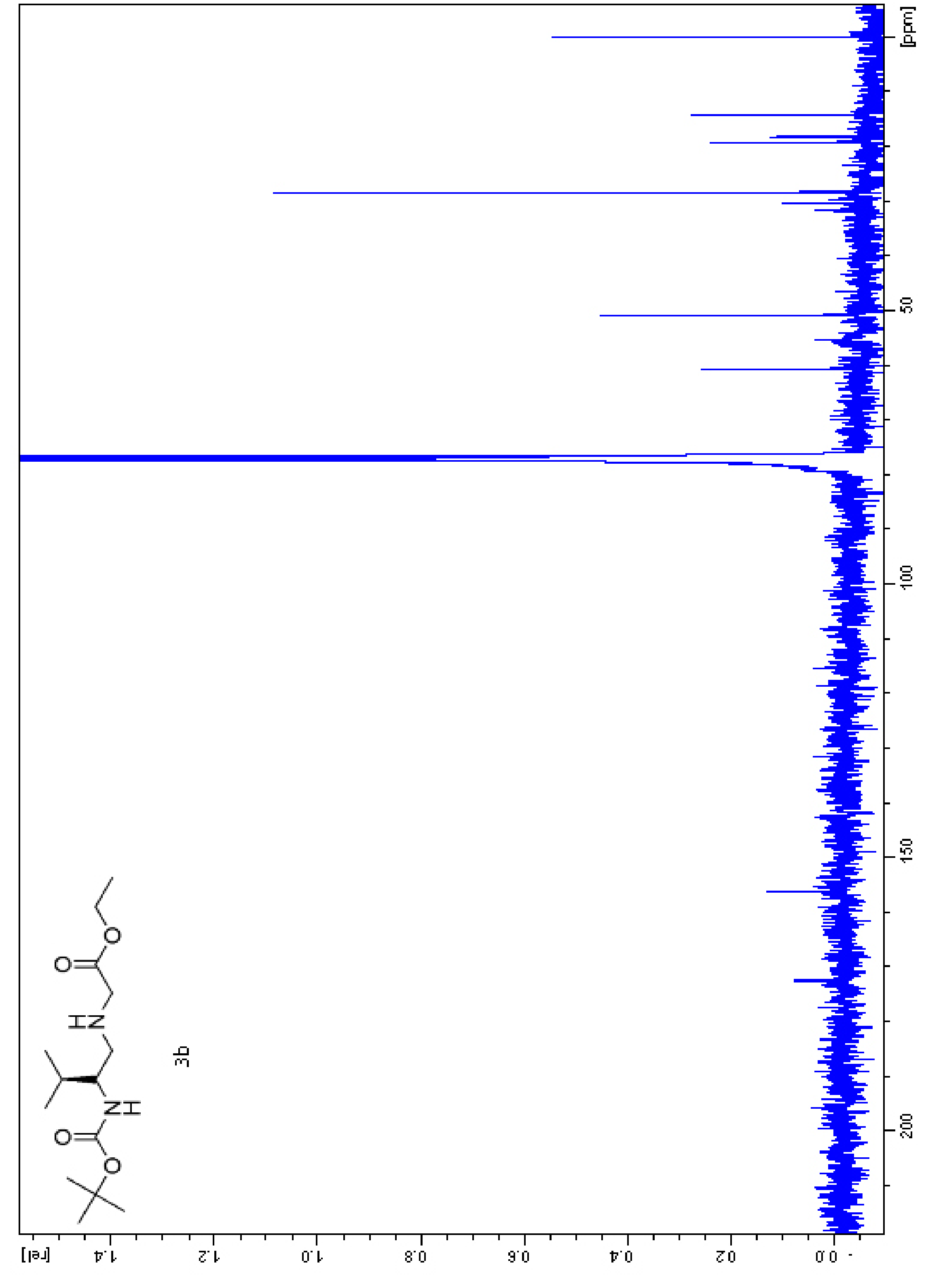

^{1}H NMR

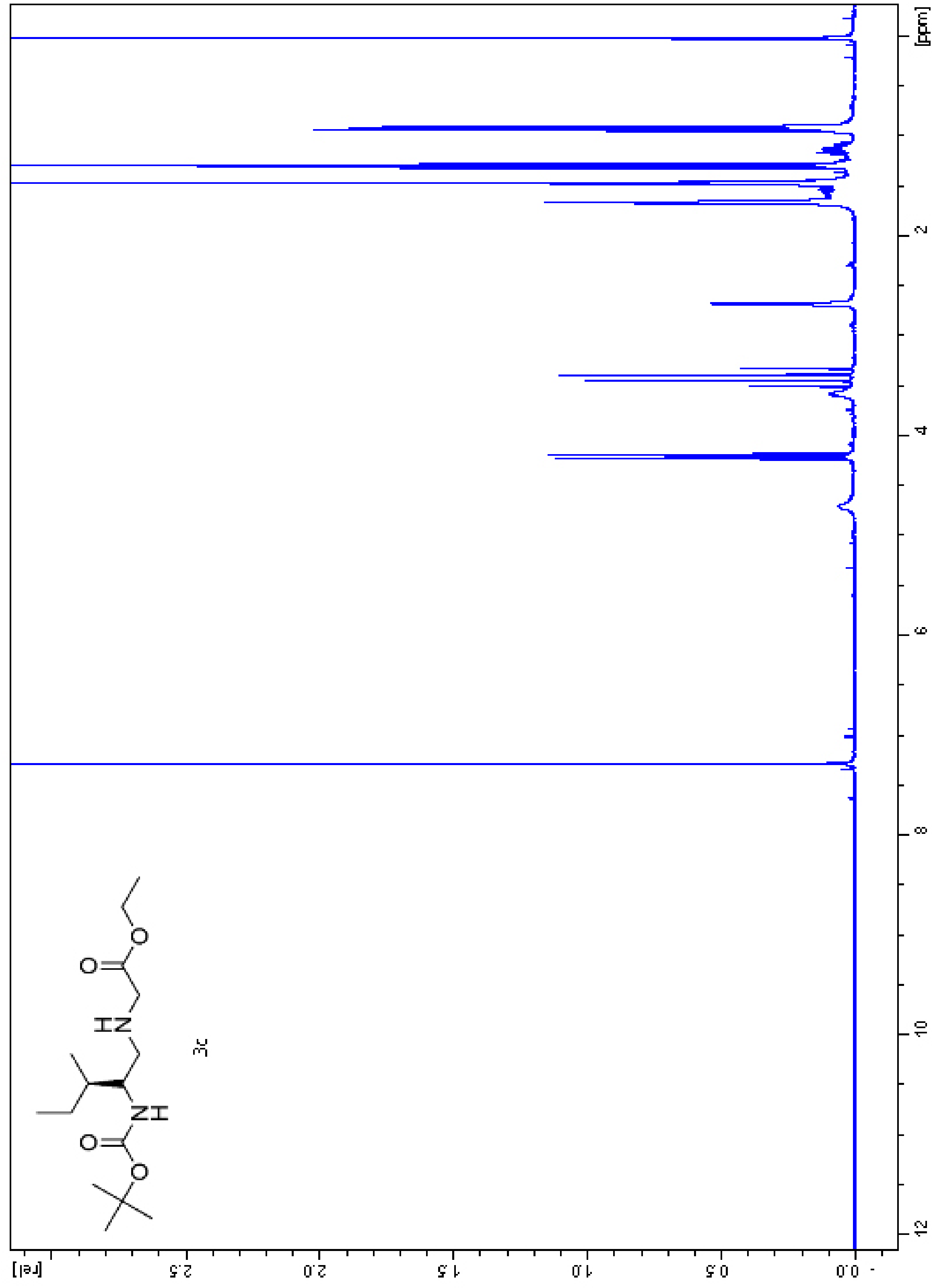

^{13}C NMR

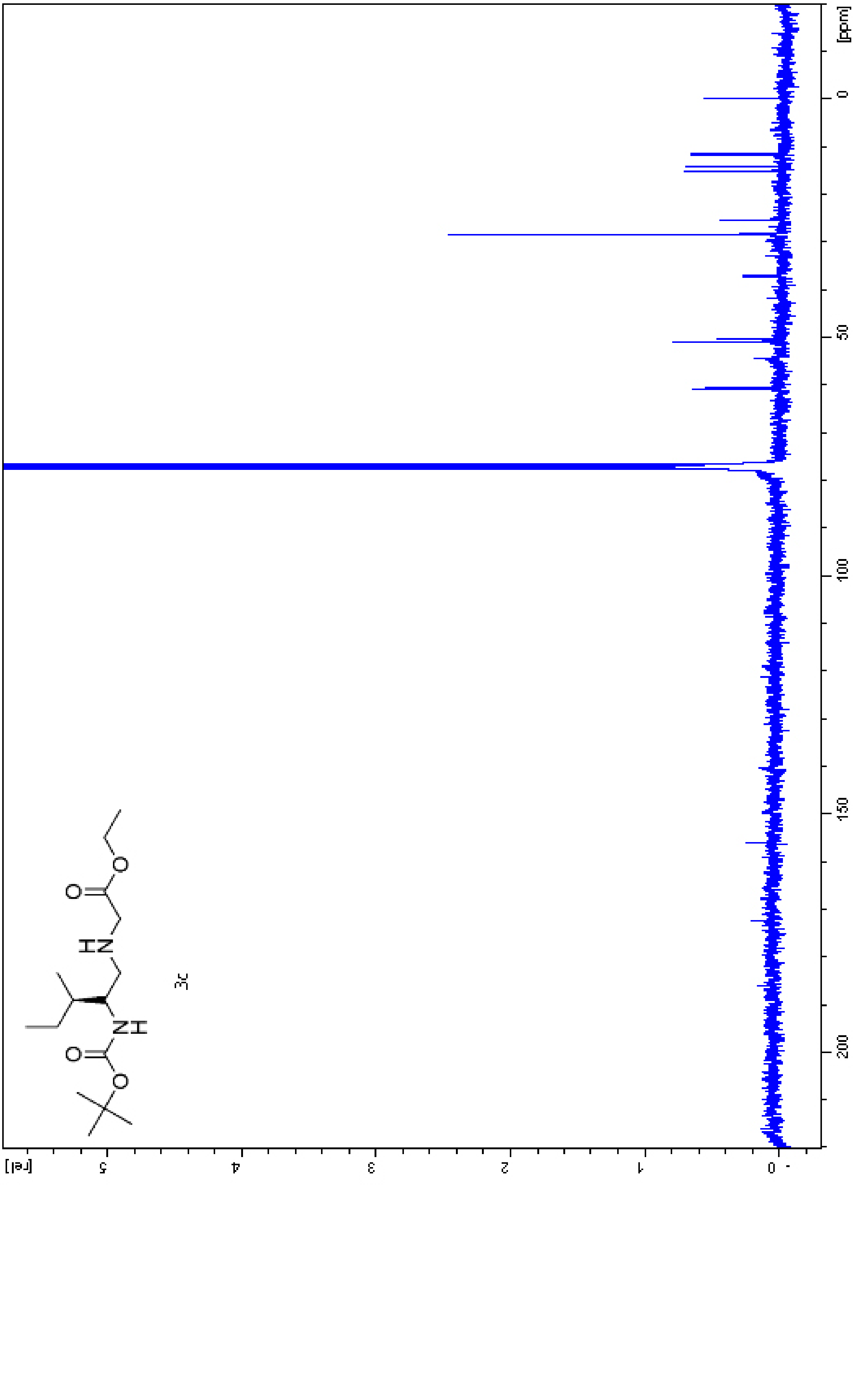

^{1}H NMR

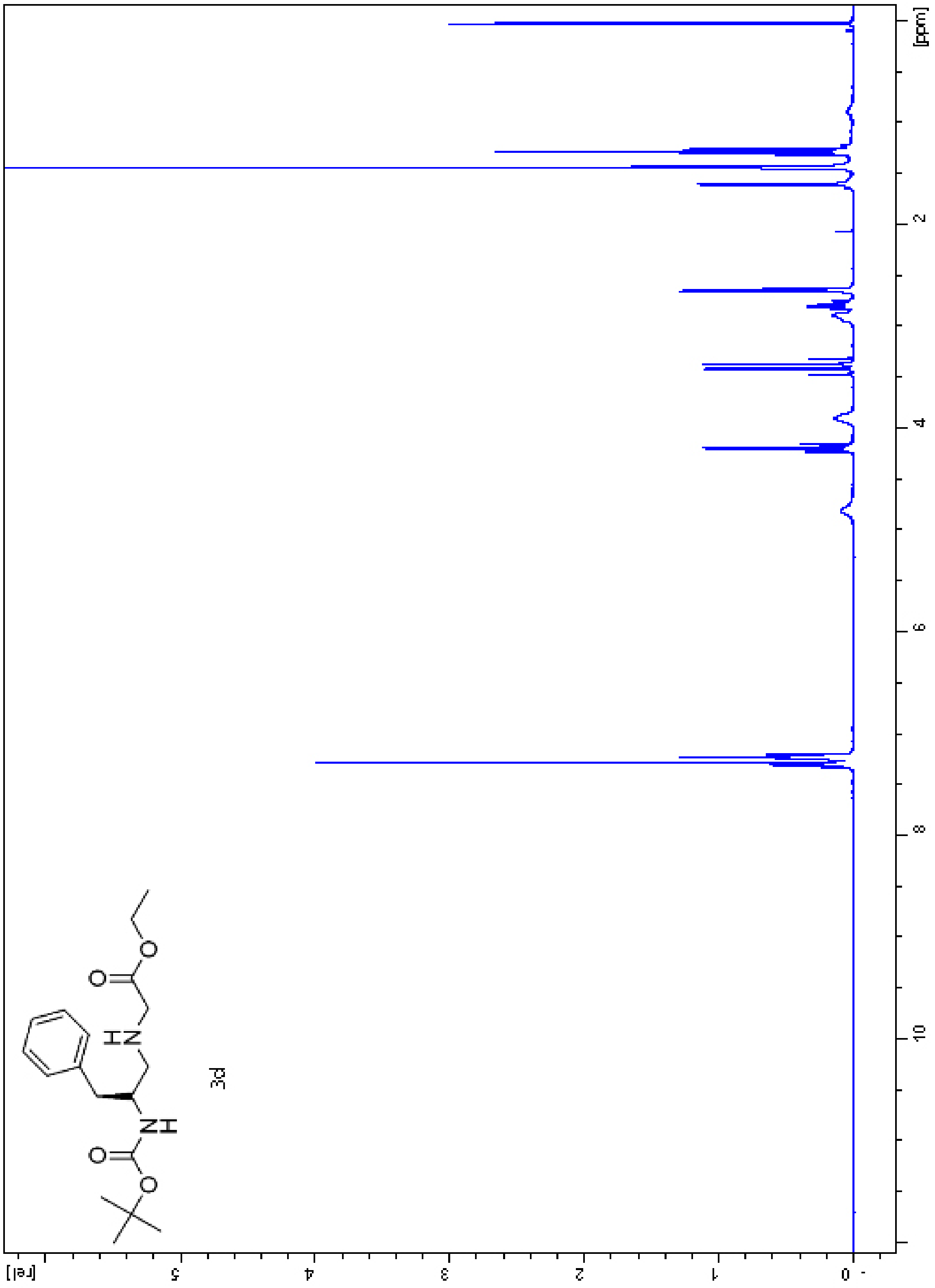

^{13}C NMR

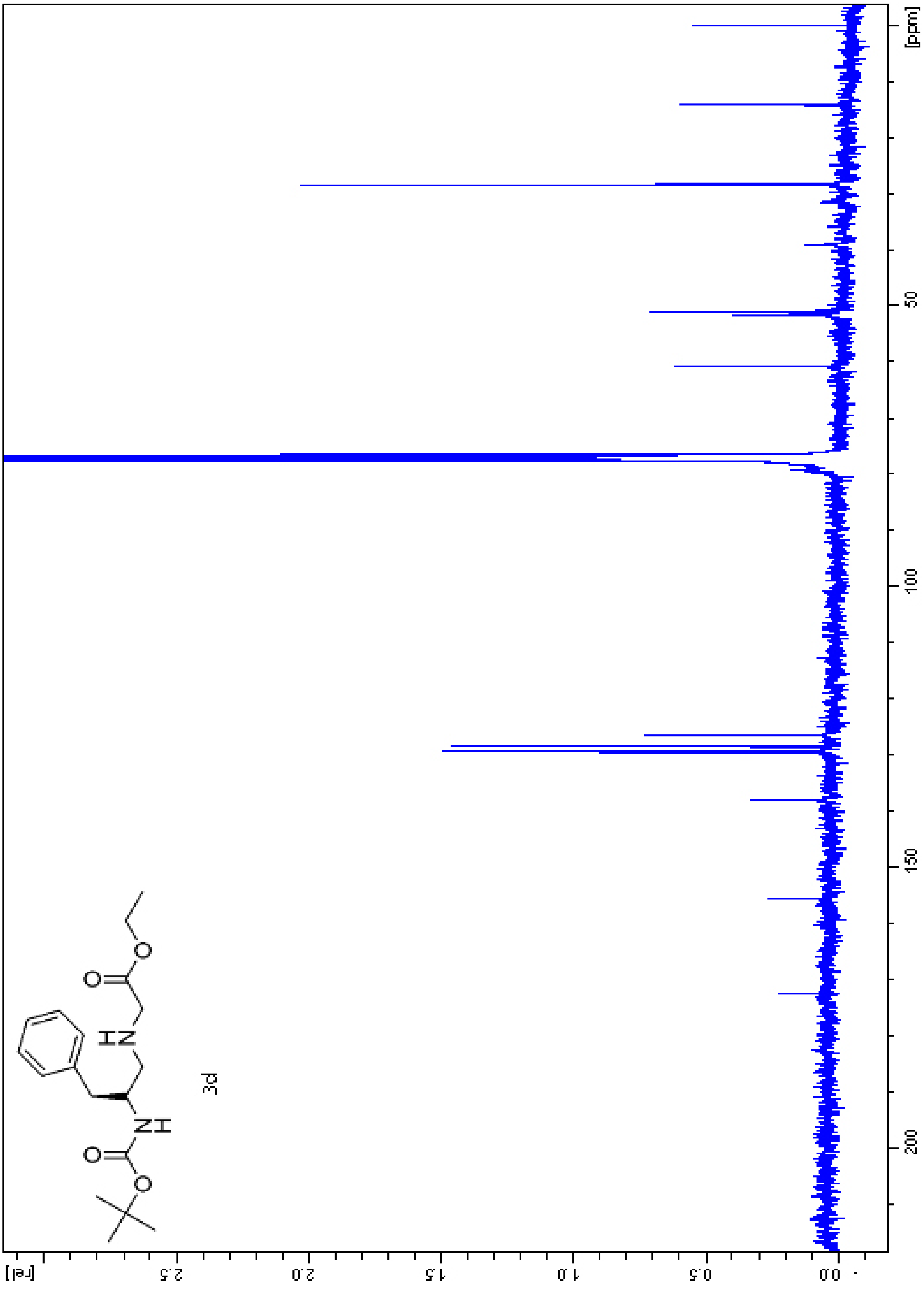

^{1}H NMR

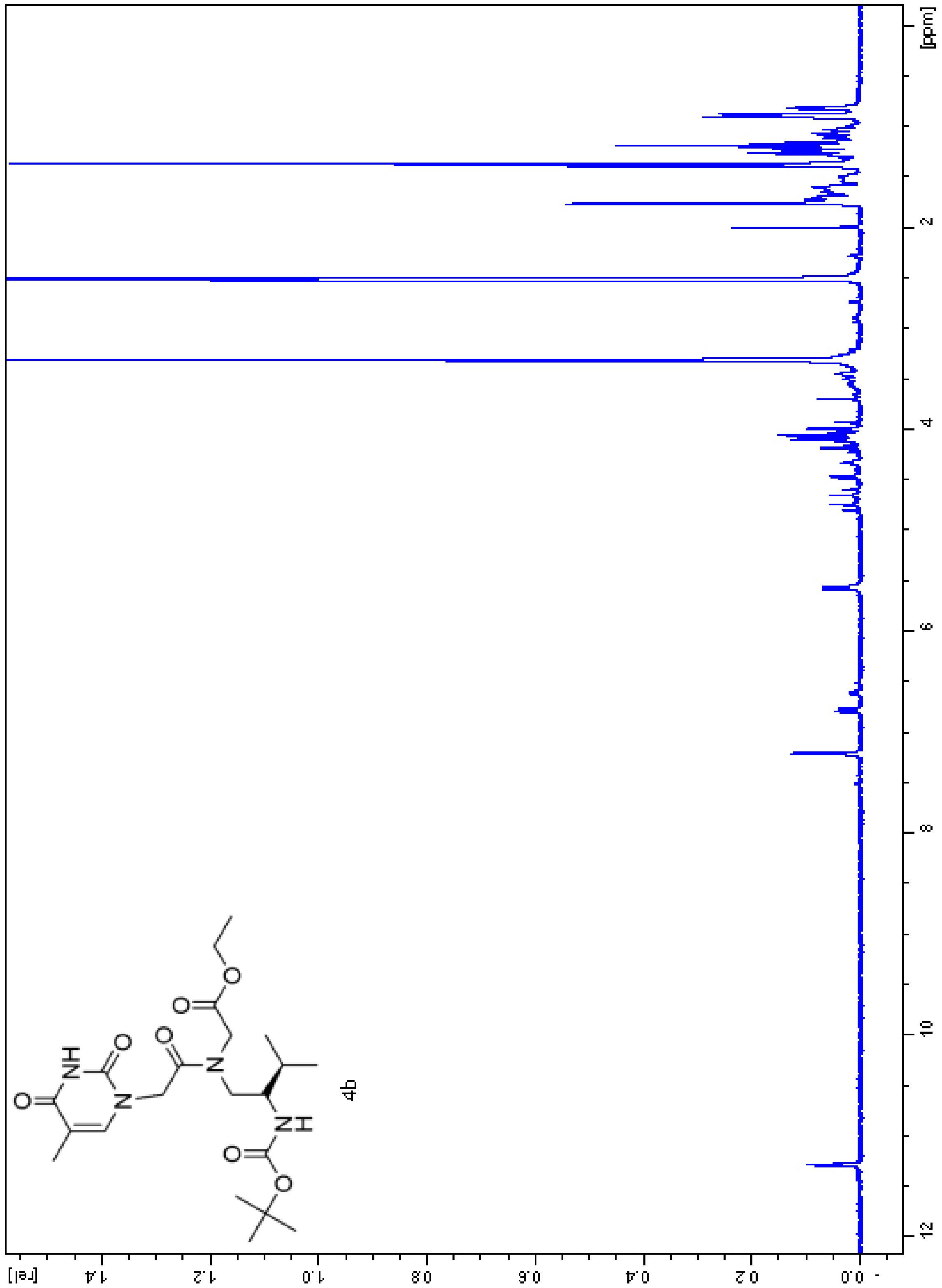

^{1}H NMR

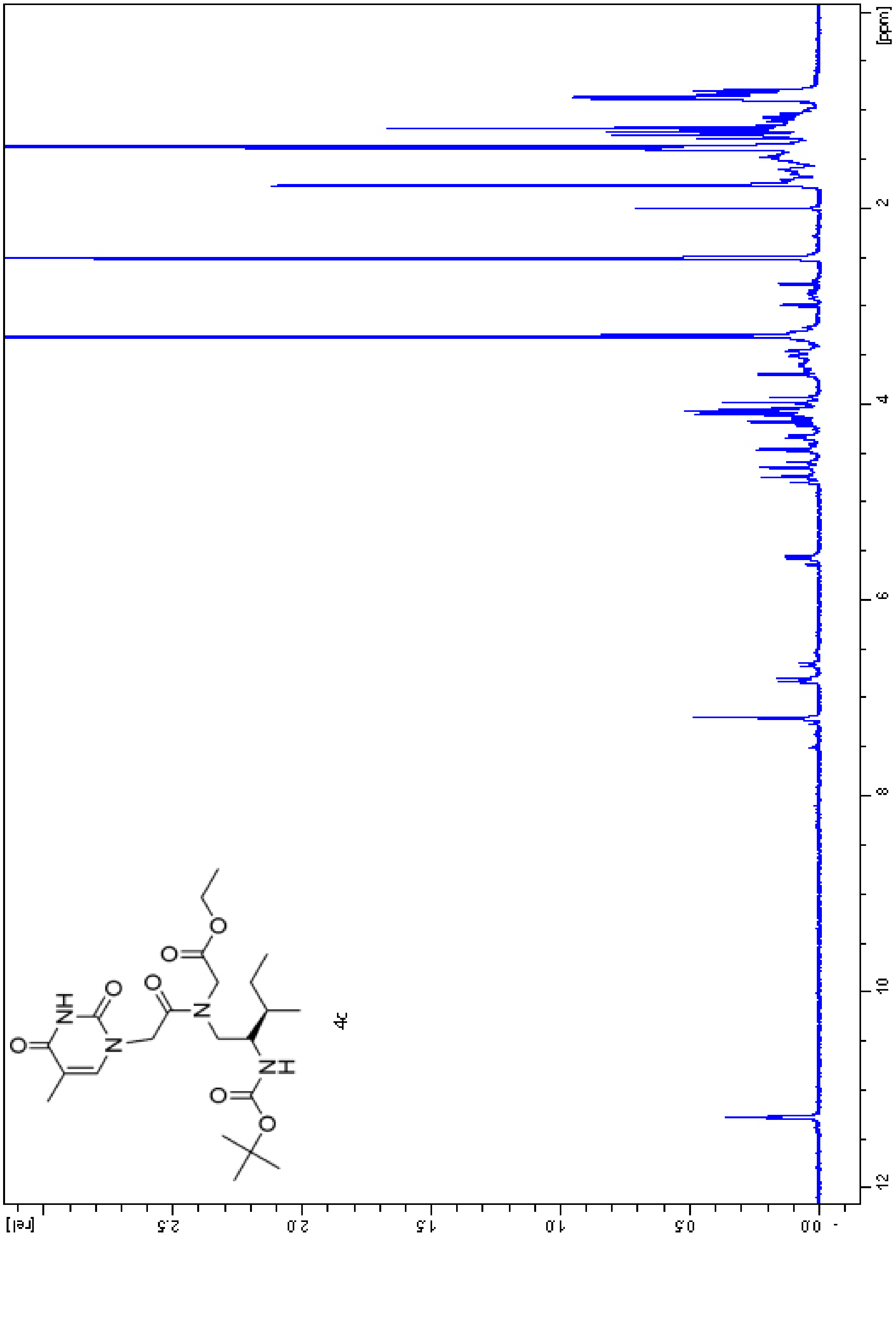

^{1}H NMR

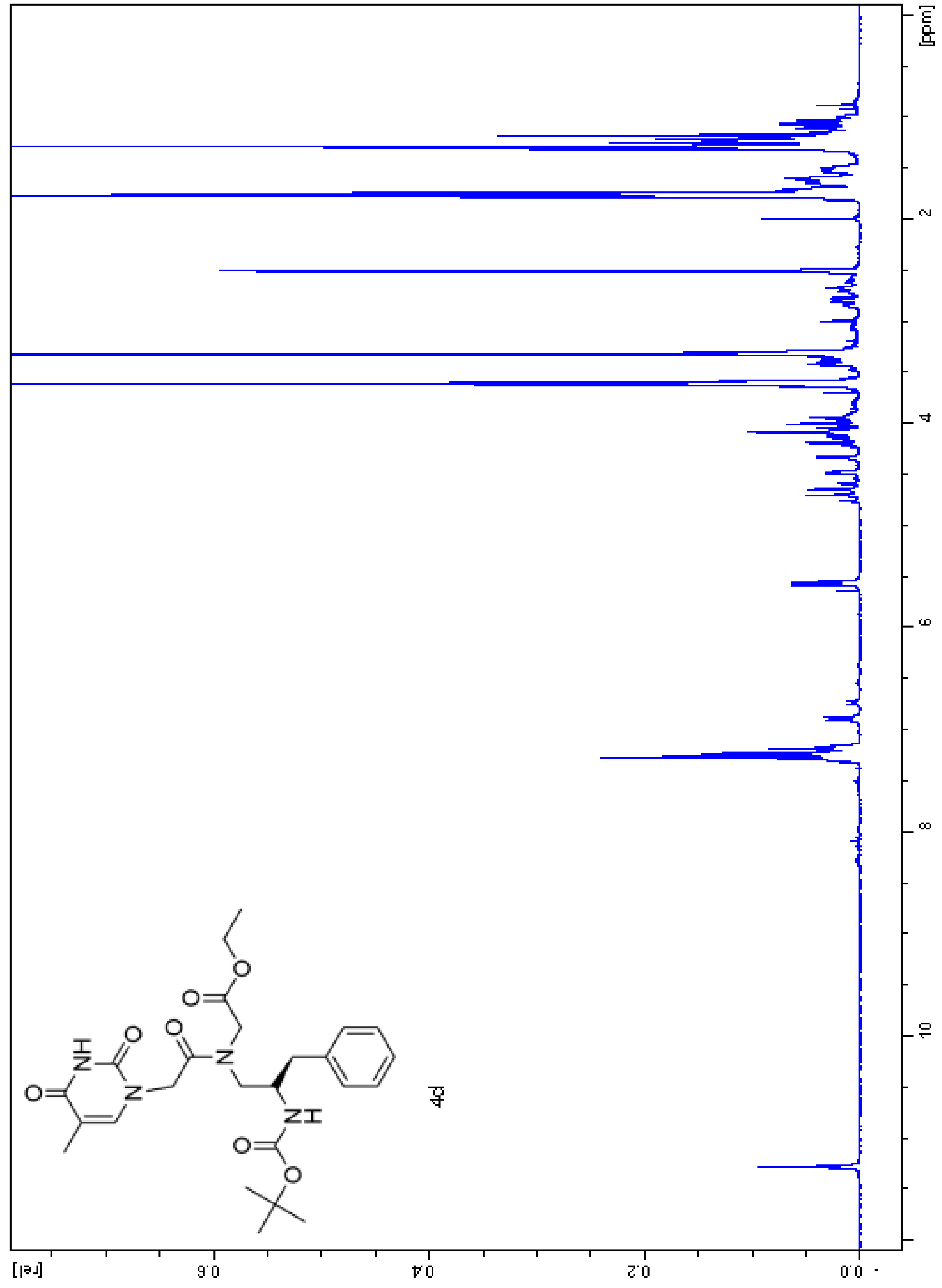

^{1}H NMR

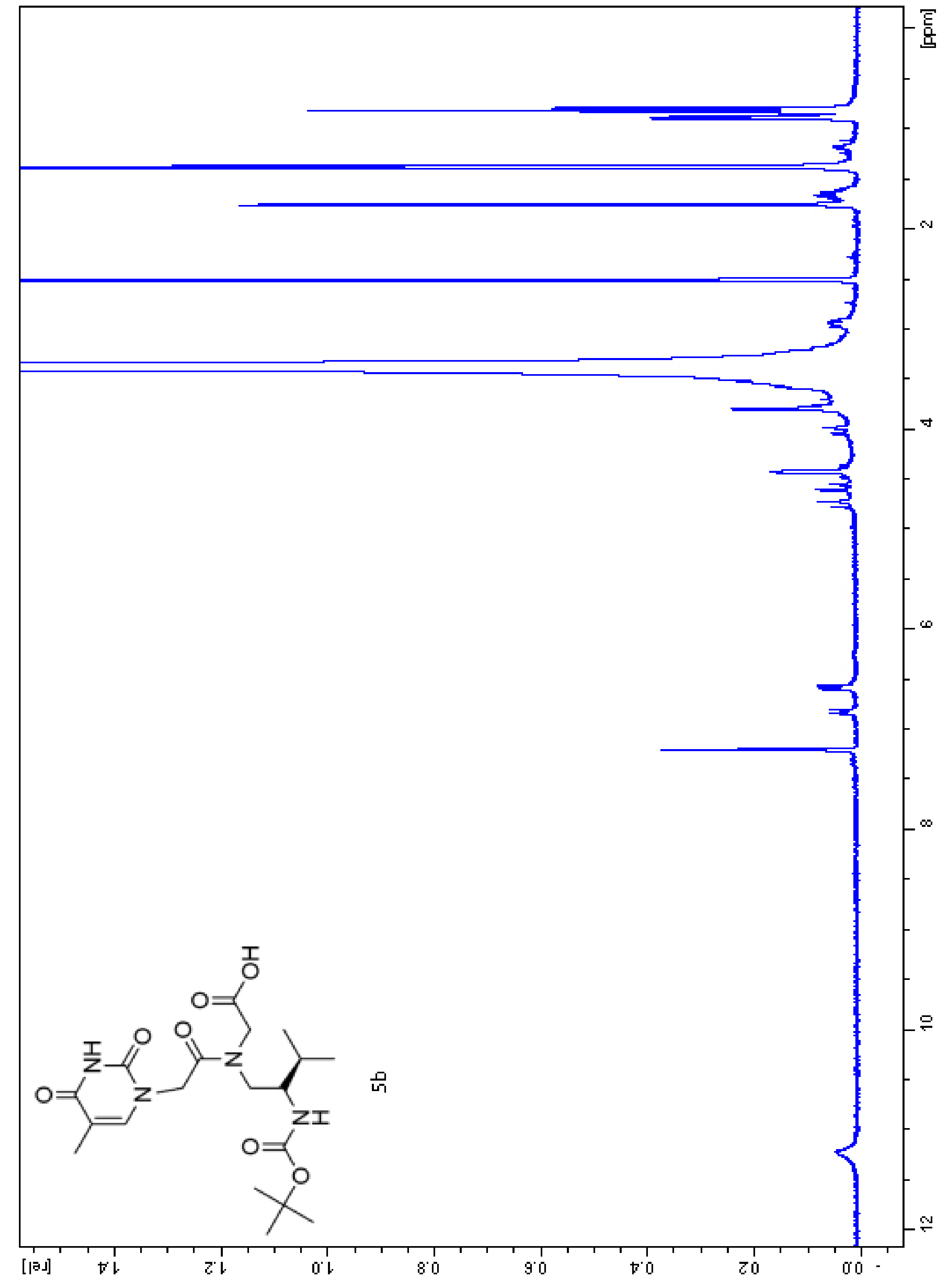

^{13}C NMR

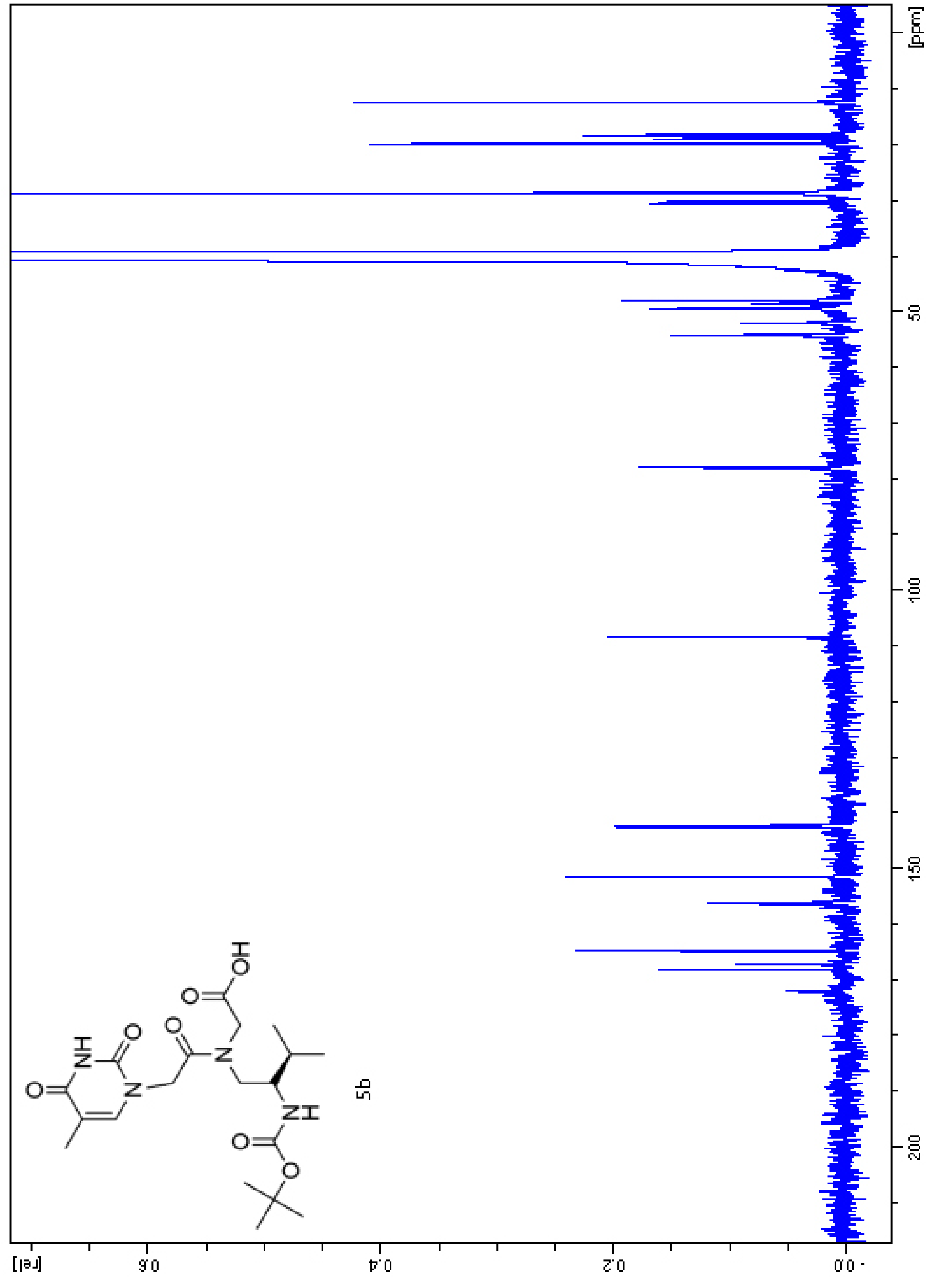

^{1}H NMR

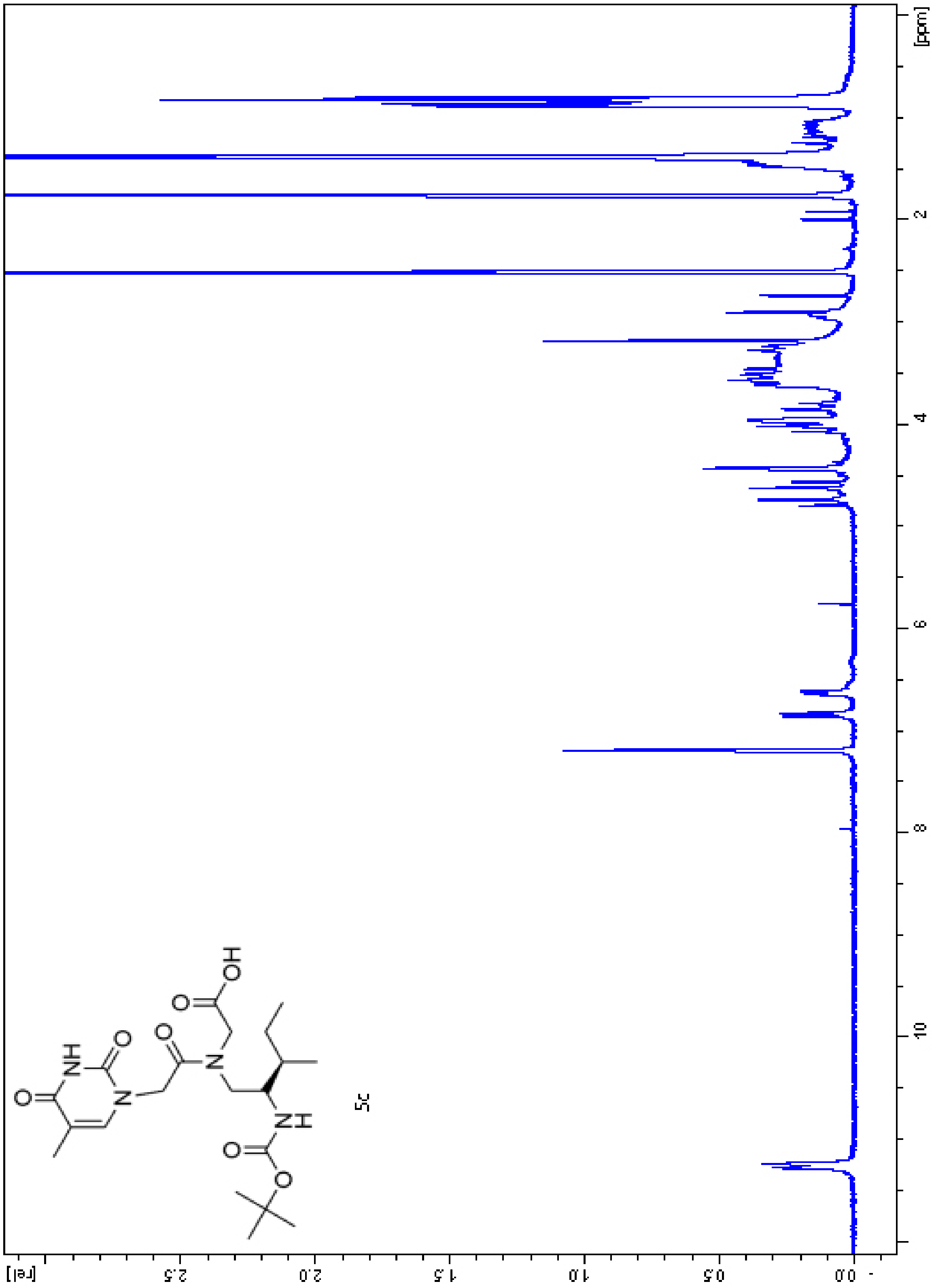

^{13}C NMR

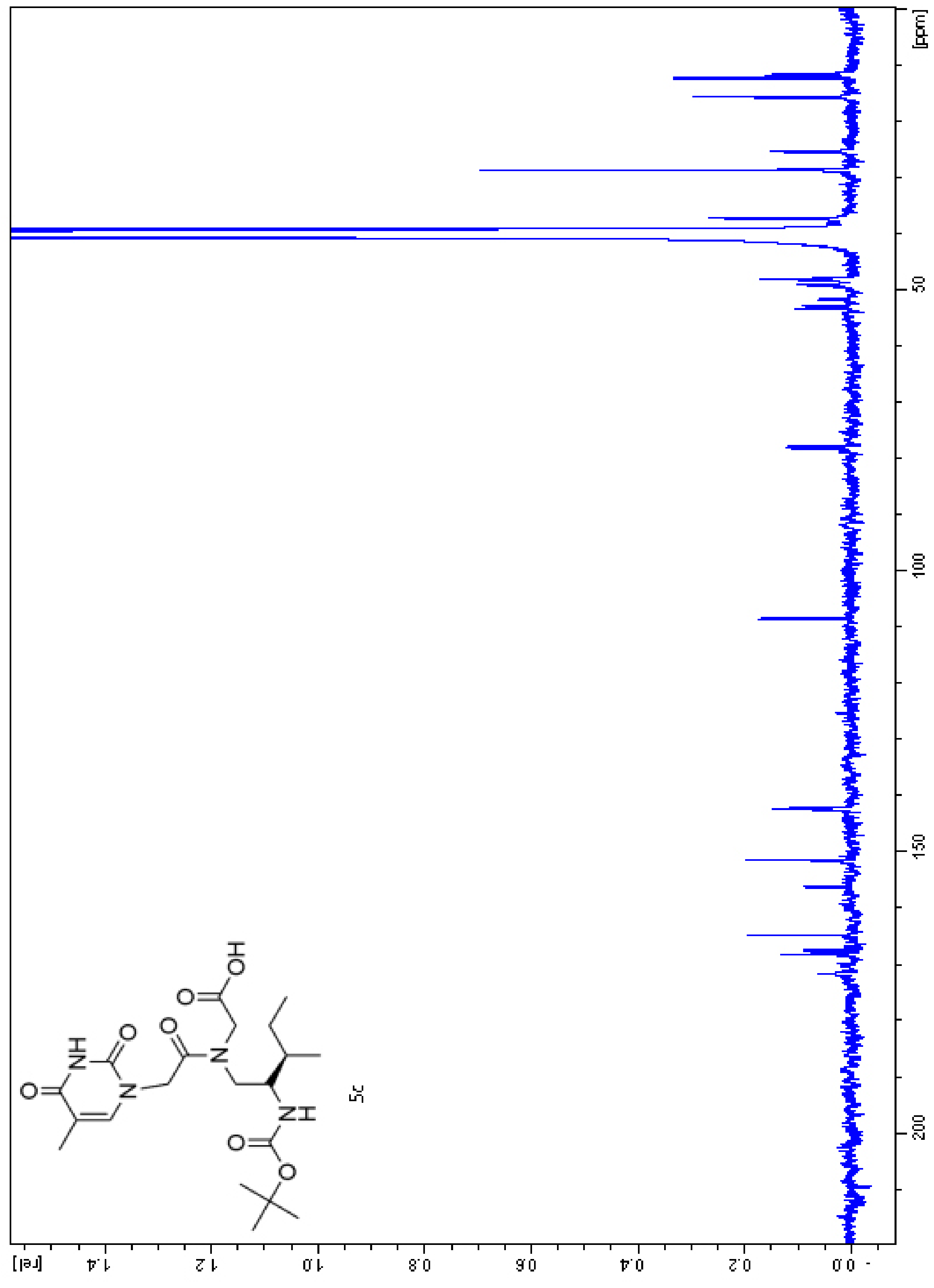

^{1}H NMR

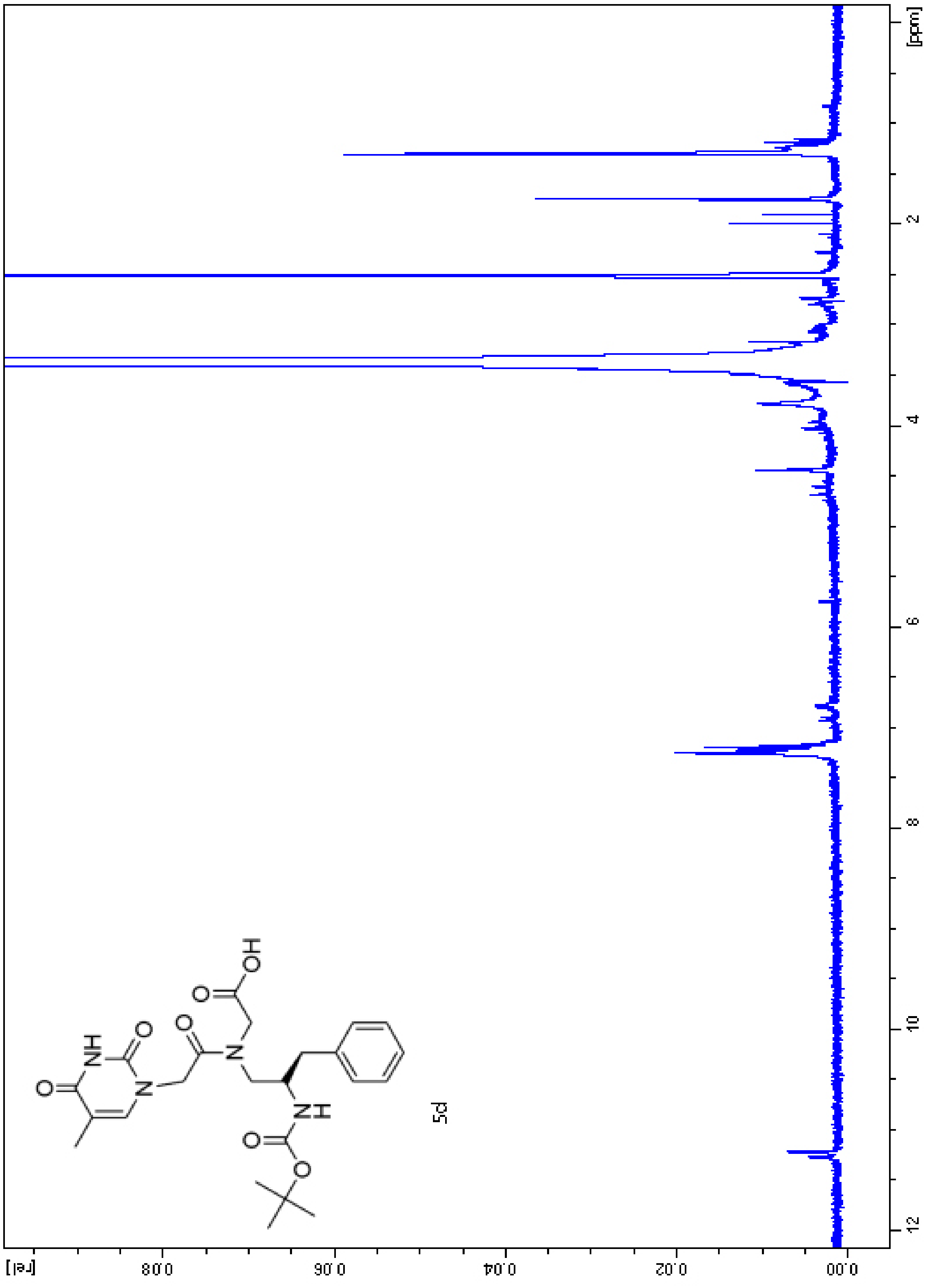

^{13}C NMR

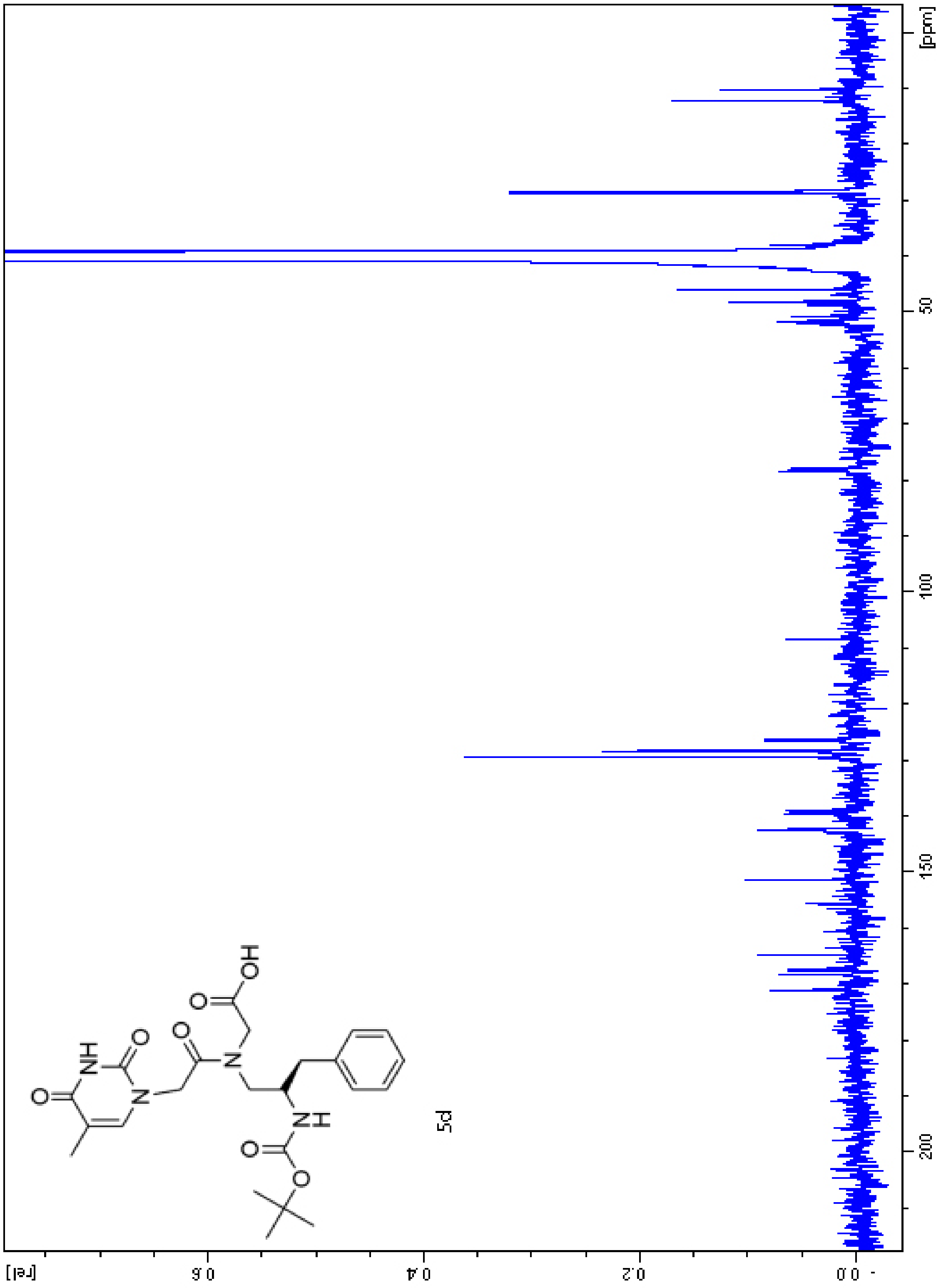

Supplement: Supplementary file 2 [file 652702.f2.pdf]
